# Data on prior pegylated liposomal doxorubicin (PLD) treatment in recurrent ovarian cancer: Post-hoc data analysis from the phase 3 randomized, open-label study comparing trabectedin and PLD versus PLD alone in patients with recurrent ovarian cancer

**DOI:** 10.1016/j.dib.2020.105465

**Published:** 2020-03-20

**Authors:** Bradley J. Monk, Thomas J. Herzog, George Wang, Spyros Triantos, Scott Maul, Roland Knoblauch, Tracy McGowan, Waleed S.W. Shalaby, Robert L. Coleman

**Affiliations:** a Arizona Oncology (US Oncology Network), University of Arizona, Creighton University, Phoenix, AZ, United States; b University of Cincinnati Cancer Center, University of Cincinnati, Cincinnati, OH, United States; c Janssen Research & Development, Spring House, PA, United States; d Janssen Research & Development, Los Angeles, CA, United States; e Janssen Scientific Affairs, LLC, Horsham, PA, United States; f The University of Texas MD Anderson Cancer Center, Houston, TX, United States

**Keywords:** Overall survival, Pegylated liposomal doxorubicin, Trabectedin, Recurrent ovarian cancer, Response rate

## Abstract

The data presented herein are supplementary to our published primary article “A phase 3 randomized, open-label, multicenter trial for safety and efficacy of combined trabectedin and pegylated liposomal doxorubicin therapy for recurrent ovarian cancer”[1]. The exploratory analysis evaluated the impact of prior pegylated liposomal doxorubicin (PLD) therapy in patients who participated in a randomized, open-label study comparing combination therapy of trabectedin and PLD vs PLD alone in third-line recurrent ovarian cancer (ROC). These exploratory analyses showed that prior treatment with PLD in ROC does not impact the response and survival rates nor does it increase toxicities or negatively influence survival and response rates in both treatment groups.

Specifications table

**Table:** 

Subject	Medicine and Dentistry
Specific subject area	Oncology
Type of data	Tables and Figures
How data were acquired	Data were obtained from scheduled clinical assessments and adverse event monitoring. Case report forms (CRF) for each patient were captured by study-site personnel from the source documents onto an electronic CRF (Electronic Data Capture).
Data format	Raw, analyzed and descriptive data
Parameters for data collection	Data were collected and analyzed according to prior PLD therapy. Described in the Statistical Analysis Plan available in a public repository: https://clinicaltrials.gov/ProvidedDocs/11/NCT01846611/SAP_001.pdf
Description of data collection	In the phase 3, randomized, open-label active-controlled study, women patients with platinum-sensitive advanced-relapsed epithelial ovarian, primary peritoneal, or fallopian tube cancer were stratified based on their prior PLD therapy. This subgroup analysis examined the safety and efficacy endpoints including overall survival, progression-free survival and objective response rate in platinum-sensitive patients with prior PLD therapy who participated in this study comparing combination therapy of trabectedin and PLD vs PLD alone in third line setting of recurrent ovarian cancer.
Data source location	Data were collected at 117 sites in 10 countries: United States (59 sites), Russian Federation (21 sites), Australia (8 sites), Israel (8 sites), United Kingdom (7 sites), China (5 sites), South Africa (4 sites); New Zealand (2 sites); Poland (2 sites); Switzerland (1 sites).
Data accessibility	Repository name: ClinicalTrials.gov Data identification number: NCT01846611 Direct URL to data: https://clinicaltrials.gov/ct2/show/results/NCT01846611?term=ovc3006&draw=2&rank=1
Related research article	Monk BJ, Herzog TJ, Wang G, et al. A phase 3 randomized, open-label, multicenter trial for safety and efficacy of combined trabectedin and pegylated liposomal doxorubicin therapy for recurrent ovarian cancer. Gynecol Oncol. 2020;156(3):535-544. https://doi.org/10.1016/j.ygyno.2019.12.043

## Value of the data

• The data from this pre-stratified exploratory analysis provide insights on the potential benefits of pegylated liposomal doxorubicin (PLD) as retreatment in combination with trabectedin vs monotherapy in patients with recurrent ovarian cancer (ROC).
• Clinicians and researchers in oncology and other allied fields may find the data useful to improve patients’ outcome in ROC setting.
• Additional statistical analyses can be performed, or the study can be reproduced for further research in this clinical setting.

## 1 Data description

**Fig. 1:**
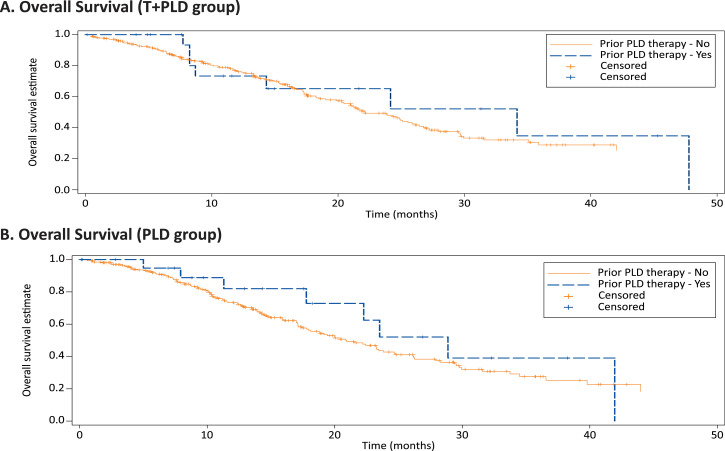
Kaplan–Meier estimates of overall survival by prior PLD use in T+PLD (A) and PLD alone (B).

**Fig. 2:**
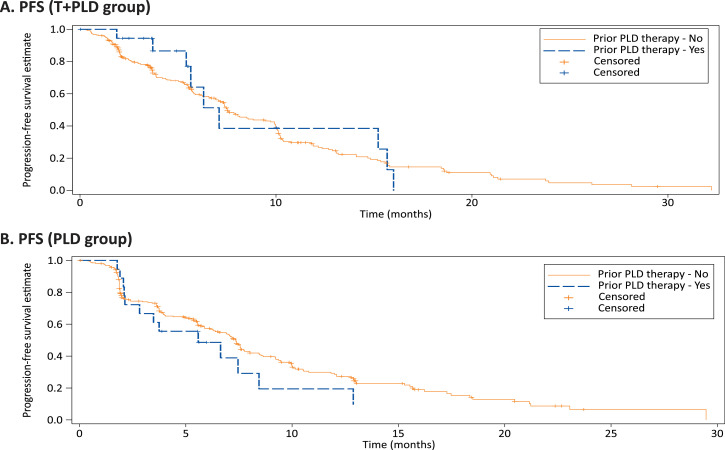
Kaplan–Meier estimates for progression free survival by prior PLD use in T+PLD (A) and PLD alone (B).

In a global phase 3 registration study, we performed a pre-stratified exploratory analysis to evaluate the impact of prior pegylated liposomal doxorubicin (PLD) on response rates and survival rates. In the protocol-specified un-stratified groups, the objective response rate (ORR) was higher in trabectedin+PLD (T+PLD) (46.0%) vs PLD group (35.9%) (odds ratio [OR]: 1.52; 95% CI: 1.07–2.16, *P* = 0.014). However, albeit limited patient numbers, prior PLD therapy did not influence overall survival (OS) or progression-free survival (PFS) between treatment arms (Fig. 1, 2). For T+PLD versus PLD alone, the ORR (OR: 2.06; 95% CI:0.48–9.07; *p* = 0.341), PFS (hazards ratio [HR]: 0.63; 95% CI: 0.26–1.48; *p* = 0.281), and OS (HR: 0.93; 95% CI: 0.33–2.60; *p* = 0.894) was similar. Furthermore, prior PLD use did not appear to influence ORR, PFS, or OS within each treatment group (Table 1). Combination T+PLD, as expected, elicited greater grade 3/4 treatment-emergent adverse events (TEAEs) than PLD alone, prior PLD therapy did not appear to impact the incidence of grade 3/4 TEAEs within each treatment arm, except for thrombocytopenia for T+PLD (Table 2). Use of PLD did not increase the incidences of any PLD-associated toxicities that generally occur after repeated treatment course of PLD, including palmar-plantar erythrodysesthesia, cardiac toxicities, and mucositis.

**Table 1:** Response rates and survival rates: randomized patients with prior PLD therapy.

**Efficacy**	**T+ PLD [*****n***** = 289]**			**PLD monotherapy [*****n***** = 287]**		
	**Prior PLD**			**Prior PLD**		
	Yes (*n* = 19, 6.6%)	No (*n* = 270, 93.4%)	HR (95% CI)	Yes (*n* = 20, 7%)	No (*n* = 267, 93%)	HR (95% CI)
ORR (%)	52.6	45.6	1.328 (0.468–3.819)	35	36	0.959 (0.313 - 2.692)
PFS (months)	7.1	7.5	0.853 (0.435–1.671)	5.6	7.4	1.212 (0.688–2.135)
OS (months)	34.2	22.1	0.844 (0.409–1.740)	28.9	20.9	0.713 (0.349–1.458)

CI, confidence interval; HR, hazard ratio; ORR, objective response rate; OS, overall survival; PFS, progression-free survival; PLD, pegylated liposomal doxorubicin; T, trabectedin.

**Table 2:** Safety of T+PLD vs PLD by prior PLD therapy use (safety analysis set).

**Safety**	**T + PLD [*****n***** = 289]**		**PLD monotherapy [*****n***** = 287]**	
	**Prior PLD**		**Prior PLD**	
	Yes (*n* = 19, 6.6%)	No (*n* = 267, 92.4%)	Yes (*n* = 20, 7%)	No (*n* = 262, 91.3%)
Grade 3/4 TEAEs, n (%)	18 (94.7)	225 (84.3)	14 (70)	166 (63.4)
Gastrointestinal	5 (26.3)	50 (18.7)	5 (25)	50 (19.1)
Nausea	3 (15.8)	18 (6.7)	1 (5)	3 (1.1)
Vomiting	3 (15.8)	15 (5.6)	1 (5)	4 (1.5)
Diarrhea	2 (10.5)	3 (1.1)	0	0
Hematologic	10 (52.6)	152 (56.9)	3 (15)	75 (28.6)
Anemia	4 (21.1)	57 (21.3)	1 (5)	19 (7.3)
Febrile neutropenia	2 (10.5)	20 (7.5)	1 (5)	2 (0.8)
Neutropenia	7 (36.8)	117 (43.8)	1 (5)	58 (22.1)
Leukopenia	3 (15.8)	38 (14.2)	0	20 (7.6)
Thrombocytopenia	4 (21.1)	39 (14.6)	0	3 (1.1)
Skin				
PPE	0	10 (3.7)	2 (10)	31 (11.8)
Cardiac		3 (1.1)	1 (5)	1 (0.4)
EF decreased	0	0	1 (5)	0
Atrial fibrillation		1 (0.4)		1 (0.4)
CHF		1 (0.4)		0

CHF, congestive heart failure; EF, ejection fraction; ORR, objective response rate; OS, overall survival; PFS, progression-free survival; PLD, pegylated liposomal doxorubicin; PPE, palmar-plantar erythrodysaesthesia; T, trabectedin; TEAE, treatment-emergent adverse event.

## 2 Experimental design, materials, and methods

Women with advanced-relapsed ROC having responded to 2 lines of platinum-based therapy were enrolled. Patients were randomly assigned 1:1 to combined trabectedin and PLD [trabectedin: 1.1 mg/m^2^, PLD: 30 mg/m^2^, IV, every 3 weeks] or PLD [PLD 50  mg/m^2^, IV, every 4 weeks]. The primary endpoint was OS. Secondary endpoints included PFS and ORR. Stratification was based on prior PLD use (yes or no). ClinicalTrials.gov #: NCT01846611.

## Conflict of Interest

Bradley J. Monk reports personal fees from Abbvie, personal fees from Advaxis, personal fees from Agenus, personal fees from Amgen, personal fees from AstraZeneca, personal fees from ChemoCare, personal fees from ChemoID, personal fees from Clovis, personal fees from Conjupro, personal fees from Easai, personal fees from Geistlich, personal fees from Genmab, personal fees from ImmunoGen, personal fees from Immunomedics, personal fees from Incyte, personal fees from Janssen (Johnson/Johnson), personal fees from Mateon (formally Oxigene), personal fees from Merck, personal fees from Myriad, personal fees from Nucana, personal fees from Oncomed, personal fees from Oncoquest, personal fees from Oncosec, personal fees from Perthera, personal fees from Pfizer, personal fees from Precison Oncology, personal fees from Puma, personal fees from Roche/Genentech, personal fees from Samumed, personal fees from Takeda, personal fees from Tesaro, personal fees from VBL, outside the submitted work. Thomas J, Herzog reports personal fees from J & J, personal fees from Clovis, personal fees from AstraZeneca, personal fees from Tesaro, personal fees from Roche, personal fees from Caris, outside the submitted work. Robert L. Coleman reports grants from NIH, grants from Gateway Foundation, grants from VFounation, during the conduct of the study; grants and personal fees fromAstraZeneca, grants fromMerck, personal fees from Tesaro, personal fees from Medivation, grants and personal fees from Clovis, personal fees from Gamamab, grants and personal fees from Genmab, grants and personal fees from Roche/Genentech, grants and personal fees from Janssen, personal fees from Agenus, personal fees from Regeneron, personal fees from OncoQuest, outside the submitted work. George Wang, Spyros Triantos, Scott Maul, Roland Knoblauch, Tracy McGowan, and Waleed Shalaby are employees of Johnson & Johnson and hold stocks.

## Appendix Supplementary material

Image, application 1
