# Supplementary material for: Data on prior pegylated liposomal doxorubicin (PLD) treatment in recurrent ovarian cancer: Post-hoc data analysis from the phase 3 randomized, open-label study comparing trabectedin and PLD versus PLD alone in patients with recurrent ovarian cancer
Source: Data Brief. 2020 Mar 20;30:105465. doi: 10.1016/j.dib.2020.105465 (PMC7178483; doi:10.1016/j.dib.2020.105465)
Supplement: Supplementary file 1 [file mmc1.pdf]

Janssen Research & Development*

Clinical Protocol

A Randomized, Open-Label Study Comparing the Combination of YONDELIS® and DOXIL®/CAELYX® with DOXIL®/CAELYX® Monotherapy for the Treatment of Advanced-Relapsed Epithelial Ovarian, Primary Peritoneal, or Fallopian Tube Cancer

Protocol ET743-OVC-3006; Phase 3

AMENDMENT 6

R279741 (trabectedin)

* YONDELIS, ET-743 (trabectedin), is being co-developed by Janssen Research & Development, LLC pursuant to a licensing arrangement with Pharma Mar, S.A. Janssen Research & Development is a global organization that operates through different legal entities in various countries. Therefore, the legal entity acting as the sponsor for Janssen Research & Development studies may vary, such as, but not limited to Janssen Biotech, Inc.; Janssen Products, LP; Janssen Biologics, BV; Janssen-Cilag International NV; Janssen, Inc; Janssen Infectious Diseases BVBA; Janssen Sciences Ireland UC; or Janssen Research & Development, LLC. The term "sponsor" is used throughout the protocol to represent these various legal entities; the sponsor is identified on the Contact Information page that accompanies the protocol.

This study will be conducted under US Food & Drug Administration IND regulations (21 CFR Part 312).

EudraCT NUMBER: 2012-004808-34

Status: Approved
Date: 9 January 2018
Prepared by: Janssen Research & Development, LLC
Document No: EDMS-ERI-41719962; 8.0

GCP Compliance: This study will be conducted in compliance with Good Clinical Practice, and applicable regulatory requirements.

Confidentiality Statement

The information in this document contains trade secrets and commercial information that are privileged or confidential and may not be disclosed unless such disclosure is required by applicable law or regulations. In any event, persons to whom the information is disclosed must be informed that the information is *privileged* or *confidential* and may not be further disclosed by them. These restrictions on disclosure will apply equally to *all* future information supplied to you that is indicated as privileged or confidential.

TABLE OF CONTENTS

TABLE OF CONTENTS 2

LIST OF ATTACHMENTS 4

LIST OF IN-TEXT TABLES AND FIGURES 5

PROTOCOL AMENDMENTS 6

SYNOPSIS 22

TIME AND EVENTS SCHEDULE (No Longer Applicable After Amendment 6) - TREATMENT ARM A (30 MG/M^2 DOXIL ADMINISTERED AS AN IV INFUSION OVER APPROXIMATELY 90 MINUTES FOLLOWED BY 1.1 MG/M^2 TRABECTEDIN ADMINISTERED AS AN IV INFUSION OVER APPROXIMATELY 3 HOURS EVERY 3 WEEKS) 27

TIME AND EVENTS SCHEDULE (No Longer Applicable After Amendment 6) - TREATMENT ARM B (50 MG/M^2 DOXIL ADMINISTERED AS AN IV INFUSION OVER APPROXIMATELY 90 MINUTES EVERY 4 WEEKS) 31

TIME AND EVENTS SCHEDULE – AMENDMENT 6 35

ABBREVIATIONS 36

1. INTRODUCTION 38
1.1. Trabectedin 39
1.1.1. Human Pharmacokinetics of Trabectedin 39
1.1.2. Metabolism of Trabectedin 39
1.2. Combination Therapy - Trabectedin + DOXIL 40
1.2.1. Pivotal Efficacy Study 40
1.2.2. Pivotal Safety Study 42
1.3. Comparator DOXIL 43
1.4. Overall Rationale for the Study 43

2. OBJECTIVES AND HYPOTHESIS 44
2.1. Objectives 44
2.2. Hypothesis 45

3. STUDY DESIGN AND RATIONALE 45
3.1. Overview of Study Design 45
3.2. Study Design Rationale 49

4. SUBJECT POPULATION 50
4.1. Inclusion Criteria 50
4.2. Exclusion Criteria 52
4.3. Prohibitions and Restrictions 54

5. TREATMENT ALLOCATION AND BLINDING 54

6. DOSAGE AND ADMINISTRATION 55
6.1. Criteria for Continuation of Treatment 56
6.2. Dose Modification 57
6.2.1. Dose Reductions Due to Hematologic Toxicity 58
6.2.2. Dose Reductions Due to Nonhematologic Toxicity 58

7. TREATMENT COMPLIANCE 59

8. PRESTUDY AND CONCOMITANT THERAPY 60

9. STUDY EVALUATIONS 61
9.1. Study Procedures 61
9.1.1. Overview 61
9.1.2. Screening Phase 62
9.1.3. Open-Label Treatment Phase 63
9.1.3.1. Clinical Laboratory Tests 64
9.1.3.2. Cardiovascular Safety 64
9.1.3.3. Physical Examination 65
9.1.3.4. Tumor Assessment 65
9.1.3.5. Patient Reported Outcomes Assessments 66
9.1.3.6. Other Assessments 66
9.1.4. Posttreatment Phase (Follow-Up) 66
9.2. Efficacy 67
9.2.1. Evaluations 67
9.2.2. Endpoints 68
9.3. Pharmacokinetics 68
9.3.1. Evaluations 68
9.3.2. Analytical Procedures 69
9.3.3. Pharmacokinetic Parameters 69
9.4. Pharmacogenomic Evaluations 69
9.4.1. Mandatory BRCA 1 and BRCA 2 Mutation Analysis 69
9.4.2. Optional Pharmacogenomic Analyses 69
9.5. Safety Evaluations 70
9.6. Sample Collection and Handling 72

10. SUBJECT COMPLETION/WITHDRAWAL 72
10.1. Completion 72
10.2. Discontinuation of Treatment 73
10.3. Withdrawal From the Study 73

11. STATISTICAL METHODS 74
11.1. Subject Information 74
11.2. Sample Size Determination 74
11.3. Efficacy Analyses 75
11.4. Pharmacokinetic Analyses 76
11.5. Patient Reported Outcomes 77
11.6. Pharmacogenomic Analyses 77
11.7. Safety Analyses 77
11.8. Interim Analysis 78
11.9. Independent Data Monitoring Committee 78

12. ADVERSE EVENT REPORTING 79
12.1. Definitions 79
12.1.1. Adverse Event Definitions and Classifications 79
12.1.2. Attribution Definitions 80
12.2. Special Reporting Situations 81
12.3. Procedures 81
12.3.1. All Adverse Events 81
12.3.2. Serious Adverse Events 82
12.3.3. Pregnancy 83
12.4. Contacting Sponsor Regarding Safety 84

13. PRODUCT QUALITY COMPLAINT HANDLING 84
13.1. Procedures 84
13.2. Contacting Sponsor Regarding Product Quality 84

14. STUDY DRUG INFORMATION 84
14.1. Physical Description of Study Drug(s) 84
14.2. Packaging 85
14.3. Labeling 85
14.4. Preparation, Handling, and Storage 85
14.5. Drug Accountability 86

15. STUDY-SPECIFIC MATERIALS 87

16. ETHICAL ASPECTS 87
16.1. Study-Specific Design Considerations 87
16.2. Regulatory Ethics Compliance 89
16.2.1. Investigator Responsibilities 89
16.2.2. Independent Ethics Committee or Institutional Review Board 89
16.2.3. Informed Consent 91
16.2.4. Privacy of Personal Data 92
16.2.5. Long-Term Storage of Samples for Future Research 92
16.2.6. Country Selection 93

17. ADMINISTRATIVE REQUIREMENTS 93
17.1. Protocol Amendments 93
17.2. Regulatory Documentation 93
17.2.1. Regulatory Approval/Notification 93
17.2.2. Required Prestudy Documentation 94
17.3. Subject Identification, Enrollment, and Screening Logs 94
17.4. Source Documentation 95
17.5. Case Report Form Completion 95
17.6. Data Quality Assurance/Quality Control 96
17.7. Record Retention 96
17.8. Monitoring 97
17.9. Study Completion/Termination 97
17.9.1. Study Completion 97
17.9.2. Study Termination 97
17.10. On-Site Audits 98
17.11. Use of Information and Publication 98

REFERENCES 100

INVESTIGATOR AGREEMENT 118

LIST OF ATTACHMENTS

Attachment 1: Evaluation of Response According to CA-125 (GCIG Criteria) 102
Attachment 2: RECIST Guidelines 103
Attachment 3: EORTC QLQ – OV28 110
Attachment 4: EQ–5D-5L Health Questionnaire 112
Attachment 5: New York Heart Association Classification 115
Attachment 6: Cockcroft-Gault Method 116
Attachment 7: Study Procedures as Outlined in Amendment 6 117

LIST OF IN-TEXT TABLES AND FIGURES

TABLES

Table 1: Criteria for Continuation of Treatment 57
Table 2: Dose Level Reductions – Trabectedin + DOXIL Group 58
Table 3: Dose Level Reductions – DOXIL Group 58
Table 4: Dose Reductions for Hematologic Toxicity 58
Table 5: Dose Reductions for Nonhematologic Toxicity 59
Table 6: Statistical Operating Characteristics 78

FIGURES

Figure 1: Kaplan-Meier Plot of Progression-Free Survival 40
Figure 2: Kaplan-Meier Plot of Overall Survival 41
Figure 3: Overall Survival by Platinum-Free Interval 42
Figure 4: Study Design 48

PROTOCOL AMENDMENTS

Protocol Version	Issue Date
Original Protocol	19 December 2012
Amendment INT-1	25 March 2013
Amendment INT-2	29 August 2013
Amendment INT-3	26 August 2015
Amendment INT-4	17 December 2015
Amendment INT-5	18 March 2016
Amendment 6	9 January 2018

Amendments are listed beginning with the most recent amendment.

Amendment 6 (9 January 2018)

This amendment is considered to be substantial based on the criteria set forth in Article 10(a) of Directive 2001/20/EC of the European Parliament and the Council of the European Union.

The overall reason for the amendment: The sponsor's decision to amend the study protocol was based on the Independent Data Monitoring Committee (IDMC) recommendation to discontinue the study based on the results of a futility analysis of overall survival (OS), in which the prespecified futility threshold was crossed. In addition, the amendment will allow study subjects deriving clinical benefit to continue on single-agent DOXIL® per local standard of care.

Applicable Section(s)	Description of Change(s)
**Rationale:** Study conduct was changed due to the IDMC recommendation to discontinue the study.	
Synopsis; Section 3.1, Overview of Study Design; Section 6, Dosage and Administration; Section 9, Study Evaluations; Section 9.1.3, Open-Label Treatment Phase; Section 9.1.4, Posttreatment Phase (Follow-up); Section 9.5, Safety Evaluations; Section 10.1, Completion; Section 10.2, Discontinuation of Treatment; Section 11.9, Independent Data Monitoring Committee; Section 14, Study Drug Information; Section 16.1, Study-Specific Design Considerations; Attachment 7	Text was added in full or in part to reflect the following: On 26 June 2017, the IDMC requested a futility analysis of OS to be performed at the time of the next meeting 6 months later. Following the review of the study data by the IDMC on 15 December 2017, the HR for OS was 0.962, crossing the previously agreed upon threshold for futility of 0.93. In view of this result and the observed and expected higher toxicity in Arm A as compared with Arm B, the IDMC recommended discontinuing the study. Therefore, as of Amendment 6, no new subjects will be randomized to study treatment, and treatment with trabectedin should be immediately discontinued for subjects assigned to Arm A (trabectedin+DOXIL). All study subjects (Arm A or Arm B) currently on study who, in the opinion of the investigator, are deriving clinical benefit may continue treatment with single-agent DOXIL as per the local standard of care. Treatment for these subjects may continue as long as the subjects comply with protocol-specified prohibitions and restrictions for treatment and as long as they experience clinical benefit in the opinion of the investigator. DOXIL will be provided by JR&D as needed until post-trial access to DOXIL is available or until disease progression, whichever occurs first.<br>Accordingly, the end of study data collection is defined as when all subjects on study treatment have completed treatment termination visit assessments as specified in the Time and Events Schedule for Amendment 6 or by 18 January 2018, whichever occurs first. For subjects continuing treatment with single-agent DOXIL, as per the local standard of care, only serious adverse events should be reported to JR&D.<br>All subjects still on study treatment should be informed of the reasons for the discontinuation of the study, and the potential risks and benefits of continued treatment with single-agent DOXIL should be discussed.

Applicable Section(s)	Description of Change(s)
**Rationale:** To indicate that subjects in screening will not be randomized to study treatment and that no new subjects will be screened.	
Section 3.2, Study Design Rationale; Section 4, Subject Population; Section 5, Treatment Allocation and Blinding; Attachment 7	Text was added in full or in part to reflect the following: Following Amendment 6, subjects will not be randomized to study treatment, and no new subjects will be screened. Subjects currently in screening are be eligible to receive treatment with single-agent DOXIL, if the investigator confirms that such treatment would be a clinically appropriate therapeutic option.
**Rationale:** To indicate that subjects in screening who are eligible to receive treatment with single-agent DOXIL should meet study inclusion and exclusion criteria as specified in Amendment 6.	
Section 4, Subject Population; Section 4.1, Inclusion Criteria; Section 4.2, Exclusion Criteria, Attachment 7	Text was added to indicate that: Following Amendment 6, in those instances where the investigator confirms that single-agent treatment with DOXIL would be a clinically appropriate therapeutic option for subjects currently in screening, those subjects in screening would need to meet study inclusion and exclusion criteria as specified in Sections 4.1 and 4.2.<br>In addition, inclusion criteria #7 and #9 and exclusion criteria #3 and #5 no longer apply for these subjects per Amendment 6 for the following reasons: efficacy will not be monitored for subjects receiving single-agent DOXIL after Amendment 6, IV administration of dexamethasone is only applicable for subjects receiving trabectedin and trabectedin treatment ends with Amendment 6, central venous catheter access is only needed for trabectedin administration (no longer applicable), hypersensitivity to trabectedin and it's excipients is no longer applicable..
**Rationale:** Text was added regarding the reporting of serious adverse events.	
Synopsis; Section 3.1, Overall Study Design; Section 9.5, Safety Evaluations; Section 10.1, Completion; Section 12, Adverse Event Reporting; Section 12.3.2, Serious Adverse Events; Attachment 7	Text was added to indicate that: Following the IDMC recommendation to stop the study (clinical cutoff date of 18 January 2018), only serious adverse events for subjects continuing to receive single-agent DOXIL should be reported to JR&D. Text was also added, where appropriate, to indicate when the reporting of serious adverse events for subjects receiving single-agent DOXIL would end.
**Rationale**: To provide a Time and Events Schedule for study procedures following Amendment 6.	
Time and Events Schedule – Amendment 6	All study procedures at the time of Amendment 6 are summarized in Time and Events Schedule. A summary of study conduct in text format is provided in Attachment 7.
**Rationale:** To indicate that concomitant therapies and monitoring of overall survival will end at the clinical cutoff date (18 January 2018).	

Applicable Section(s)	Description of Change(s)
Synopsis; Section 3.1, Overview of Study Design; Section 8, Prestudy and Concomitant Therapy; Section 9.1.4, Posttreatment Phase (Follow-up); Section 9.2.1, Evaluations; Section 11.3, Efficacy Analyses	Text was updated to indicated that the collection of survival status will continue until at least 514 deaths have been observed or until the clinical data cutoff date. In addition, concomitant therapies must be recorded throughout the study beginning from the time the ICF is signed to 30 days after the last dose of study drug or until 18 January 2018, whichever occurs first.
**Rationale:** To provide data collection guidance regarding adverse event reporting and special reporting situations.	
Section 12.2, Special Reporting Situations; Section 12.3.1, All Adverse Events	Updated text was provided to indicate that adverse events and special reporting situations, will be reported up to the clinical cutoff date of 18 January 2018.
**Rationale:** To indicate that follow-up data collection will end with Amendment 6.	
Synopsis; Section 9.1.4, Posttreatment Phase (Follow-up); Section 12.3.1, All Adverse Events	Text was added to indicate that follow-up data will no longer be collected. Text regarding follow-up for significant declines in LVEF was deleted.
**Rationale:** Minor errors were noted	
Throughout the protocol	Minor grammatical, formatting, or spelling changes were made.

<u>Amendment INT-5</u> (18 March 2016)

This amendment is considered to be nonsubstantial based on the criteria set forth in Article 10(a) of Directive 2001/20/EC of the European Parliament and the Council of the European Union, in that it does not significantly impact the safety or physical/mental integrity of subjects, nor the scientific value of the study.

The overall reason for the amendment: The overall reason for the amendment is to add text regarding the monitoring and recording of significant declines in left ventricular ejection fraction.

Applicable Section(s)	Description of Change(s)
**Rationale:** To clarify the timing of LVEF assessments for subjects experiencing a significant decline in LVEF that has not resolved by the end of treatment.	
Synopsis, Safety Evaluations; Time and Events Schedules; Section 9.1.4, Posttreatment Phase (Follow-up); Section 12.3.1, All Adverse Events	The following text was added: Subjects experiencing significant LVEF decline (defined as absolute decrease ≥15%, or less than LLN and absolute decrease ≥5%) not recovered to less than Grade 2 (or baseline) by the end of treatment should have follow up assessments of LVEF every 2 months until recovered or up to 6 months after discontinuation of study treatment (whichever occurs first).

Applicable Section(s)	Description of Change(s)
**Rationale:** To clarify the dose reduction guidelines to be followed if DOXIL is discontinued due to cardiac toxicity.	
Section 6.2.2, Dose Reduction Due to Nonhematologic Toxicity	The following text was added: If DOXIL is discontinued due to cardiac toxicity, then refer to Table 5 for treatment guidelines for trabectedin monotherapy.
**Rationale:** To clarify the reporting of significant decreases in LVEF.	
Section 6.2.2, Dose Reduction Due to Nonhematologic Toxicity	The following text was added: Any significant decrease in LVEF (defined as absolute decrease ≥15%, or less than LLN and absolute decrease ≥5%) should be captured as an adverse event regardless of assessed clinical significance or action taken. The supplemental cardiac adverse events form should also be completed for these adverse events.
**Rationale:** To clarify that subsequent LVEF assessments may be required during the follow-up period.	
Section 9.5, Safety Evaluations	The following text was added: Subsequent LVEF assessments may be indicated during the follow-up period if significant LVEF decline is noted (Section 9.1.4) at the time of end of treatment.
**Rationale:** To clarify the reporting of LVEF assessments conducted during the follow-up period.	
Section 12.3.1, All Adverse Events	The following text was added: These assessments of LVEF are to be reported in the eCRF.
**Rationale:** To clarify the reporting of data for subjects who discontinue study drug due to cardiotoxicity or significant decreases in LVEF.	
Section 12.3.1, All Adverse Events	The following text was added: For study subjects who discontinue a study drug for cardiotoxicity or who have a significant decrease in LVEF, all cardiac adverse events and relevant concomitant therapies must be recorded until the events resolve or for 6 months after the last dose of study drug (whichever occurs first). Any significant decrease in LVEF should be captured as an adverse event regardless of assessed clinical significance or action taken. The supplemental cardiac adverse events form should also be completed for these adverse events.
**Rationale:** To clarify the no change in dose is required for the first occurrence of an LVEF decline ≥10% and <LLN or clinical evidence of cardiomyopathy.	
Table 5	Text was added indicating no change in dose is required for the first occurrence of an LVEF decline ≥10% and <LLN or clinical evidence of cardiomyopathy.
**Rationale:** Minor errors were noted	
Throughout the protocol	Minor grammatical, formatting, or spelling changes were made.

Amendment INT-4 (17 December 2015)

This amendment is considered to be nonsubstantial based on the criteria set forth in Article 10(a) of Directive 2001/20/EC of the European Parliament and the Council of the European Union, in that it does not significantly impact the safety or physical/mental integrity of subjects, nor the scientific value of the study.

The overall reason for the amendment: The overall reason for the amendment is to add additional left ventricular ejection fraction evaluations.

Applicable Section(s)	Description of Change(s)
**Rationale:** To add the United States regulatory approval of trabectedin.	
Synopsis; Section 1, Introduction	The approved United States indication was added.
**Rationale:** To include 2-dimensional echocardiograms as an evaluation choice regardless of the availability of multigated acquisition scans.	
Synopsis, Overview of Study Design and Safety Evaluations; Time and Events Schedule; Section 3.1 Overview of Study Design; Section 9.1.2, Screening Phase; Section 9.1.3.2, Cardiovascular Safety; Section 9.5, Safety Evaluations; Section 11.7, Safety Analyses	The protocol was changed to reflect that either MUGA scans or 2D-ECHOs can be used for LVEF assessments; with the understanding that the same imaging technique should be used consistently throughout the study.
**Rationale:** To include the final cardiac analysis and report.	
Section 3.1, Overview of Study Design; Section 11.7, Safety Analyses	Text was added to indicate that a-final cardiac analysis will be conducted and a report will be provided.
**Rationale:** To add additional LVEF assessments.	
Synopsis, Safety Evaluations; Time and Events Schedule; Section 9.1.3.2, Cardiovascular Safety; Section 9.5, Safety Evaluations	Additional assessments of LVEF were added to reflect the following complete LVEF assessment schedule:<br>Left ventricular ejection fraction assessments (MUGA scans or 2D-ECHOs) will be performed for all subjects at screening (baseline), after an approximately 100 mg/m^2 cumulative dose of planned anthracycline treatment with DOXIL (ie, after Cycle 3 for treatment Arm A and after Cycle 2 for treatment Arm B) and as part of end-of-treatment assessments (ie, end-of-treatment termination visit). In addition, those subjects who receive a total cumulative dose of anthracycline exceeding 300 mg/m^2 (including previous and on-study treatments) or have a clinically significant history of cardiomyopathy should have follow-up LVEF assessments after every 2 cycles of therapy, or as clinically warranted. The same methodology (MUGA scans or 2D-ECHOs) should be used to assess scheduled LVEF assessments throughout the study. Additional LVEF assessments performed during the study should be reported as unscheduled LVEF assessments.
**Rationale**: To specify that waivers to inclusion and exclusion criteria are not allowed.	
Section 4, Subject Population	Text was added to indicate that any questions regarding the inclusion/exclusion criteria should be resolved before enrolling a subject, and to indicate that waivers to the inclusion/exclusion criteria are not allowed.
**Rationale:** To reiterate that the use of prophylactic granulocyte-colony stimulating factor is strongly recommended.	
Section 6, Dosage and Administration; Section 6.2.1, Dose Reductions Due to Hematologic Toxicity	Text was added noting that the use of prophylactic granulocyte-colony stimulating factor is strongly recommended for subjects randomized to treatment Arm A.

Applicable Section(s)	Description of Change(s)
**Rationale:** To add the LVEF assessment to the list of criteria to be met for continuation of treatment (as noted in Table 1) and to update the corresponding text regarding this criterion.	
Section 6.1, Criteria for Continuation of Treatment	Table 1 was updated to reflect that the most recent LVEF assessment must be ≥ the lower limit of normal [LLN] for treatment to continue. In addition, the following corresponding text regarding continuation of treatment based on the LVEF assessment was updated: Subjects must discontinue DOXIL for clinical evidence of cardiac toxicity or if the LVEF is compromised (ie, any absolute decrease in LVEF ≥10% and an LVEF value below the institutional lower limit of normal [LLN]). However, dosing with trabectedin should continue if the criteria for continuation of treatment are met (Table 1) at the same dose. For those subjects on treatment Arm A who continue with trabectedin as a monotherapy after a cumulative anthracycline dose of 300 mg/m^2 has been exceeded or due to cardiotoxicity, regular monitoring of LVEF every 2 cycles of treatment should continue, regardless of the cumulative dose of anthracycline. All subjects in Arm A should undergo LVEF assessments after Cycle 3 of study treatment, even if DOXIL has been discontinued.
**Rationale:** To add parameters for dose reductions in cases of LVEF decline or cardiomyopathy.	
Section 6.2.2, Dose Reductions Due to Nonhematologic Toxicity	Table 5 was updated to reflect dose reductions at first and subsequent occurrences of LVEF decline ≥10% and <LLN or clinical evidence of cardiomyopathy.
**Rationale:** To add text indicating the follow-up procedures to potential cases of cardiac toxicity and the documentation required.	
Section 9.5, Safety Evaluations	The following text was added:<br>In selected cases of cardiac adverse events, investigators may be asked to complete a supplemental CRF section in order to provide more detailed information relating to the event. Any additional LVEF assessments should be reported as unscheduled assessments.
**Rationale:** Text added for clarification regarding local requirements.	
Section 16.2.2, Independent Ethics Committee or Institutional Review Board	Text was changed from: "This study will be undertaken only after the IEC/IRB has given full approval of the final protocol, amendments (if any, excluding the ones that are purely administrative, with no consequences for subjects, data, or study conduct)…" to "This study will be undertaken only after the IEC/IRB has given full approval of the final protocol, amendments (if any, excluding the ones that are purely administrative, with no consequences for subjects, data, or study conduct, unless required locally)…".
**Rationale:** For clarity, minor wording changes were made regarding the contact information page.	
Section 17.1, Protocol Amendments	Minor wording changes were made to clarify that the Contact Information page will be provided as a separate document.
**Rationale:** For clarity, minor wording changes were made regarding source documentation.	
Section 17.4, Source Documentation	The following text was moved from the beginning of the 3rd paragraph to the beginning of the first paragraph: "At a minimum, source documents consistent in the type and level of detail with that commonly recorded at the study site as a basis for standard medical care."
**Rationale:** To add text regarding the verification of inclusion/exclusion criteria.	
Section 17.4, Source Documentation	The following text was added: "Inclusion and exclusion criteria not requiring documented medical history must be verified at a minimum by subject interview or other protocol required assessments (eg, physical examination, laboratory assessment) and documented in the source documents."

Applicable Section(s)	Description of Change(s)
**Rationale:** To ensure the recording and accuracy of data in the CRF.	
Section 17.5, Case Report Form Completion	The following text was added:<br>"All CRF entries, corrections, and alterations must be made by the investigator or authorized study-site personnel. The investigator must verify that all data entries in the CRF are accurate and correct."
**Rationale:** To update the method for correcting the CRF.	
Section 17.5, Case Report Form Completion	The method for correcting the CRF was updated to the following: "If corrections to a CRF are needed after the initial entry into the CRF, this can be done in either of the following ways:<br>• Investigator and study-site personnel can make corrections in the eDC tool at their own initiative or as a response to an auto query (generated by the eDC tool).<br>• Sponsor or sponsor delegate can generate a query for resolution by the investigator and study-site personnel."
**Rationale:** For clarity, minor wording changes were made to ensure: 1) the sponsor review of the CRFs after transmission to the sponsor and 2) verification for accuracy and consistency with data sources after the upload of the data into the study database.	
Section 17.6, Data Quality Assurance/Quality Control	The wording was revised to: "The sponsor will review CRFs for accuracy and completeness during on-site monitoring visits and after transmission to the sponsor; any discrepancies will be resolved with the investigator or designee, as appropriate. After the upload of the data into the study database, they will be verified for accuracy and consistency with the data sources."
**Rationale:** For clarity, additional examples of source documents were specified.	
Section 17.8, Monitoring	The following examples were provided as source documents: hospital/clinic/physician's office medical records.
**Rationale:** Minor editorial changes were made.	
Throughout the protocol	Minor grammatical, formatting, or spelling changes were made.<br>The terms multiple gated acquisition scan and multi gated acquisition scan were corrected to multigated acquisition scan. The MedDRA version number was deleted.

Amendment INT-3 (26 August 2015)

This amendment is considered to be nonsubstantial based on the criteria set forth in Article 10(a) of Directive 2001/20/EC of the European Parliament and the Council of the European Union, in that it does not significantly impact the safety or physical/mental integrity of subjects, nor the scientific value of the study.

The overall reason for the amendment: The overall reason for the amendment is to add to and revise study inclusion and exclusion criteria to allow greater flexibility in demonstrating eligibility based on response to previous therapy.

Applicable Section(s)	Description of Change(s)
**Rationale:** To clarify that study treatment will continue until 2 cycles after assessment of a complete response.	
Synopsis, Overview of Study Design; Section 3.1, Overview of Study Design	The following statement was changed from “Treatment will continue until the occurrence of disease progression or unacceptable treatment toxicity, or until 2 cycles beyond a confirmed complete response” to “Treatment will continue until the occurrence of disease progression or unacceptable treatment toxicity, or until 2 cycles after assessment of a complete response (CR).”
**Rationale:** To clarify that disease progression is to be radiographically confirmed.	
Synopsis, Overview of Study Design, Efficacy Evaluations/Endpoints; Time and Events Schedule footnotes; Section 3.1, Overview of Study Design, Figure 4; Section 9.1.3.1, Clinical Laboratory Tests; Section 9.1.3.4, Tumor Assessment; Section 9.1.4, Posttreatment Phase (Follow-up); Section 9.2.1, Evaluations	Specified that disease progression is to be confirmed radiographically.
**Rationale:** To clarify that documentation of all subsequent anticancer therapy, survival status, and safety evaluations are required for all subjects discontinuing study treatment and not just those subjects with disease progression at the time of treatment discontinuation.	
Synopsis, Overview of Study Design; Section 3.1, Overview of Study Design; Section 9.1.4, Posttreatment Phase (Follow-up)	The following statement was changed from “For subjects with disease progression at the time of treatment discontinuation, documentation of all subsequent anticancer therapy, survival status, and safety evaluations as outlined in the Time and Events Schedule will be required.” to “For subjects discontinuing study treatment, documentation of all subsequent anticancer therapy, survival status, and safety evaluations as outlined in the Time and Events Schedule will be required.” Related text/statements were also changed.
**Rationale:** Inclusion criterion #5 was changed to specify serum CA-125 levels.	
Synopsis, Subject Population; Section 4.1, Inclusion Criteria	Inclusion criterion #5 was changed to specify that a subject was to have received second-line treatment with a platinum-based regimen, with progression of disease after attaining a response. A response could have been determined by imaging or by serum CA-125 levels. Reference to the Gynecologic Cancer Intergroup outline was deleted.

Applicable Section(s)	Description of Change(s)
**Rationale:** For clarity, editorial changes were made to inclusion criterion #11 regarding subjects receiving recombinant erythropoietin therapy.	
Section 4.1, Inclusion Criteria	Inclusion criterion #11 was changed to reflect that subjects may be enrolled into the study while receiving recombinant erythropoietin provided that they have received recombinant erythropoietin for at least 4 weeks before the first dose of study drug.
**Rationale:** Inclusion criterion #18 was changed to define postmenopausal and surgical sterility, to indicate that male and female condoms should not be used together, and to note that if childbearing potential changes after the start of the study, 2 effective methods of birth control must be used.	
Section 4.1, Inclusion Criteria	Inclusion criterion #18 was revised to "Be postmenopausal (>45 years of age with amenorrhea for at least 2 years or any age with amenorrhea for at least 6 months and a serum follicle stimulating hormone level >40 IU/L or mIU/mL), surgically sterile (have had a hysterectomy or bilateral oophorectomy, bilateral salpingectomy, bilateral tubal occlusion ligation [which includes tubal ligation procedures as consistent with local regulations], or otherwise be incapable of pregnancy), abstinent (acceptability of this method is at the discretion of the investigator who will ensure and document that the subject understands the definition of "abstinence", and who will periodically remind and counsel the subject on this topic), or if heterosexually active, be practicing two effective methods of birth control (eg, prescription hormonal contraceptive, intrauterine device, double-barrier method [eg, condoms, occlusive cap (diaphragm or cervical/vault caps) with spermicidal foam, cream, gel, film, or suppository]), before enrollment, and must agree to continue to use the same two methods of contraception throughout the study and for 6 months thereafter. Note: a female condom and a male condom should not be used together as friction between the two can result in either product failing.<br>Note: If the childbearing potential changes after the start of the study (eg, woman who is not heterosexually active becomes active), then the woman must begin two effective methods of birth control, as described above".
**Rationale:** To include inclusion criterion #22.	
Section 4.1, Inclusion Criteria	Inclusion criterion #22 was added and states: Each subject "must agree not to donate eggs (ova, oocytes) for the purposes of assisted reproduction".
**Rationale:** Exclusion criterion #2 was changed to clarify the number and type of prior lines of therapy.	
Synopsis, Subject Population; Section 4.2 Exclusion Criteria	Exclusion criterion #2 was changed from: "A subject who had more than 2 prior lines of chemotherapy" to "A subject who had more than 2 prior lines of systemic therapy. Maintenance therapies and hormonal therapies are not considered additional lines of therapy." Related text/statements were also changed.
**Rationale:** Exclusion criterion #7 was revised to clearly state the time period between study treatment and other specified therapies.	
Section 4.2, Exclusion Criteria	Exclusion criterion #7 was changed from "A subject who is less than 3 weeks from radiation therapy, experimental therapy, hormonal therapy, prior chemotherapy, or biological therapy" to "A subject who would receive study treatment within 3 weeks from radiation therapy, experimental therapy, hormonal therapy, prior chemotherapy, or biological therapy; use an invasive investigational device; or is currently enrolled in an investigational study".

Applicable Section(s)	Description of Change(s)
**Rationale:** To change neoplastic disease to invasive malignancy in exclusion criterion #8.	
Section 4.2, Exclusion Criteria	Exclusion criterion #8 was changed from "A subject who has a history of another neoplastic disease (except non-metastatic basal cell carcinoma or squamous cell carcinoma of the skin or cervical carcinoma in situ adequately treated) unless in remission for ≥5 years" to "A subject who has a history of another invasive malignancy (except non-metastatic basal cell carcinoma or squamous cell carcinoma of the skin adequately treated) unless in remission for ≥5 years, or a non-invasive malignancy requiring ongoing therapy".
**Rationale:** To clarify the dosing administration for Treatment Arm A and Treatment Arm B and to add approximate durations of infusions.	
Synopsis, Dosage and Administration; Time and Events Schedule; Section 3.1, Overview of Study Design Figure 4; Section 6, Dosage and Administration	Changes were made to the dosing administration of DOXIL and trabectedin. The revised text reflects the following:<br>Treatment Arm A: DOXIL 30 mg/m^2 administered as an IV infusion over approximately 90 minutes (per package insert) followed by trabectedin 1.1 mg/m^2, administered as an IV infusion over approximately 3 h, via central venous access, every 3 weeks. If no infusion-related reaction is observed with DOXIL after the first infusion, DOXIL may be administered as a 60- to 90-minute infusion in subsequent cycles.<br><br>Treatment Arm B: DOXIL, 50 mg/m^2 administered as an IV infusion over approximately 90 minutes (per package insert) every 4 weeks. If no infusion-related reaction is observed with DOXIL after the first infusion, DOXIL may be administered as a 60- to 90-minute infusion in subsequent cycles.
**Rationale:** To add text indicating that investigators should review adverse events before each dose of study drug.	
Synopsis, Dosage and Administration	The terms "adverse events" were added to the following sentence and related text: "Investigators will review physical findings, adverse events, and the results of clinical laboratory tests before each dose of study drug and, if required, delay administration or reduce the dose according to the protocol-defined guidelines".
**Rationale:** To clarify the timing of the CA-125 evaluations.	
Synopsis, Efficacy Evaluation/Endpoints; Time and Events Schedule Footnotes; Section 9.1.3.1, Clinical Laboratory Tests; Section 9.2.1, Evaluations	The text regarding CA-125 evaluations now reflects that the evaluations should coincide (±5 days) with the scheduled tumor assessments. The ±5 day window for the assessment was added for clarity.
**Rationale:** To clarify the timing of the clinical laboratory evaluations.	
Synopsis, Safety Evaluations; Time and Events Schedule; Section 9.1.3.1, Clinical Laboratory Tests	The timing of the clinical laboratory evaluations was clarified and now reflect the following:<br>Clinical laboratory test results will be evaluated before dosing (ie, within 2 days prior to dosing) on Day 1 of each cycle; and<br><br>Blood samples will also be collected for clinical laboratory testing on +/- 2 days on Days 8 and 15 (Treatment Arm A) or Days 8, 15, and 22 (Treatment Arm B) of each cycle and at the end of treatment.

Applicable Section(s)	Description of Change(s)
**Rationale:** To add text regarding the capping of body surface area (BSA) utilized for dosing calculations.	
Time and Events Schedule Footnotes; Section 6, Dosage and Administration	The text regarding the capping of BSA utilized for dosing calculations was added. Example text: For those subjects with a body mass index (BMI) ≥30, the use of ideal body weight for dose calculation and capping of BSA utilized for dosing calculations will be left to the discretion of the investigator and to institutional guidelines.
**Rationale:** To clarify that all subjects who discontinue study treatment will be monitored for survival.	
Section 3.1, Overview of Study Design	The following text was changed from "Subjects who discontinue study treatment due to evidence of disease progression or unacceptable toxicity will be monitored for survival" to "All subjects who discontinue study treatment will be monitored for survival".
**Rationale:** To delete text regarding alopecia from Table 1.	
Section 6.1, Criteria for Continuation of Treatment, Table 1 Footnote	The term "alopecia" was deleted from footnote "b" in Table 1.
**Rationale:** To clarify the dexamethasone premedication is not required if trabectedin treatment is discontinued.	
Section 6.2, Dose Modification	The following text was added to the protocol: "If trabectedin is discontinued for a subject in Treatment Arm A, premedication with dexamethasone is no longer required".
**Rationale:** To clarify that investigators may reduce the dose of either or both study drugs for any toxicity and subjects who experience toxicities that require more than 2 dose reductions of the same drug must discontinue study treatment with that drug.	
Section 6.2, Dose Modification; Section 6.2.1, Dose Reductions Due to Hematologic Toxicity; 6.2.2, Dose Reductions Due to Nonhematologic Toxicity	Text was added to clarify that:<br>Investigators may also reduce the dose of either or both study drugs for any toxicity as clinically warranted, and<br><br>Only 2 dose reductions of each study drug will be allowed. Subjects who experience toxicities that require more than 2 dose reductions of the same drug must discontinue study treatment with that drug.
**Rationale:** To clarify the parameters for dose reductions for nonhematologic toxicity.	
Section 6.2.2, Dose Reductions Due to Nonhematologic Toxicity, Table 5	In Table 5, the footnotes were updated, direct bilirubin was changed to bilirubin, criteria for a dose reduction due to alkaline phosphatase were updated, and criteria for creatine phosphokinase were added.

Applicable Section(s)	Description of Change(s)
**Rationale:** To clarify the start of the screening phase and activities to be completed during this study phase.	
Section 9.1.2, Screening Phase	Text was revised to reflect the following: "The Screening Phase begins with the signing of the informed consent and should not last longer than 30 days. During the Screening Phase, the medical monitors will confirm subject eligibility based on previous therapy history, according to inclusion criteria #4 through #8 [Section 4.1, Inclusion Criteria]). Response to second-line platinum-based therapy will be confirmed either based on imaging demonstrating at least a 30% decline in tumor burden or on a 50% reduction of elevated baseline serum CA-125 levels (≥2 x ULN, measured within approximately 4 weeks prior to the first dose of second-line platinum-based therapy that is maintained after at least 28 days as modified from Gynecologic Cancer Intergroup [GCIG] criteria [Attachment1])."<br><br>In addition, the following screening procedures to be completed within 30 days before randomization were revised or added:<br>Submission of redacted source documents supporting subject eligibility relevant to inclusion criteria #4 through #8.<br><br>On or prior to Day 1 of Cycle 1, all subjects will be provided with a "wallet (study) card" and instructed to carry this card with them for the duration of the study.
**Rationale:** To clarify the timing of absolute neutrophil count (ANC) determination.	
Section 9.1.3.1, Clinical Laboratory Texts	Text was revised to reflect that: If a subject has an ANC of <500/μL, the ANC for this subject should be repeated at most 5 days later to document recovery to >500/μL.
**Rationale:** To clarify disease assessments should be performed at the treatment termination visit.	
Section 9.1.3.4, Tumor Assessment	Text was revised from: "disease assessments must be performed at the treatment termination visit…" to disease assessments should be performed at the treatment termination…".
**Rationale:** To clarify assessment required in case of clinical progression.	
Section 9.1.3.4, Tumor Assessment	Text was changed from: "In case of clinical or symptomatic progression, the same tumor assessments will be performed to document the progression. If progression is not confirmed at this unscheduled radiologic assessment, subsequent radiologic assessments should proceed according to the original Time and Events Schedule" to In case of clinical progression, radiologic assessments should proceed according to the original Time and Events Schedule, or as clinically indicated".
**Rationale:** To clarify that the treatment termination visit should occur 30 days (+3 days) after the last dose of study drug.	
Section 9.1.4, Posttreatment Phase (Follow-up)	Text was revised to state that the treatment termination visit should occur 30 days (+3 days) after the last dose of study drug.
**Rationale:** Text was added regarding CA-125 use.	
Section 9.2.1 Evaluations	The following text was added: "CA-125 shall not be used as a basis to assess disease response or progression during study treatment and should not guide treatment decisions."

Applicable Section(s)	Description of Change(s)
**Rationale:** To add/delete criteria for subject discontinuation from treatment.	
Section 10.2, Discontinuation of Treatment	The following was deleted from the list criteria for treatment discontinuation:<br>The investigator believes that for safety reasons (eg, adverse event) it is in the best interest of the subject to stop treatment.<br>The following were added to or revised in the list of criteria for treatment discontinuation:<br>The subject meets protocol prescribed criteria for drug discontinuation due to toxicity after appropriate dose reduction (Section 6.2).<br><br>The subject develops a concurrent medical condition (adverse event) that precludes further participation.<br><br>The subject completes 2 cycles of treatment after assessment of CR.
**Rationale:** To add text regarding the reporting of suspected unexpected serious adverse reactions.	
Section 12.3.1, All Adverse Events	The following text was added: "The sponsor will also report to the investigator (and the head of the investigational institute where required) all suspected unexpected serious adverse reactions (SUSARs). The investigator (or sponsor where required) must report SUSARs to the appropriate Independent Ethics Committee/Institutional Review Board (IEC/IRB) that approved the protocol unless otherwise required and documented by the IEC/IRB".
**Rationale:** To add to the list of abnormal pregnancy outcomes.	
Section 12.3.3, Pregnancy	The following were added to the list of abnormal pregnancy outcomes: fetal death and ectopic pregnancy.
**Rationale:** To add text regarding study monitoring.	
Section 17.8, Monitoring	The following text was added:<br>"The sponsor will use a combination of monitoring techniques (central, remote, or on-site monitoring) to monitor the study."<br><br>"In addition, to on-site monitoring visits, remote contact can occur. It is expected that during these remote contact, study-site personnel will be available to provide an update on the progress of the study at the site."<br><br>"Central monitoring will take place for data identified by the sponsor as requiring central review."
**Rationale:** Minor editorial changes were made throughout the protocol.	
Throughout the protocol	Minor editorial, grammatical, formatting, or spelling changes were made. Changes terminology were also made (eg, 48 h changed to 2 days; study medication changed to study drug)

Amendment INT-2 (29 August 2013)

This amendment is considered to be substantial based on the criteria set forth in Article 10(a) of Directive 2001/20/EC of the European Parliament and the Council of the European Union.

The overall reason for the amendment: The overall reasons for the amendment are to extend the use of contraceptives from 3 months to 6 months after the study, to increase the creatinine clearance rate from ≥40 mL/min/1.73 m^2 to ≥60 mL/min/1.73 m^2, and to add a prohibition regarding subjects receiving a yellow fever vaccine.

Applicable Section(s)	Description of Change(s)
**Rationale:** Inclusion criterion 11 was modified to increase the calculated glomerular filtration rate from ≥40 mL/min/1.73 m^2 to ≥60 mL/min/1.73 m^2.	
Section 4.1, Inclusion Criteria	The following statement was changed from "either a serum creatinine ≤1.5 mg/dL or a calculated glomerular filtration rate ≥40 mL/min/1.73 m^2 (Cockcroft-Gault)" to "either a serum creatinine ≤1.5 mg/dL or a calculated glomerular filtration rate ≥60 mL/min/1.73 m^2 (Cockcroft-Gault)".
**Rationale:** Inclusion criterion 18 was modified to extend the period of contraceptive use from 3 months after the study to 6 months.	
Section 4.1, Inclusion Criteria	The following statement was changed from "must agree to continue to use the same two methods of contraception throughout the study and for 3 months thereafter" to "must agree to continue to use the same two methods of contraception throughout the study and for 6 months thereafter".
**Rationale:** To add a prohibition (#6) for yellow fever vaccination.	
Section 4.3, Prohibitions and Restrictions	The following prohibition was added: "Subjects may not receive yellow fever vaccine during this study".
**Rationale:** To clarify BRCA testing procedures when the mutation status of BRCA 1 or BRCA 2, and not both, is known.	
Section 9.4.1, Mandatory BRCA 1 and BRCA 2 Mutation Analysis	The following phrase was deleted from the first sentence of the first paragraph in Section 9.4.1: "for subjects who have previously been tested for BRCA 1/2 mutations".<br><br>The following paragraph was added to the end of Section 9.4.1: "For subjects with a known mutation status for either BRCA 1 or BRCA 2, but not for both genes, the result from the single gene tested will be captured in the CRF. If the status of the reported gene indicates a mutation, the status will be used for randomization. However, if the single gene tested indicates no mutation, then BRCA testing for the other gene is to be conducted and reported within 30 days prior to randomization."
**Rationale:** To reflect the recent United States Food and Drug Administration approval of generic doxorubicin hydrochloride (HCl) liposome injection, and it's potential to be used in the United States of America (USA) as a treatment for ovarian cancer.	
Throughout the protocol	DOXIL refers to DOXIL®/doxorubicin hydrochloride (HCl) liposome injection (USA only) and CAELYX®/pegylated liposomal doxorubicin (PLD) (non-USA).
**Rationale:** To change the EQ-5D-3L Health Questionnaire to the current EQ-5D-5L Health Questionnaire	
Attachment 4, EQ-5D-5L Health Questionnaire	The 3L questionnaire was deleted and the 5L questionnaire was added.

Applicable Section(s)	Description of Change(s)
**Rationale:** To simplify the Time and Events Schedule.	
Time and Events Schedule	The randomization column was deleted.
**Rationale:** To change the wording regarding clinically significant abnormalities persisting during follow-up.	
Time and Events Schedule; Section 9.1.4, Posttreatment Phase (Follow-Up); Section 9.5, Safety Evaluations	Text was changed to indicate that clinically significant abnormalities persisting 30 days after the last dose of study drug would be followed, rather than clinically significant abnormalities persisting at the end of the study.
**Rationale:** To update the text regarding the study card.	
Section 12.3.1, All Adverse Events	The information to be listed on the "wallet (study) card" was revised. Text instructing the subject to carry the "wallet (study) card" with them for the duration of the study was added.
**Rationale:** To update the title page.	
Title page	Document and version number provided. Approval date changed.
**Rationale:** Minor edits for clarity and consistency.	
Throughout the protocol	Minor edits were made throughout the protocol.

Amendment INT-1 (25 March 2013)

This amendment is considered to be substantial based on the criteria set forth in Article 10(a) of Directive 2001/20/EC of the European Parliament and the Council of the European Union.

The overall reason for the amendment: The overall reason for the amendment is to revise inclusion criterion #6.

Applicable Section(s)	Description of Change(s)
**Rationale:** To modify the subject inclusion criterion #6 and to clearly identify the subject population.	
Synopsis; Section 4.1, Inclusion Criteria	The stipulation that progression of disease must occur ≥6 months from the start of second-line therapy was deleted from inclusion criterion #6. The bullet regarding second-line therapy for subjects treated with a pegylated liposomal doxorubicin was changed to "Subjects treated with a pegylated liposomal doxorubicin-containing regimen as a second-line therapy are eligible if subsequent disease progression occurs ≥9 months from the first dose".
**Rationale:** To add text regarding the rare incidence of secondary oral cancer after the long-term exposure to DOXIL.	
Section 1.3, Comparator DOXIL (doxorubicin HCL)	Text was added regarding rare cases of secondary oral cancer after long-term exposure to DOXIL.

Applicable Section(s)	Description of Change(s)
**Rationale:** To clarify the timing of the follow-up survival.	
Synopsis; Time and Event Schedule; Section 3.1, Overview of Study Design; Section 9.1.4, Posttreatment Phase (Follow-up)	Text was modified to state: Subjects will be followed for survival at least every 8 weeks for the first 2 years after the treatment termination visit and approximately every 12 weeks thereafter.
**Rationale:** To specify that subjects who complete 2 cycles of treatment after confirmed CR should discontinue treatment.	
Synopsis, Overview of Study Design; Section 3.1, Overview of Study Design	The following text was revised to include the condition noted in Section 10.2: "Treatment will continue until the occurrence of disease progression or unacceptable treatment toxicity, or until 2 cycles beyond a confirmed complete response (CR)."
Section 10.2, Discontinuation of Treatment	The following condition was added: "The subject completes 2 cycles of treatment after confirmed CR".
**Rationale:** To add pegylated liposomal doxorubicin (PLD) to the text.	
Section 1.3, Comparator – DOXIL (pegylated liposomal doxorubicin)	Pegylated liposomal doxorubicin (PLD) was added to the description of DOXIL.
**Rationale:** To change the blood sample collection for the optional pharmacogenomics analysis from randomization to Day 1 of Cycle 1.	
Time and Events Schedule; Section 9.1.2, Screening Phase	The time and events schedules were modified and the footnotes updated to indicate "The optional pharmacogenomics blood sample collection is scheduled for Day 1 of the first cycle of treatment. However, it will not be considered a protocol deviation if the blood sample is collected at any time following this timepoint."<br>In Section 9.1.2, text regarding the optional pharmacogenomics portion of the study was updated to note that the pharmacogenomics blood sample collection is scheduled for Day 1 of Cycle 1.
**Rationale:** To update the time interval for reporting PQCs.	
Section 13.1, Procedures	The reporting of PQCs was changed from as soon as possible to within 24 hours.
**Rationale:** To update the title page.	
Title page	Document and version number provided. Approval date changed.
**Rationale:** Minor edits.	
Throughout the protocol	The OV28 questionnaire designation was changed to QLQ-OV28. In addition, there are minor edits throughout the document.

SYNOPSIS

A Randomized, Open-Label Study Comparing the Combination of YONDELIS® and DOXIL®/CAELYX® With DOXIL®/CAELYX® Monotherapy for the Treatment of Advanced-Relapsed Epithelial Ovarian, Primary Peritoneal, or Fallopian Tube Cancer

EudraCT NUMBER: 2012-004808-34

Trabectedin (YONDELIS®) is a tris, tetrahydroisoquinoline alkaloid initially isolated from the marine ascidian ecteinascidin turbinate. It has a unique mechanism of action that involves the transcription-dependent nucleotide excision repair system and is currently under development as an antineoplastic agent. Trabectedin has been approved in the European Union and other countries outside the United States of America (USA) for the treatment of soft tissue sarcoma (STS) and ovarian cancer, and it has been approved in the USA for the treatment of unresectable or metastatic liposarcoma or leiomyosarcoma in patients who received a prior anthracycline-containing regimen.

Trabectedin and DOXIL (as per protocol, DOXIL refers to DOXIL®/doxorubicin hydrochloride [HCl] liposome injection [USA only] and CAELYX®/pegylated liposomal doxorubicin [PLD] [non-USA]), 2 agents with different mechanisms of action, have shown efficacy in treating women with advanced ovarian cancer. The combination of trabectedin and DOXIL has previously been demonstrated to improve progression-free survival (PFS), as compared with DOXIL monotherapy, in the second-line treatment of refractory ovarian cancer. Median overall survival (OS) was also increased by 3.3 months in the combined trabectedin+DOXIL treatment arm, but this result did not reach statistical significance (p=0.0835). Retrospective analysis suggested that the efficacy of the trabectedin+DOXIL combination was greatest in subjects with platinum-sensitive disease (subjects with a platinum-free interval ≥6 months), and that the lack of statistical significance for OS may have resulted from an imbalance in prognostic factors. Thus, limiting treatment to patients with platinum-sensitive disease is expected to result in improved demonstrated efficacy, as well as an improved risk-benefit ratio.

Currently, there are few prospective studies that have examined the third-line treatment of patients with refractory epithelial ovarian cancer. Study ET743-OVC-3006 is designed to investigate the activity of trabectedin+DOXIL for the treatment of subjects with platinum-sensitive advanced-relapsed epithelial ovarian, primary peritoneal, or fallopian tube cancer who have received 2 previous lines of platinum-based chemotherapy.

OBJECTIVES AND HYPOTHESIS

Primary Objective

To compare the OS after treatment with trabectedin+DOXIL combination therapy to that observed after treatment with DOXIL monotherapy for subjects with platinum-sensitive advanced-relapsed epithelial ovarian, primary peritoneal, or fallopian tube cancer who have received 2 previous lines of platinum-based chemotherapy.

Secondary Objectives

- To evaluate PFS.
- To evaluate the objective response rate (ORR).
- To characterize the plasma pharmacokinetics of trabectedin using a sparse sampling scheme in the trabectedin+DOXIL treatment group.
- To evaluate the safety of the trabectedin+DOXIL combination therapy and DOXIL monotherapy.

Exploratory Objectives

- To conduct pharmacogenomic evaluations of OS, PFS and other endpoints in subjects with and without mutations in BRCA1 or BRCA2.
- To evaluate patient-reported outcomes (PROs).

Hypothesis

Trabectedin in combination with DOXIL will improve OS compared with DOXIL monotherapy in the treatment of subjects with platinum-sensitive advanced-relapsed epithelial ovarian, primary peritoneal, or fallopian tube cancer who received 2 previous lines of platinum-based chemotherapy.

OVERVIEW OF STUDY DESIGN

This is a randomized, open-label, active-controlled, multicenter study designed to assess the efficacy and safety of trabectedin+DOXIL as a third-line chemotherapy in subjects with platinum-sensitive advanced-relapsed epithelial ovarian, primary peritoneal, or fallopian tube cancer who received 2 previous lines of platinum-based chemotherapy. Approximately 670 subjects will be enrolled in this study, with 335 subjects per planned treatment group.

During the Screening Phase, potential subjects will be assessed for study eligibility after providing informed consent to participate in the study. Baseline radiographic disease assessments must be performed within 30 days before randomization.

At randomization, subjects will be stratified by 4 criteria: 1) the time from the last dose of first-line platinum therapy to disease progression (6 months to 12 months vs >12 months to 24 months vs >24 months), 2) Eastern Cooperative Oncology Group (ECOG) performance status grade (0 vs 1), 3) BRCA 1/2 status (mutation vs no mutation), and 4) prior DOXIL therapy (no vs yes). Subjects will then be randomly assigned in a 1:1 ratio to the trabectedin+DOXIL combination therapy group (Arm A) or to the DOXIL monotherapy group (Arm B).

During the Treatment Phase, subjects will receive study drug by IV infusion on Day 1 of a 21-day cycle in Arm A and on Day 1 of a 28-day cycle in Arm B. Treatment will continue until the occurrence of disease progression or unacceptable treatment toxicity, or until 2 cycles after assessment of a complete response (CR). Subjects assigned to the trabectedin+DOXIL group (Arm A) will be pretreated with 20 mg of IV dexamethasone (or an equivalent IV corticosteroid) approximately 30 minutes prior to initiation of infusion of DOXIL IV, on Day 1 of each treatment cycle. Treatment efficacy endpoints will be evaluated using Response Evaluation Criteria in Solid Tumors (RECIST, Version 1.1). Scheduled assessments of disease status will be performed within 30 days before randomization, every 8 weeks (±5 days) after randomization for the first 4 assessments, and then every 12 weeks (±5 days) thereafter. Disease assessments, including assessments for subjects who discontinue treatment for reasons other than disease progression, will be performed until disease progression is radiographically confirmed, the start of subsequent anticancer therapy, withdrawal of subject consent, or the clinical cutoff date. For subjects discontinuing study treatment, documentation of all subsequent anticancer therapy, survival status, and safety evaluations as outlined in the Time and Events Schedule will be required. Survival status follow-up should be done at least every 8 weeks for the first 2 years after the treatment termination visit and approximately every 12 weeks thereafter. Collection of survival status will continue until at least 514 deaths have been observed or until the clinical data cutoff date. Treatment safety will be assessed using laboratory test results and the incidence and severity of adverse events. Left ventricular ejection fraction (LVEF) assessments (either multigated acquisition [MUGA] scans or 2-dimensional echocardiograms [2D-ECHO]) and electrocardiograms (ECGs) will be used to monitor cardiac function.

An Independent Data Monitoring Committee (IDMC) will assess safety and efficacy data. Safety data will be reviewed approximately every 6 months. An interim analysis of OS will be performed after

approximately 308 subjects have died. The final analysis of OS will occur when approximately 514 deaths have been observed or until the clinical cutoff date. A study steering committee composed of physicians will periodically review issues related to study conduct and offer advice as needed.

On 26 June 2017, the IDMC requested a futility analysis of OS to be performed at the time of the next meeting 6 months later. Following the review of the study data by the IDMC on 15 December 2017, the HR for OS was 0.962, crossing the previously agreed upon threshold for futility of 0.93. In view of this result and the observed and expected higher toxicity in Arm A as compared with Arm B, the IDMC recommended discontinuing the study. Therefore, as of Amendment 6, no new subjects will be randomized to study treatment, and treatment with trabectedin should be immediately discontinued for subjects assigned to Arm A (trabectedin+DOXIL). All study subjects (Arm A or Arm B) currently on study who, in the opinion of the investigator, are deriving clinical benefit may continue treatment with single-agent DOXIL as per the local standard of care. Treatment for these subjects may continue as long as the subjects comply with protocol-specified prohibitions and restrictions for treatment and as long as they experience clinical benefit in the opinion of the investigator. DOXIL will be provided by JR&D as needed until post-trial access to DOXIL is available or until disease progression, whichever occurs first.

Accordingly, the end of study data collection is defined as when all subjects on study treatment have completed treatment termination visit assessments as specified in the Time and Events Schedule for Amendment 6 or by 18 January 2018, whichever occurs first. For subjects continuing treatment with single-agent DOXIL, as per the local standard of care, only serious adverse events should be reported to JR&D.

SUBJECT POPULATION

Eligible women must fulfill all study inclusion and exclusion criteria. Subjects will be at least 18 years of age at screening, with histologically proven advanced-relapsed epithelial ovarian cancer, fallopian tube cancer, or primary peritoneal cancer. Subjects must have:

- ECOG performance status Grade 0 or 1.
- First-line treatment with a platinum-based regimen, and no evidence of disease progression for ≥6 months after the last dose.
- Second-line treatment with a platinum-based regimen, with progression of disease after attaining a CR or partial response (PR). Complete response or PR could have been determined by imaging or by serum CA-125 levels.
- Progression of disease based on imaging after the second-line platinum-based regimen.
  - Subjects treated with a DOXIL-containing regimen as a second-line therapy are eligible if subsequent disease progression occurs ≥9 months from the first dose.
- No more than 2 prior lines of systemic therapy.

DOSAGE AND ADMINISTRATION

Study treatment occurs on Day 1 (+2 days) of each 21-day cycle for subjects assigned to Treatment Arm A and on Day 1 (+2 days) of each 28-day cycle for subjects assigned to Treatment Arm B.

- **Treatment Arm A**: DOXIL 30 mg/m^2 administered as an IV infusion over approximately 90 minutes (per package insert) followed by trabectedin 1.1 mg/m^2 administered as an IV infusion over approximately 3h, via central venous access, every 3 weeks. Central venous access is required for administration of trabectedin. If no infusion-related reaction is observed with DOXIL after the first infusion, DOXIL may be administered as a 60- to 90-minute infusion in subsequent cycles. All subjects assigned to Treatment Arm A will be premedicated with 20 mg dexamethasone, administered IV approximately 30 minutes before the start of the DOXIL infusion, to reduce the

incidence of transaminase elevation related to trabectedin. If dexamethasone is not available, an equivalent IV dose of another corticosteroid may be substituted.

- **Treatment Arm B:** DOXIL, 50 mg/m^2 administered as an IV infusion over approximately 90 minutes (per package insert) every 4 weeks. If no infusion-related reaction is observed with DOXIL after the first infusion, DOXIL may be administered as a 60- to 90-minute infusion in subsequent cycles.

Investigators will review physical findings, adverse events, and the results of clinical laboratory tests before each dose of study drug and, if required, delay administration or reduce the dose according to the protocol-defined guidelines.

EFFICACY EVALUATIONS/ENDPOINTS

Efficacy will be assessed by a determination of OS, PFS, and ORR. Measurable disease and the response criteria based on measurable disease used in this protocol are defined in the RECIST (Version 1.1) guidelines. Radiological disease assessments (thoraco-abdominal and pelvic computed tomography [CT] scan or magnetic resonance imaging [MRI] measurements and evaluation of extent of disease) will be performed within 30 days before randomization, every 8 weeks (±5 days) after randomization for the first 4 assessments, and then every 12 weeks (±5 days) thereafter. Disease assessments, including assessments for subjects who discontinue treatment for reasons other than disease progression, will be performed until disease progression is radiographically confirmed, the start of subsequent anticancer therapy, withdrawal of subject consent, or the clinical cutoff date.

Assessments must be done consistently in both treatment arms to ensure an unbiased assessment of tumor response and progression. The CA-125 analyses will be performed at a local laboratory. The initial CA-125 evaluation will be performed within 30 days before randomization, approximately every 8 weeks after randomization for the first 4 evaluations, and then approximately every 12 weeks until disease progression is radiographically confirmed, the start of subsequent anticancer therapy, withdrawal of subject consent, or the clinical cutoff date. CA-125 evaluations should coincide (±5 days) with the scheduled tumor assessments. Survival follow-up will continue for all enrolled subjects until at least 514 deaths are observed or until the clinical cutoff date.

As of Amendment 6, follow-up data will no longer be collected.

PHARMACOKINETIC EVALUATIONS

Sparse samples for measurement of trabectedin concentrations in plasma will be collected from at least 40 subjects administered trabectedin+DOXIL.

PHARMACOGENOMIC EVALUATIONS

BRCA 1/2 mutation data will be collected at screening. If BRCA 1/2 mutation status has not been determined, a blood sample (10 mL) will be collected for BRCA 1/2 analysis. In addition, an optional pharmacogenomic blood sample (4 mL) will be collected from consenting subjects to allow for pharmacogenomic research (where local regulations permit).

SAFETY EVALUATIONS

The incidence and severity of adverse events will be assessed for all subjects who receive study drug. Clinical laboratory test results will be evaluated before dosing (ie, within 2 days prior to dosing) on Day 1 of each cycle. Blood samples will also be collected for clinical laboratory testing on +/- 2 days on Days 8 and 15 (Treatment Arm A) or Days 8, 15, and 22 (Treatment Arm B) of each cycle and at the end of treatment. Electrocardiograms and LVEF assessments (either MUGA scans or 2D-ECHO) will be performed for all subjects at screening (baseline), after Cycle 3 for treatment Arm A and after Cycle 2 for treatment Arm B, and as part of end-of-treatment assessments. In addition, those subjects who receive a

total cumulative dose of anthracycline exceeding 300 mg/m^2 (including previous and on-study treatments) or have a clinically significant history of cardiomyopathy should have follow-up LVEF assessments after every 2 cycles of therapy, or as clinically warranted.

Following the IDMC recommendation to stop the study (clinical cutoff date of 18 January 2018), only serious adverse events for subjects continuing to receive single-agent DOXIL should be reported to JR&D.

STATISTICAL METHODS

Sample Size Justification

It is assumed that failure will follow an exponential distribution with a constant hazard rate. Assuming a median OS of 16 months for the active control group (DOXIL monotherapy), a planned sample size of approximately 670 subjects will provide 80% power to detect a hazard ratio (HR) of 0.78 (16 months vs 20.5 months) at a 2-tailed significance level of 0.05 and an enrollment duration of approximately 52 months (13 subjects/month enrollment) over a total study duration of 64 months to obtain the required 514 events.

Efficacy Endpoints

All efficacy endpoints will be analyzed using the All Randomized population. The Kaplan-Meier product limit method and un-stratified Cox proportional hazards model will be used to estimate the time-to-event variables and to obtain the HR and their 95% confidence intervals. The un-stratified log-rank test will be used as the primary analysis for treatment comparison. The response rate variables will be evaluated using the Chi-square statistic (un-stratified). Sensitivity analyses for the primary endpoint using the stratified log-rank test will also be performed. Subgroup analyses will be carried out to assess if the treatment effect is consistent across clinically relevant subgroups.

TIME AND EVENTS SCHEDULE (No Longer Applicable After Amendment 6) - TREATMENT ARM A (30 MG/M^2 DOXIL ADMINISTERED AS AN IV INFUSION OVER APPROXIMATELY 90 MINUTES FOLLOWED BY 1.1 MG/M^2 TRABECTEDIN ADMINISTERED AS AN IV INFUSION OVER APPROXIMATELY 3 HOURS EVERY 3 WEEKS)

Phase	Screening[a]	Each Treatment Cycle							
Period	Within 30 days prior to randomization	Dosing on Day 1 (with a dosing window of +2 days) of each 21-day treatment cycle. Each treatment cycle being at least 21 days apart.			Cycle 3 (within the 21 day period after Cycle 3 Day 1)	Every 2 cycles when the cumulative dose of anthracyclines exceeds 300 mg/m^2	First 4 evaluations every 8 wks after randomization and then every 12 wks until disease progression	Treatment Termination Visit (30 to 33 days after the last dose)	Follow-up Phase
Day		Day 1	Day 8	Day 15					
**Study Procedure**									
Screening/Administrative									
Informed consent (ICF)	X								
Pharmacogenomic ICF (optional)	X								
Inclusion/exclusion criteria	X								
Medical history and demographics	X								
Prestudy therapy	X								
Preplanned surgery/procedure(s)	X								
Pregnancy test	X								
Study Drug Administration									
Randomization	X								
Body Surface Area (BSA)[b]	X	X							
Premedication with dexamethasone (20 mg IV) or an equivalent IV corticosteroid approximately 30 min prior to DOXIL. Combination Arm A ONLY		X							
DOXIL 30 mg/m^2, IV infusion, approximately 90 min		X							
Trabectedin 1.1 mg/m^2, IV infusion, approximately 3h		X							

Phase	Screening[a]	Each Treatment Cycle							
Period	Within 30 days prior to randomization	Dosing on Day 1 (with a dosing window of +2 days) of each 21-day treatment cycle. Each treatment cycle being at least 21 days apart.			Cycle 3 (within the 21 day period after Cycle 3 Day 1)	Every 2 cycles when the cumulative dose of anthracyclines exceeds 300 mg/m^2	First 4 evaluations every 8 wks after randomization and then every 12 wks until disease progression	Treatment Termination Visit (30 to 33 days after the last dose)	Follow-up Phase
Day		Day 1	Day 8	Day 15					
**Study Procedure**									
Safety Assessments									
Physical examination	X								
Vital signs	X								
ECOG Performance Status	X							X	
ECG[c]	X							X	
LVEF assessment[c]	X[c]				X[c]	X[c]		X[c]	X[c]
Efficacy Assessments									
Tumor assessments	X[d]						X[d]	X[d]	X[d]
Survival Status									At least every 8 weeks for the first 2 years after the treatment termination visit, then ≈ every 12 weeks
Subsequent anticancer therapy									X
Patient Reported Outcomes (PRO) – Always complete before any tests, procedures, drug administration, or other consultations									
QLQ-OV28		X						X	
EQ-5D-5L		X						X	≈ every 8 weeks until the start of subsequent anticancer treatment

Phase	Screening[a]	Each Treatment Cycle							
Period	Within 30 days prior to randomization	Dosing on Day 1 (with a dosing window of +2 days) of each 21-day treatment cycle. Each treatment cycle being at least 21 days apart.			Cycle 3 (within the 21 day period after Cycle 3 Day 1)	Every 2 cycles when the cumulative dose of anthracyclines exceeds 300 mg/m^2	First 4 evaluations every 8 wks after randomization and then every 12 wks until disease progression	Treatment Termination Visit (30 to 33 days after the last dose)	Follow-up Phase
Day		Day 1	Day 8	Day 15					
**Study Procedure**									
Clinical Laboratory Assessments - Obtain within 2 days prior to drug administration on Day 1 and +/- 2 days on Days 8 and 15 of each treatment cycle									
CA-125	X						X	X	X[i]
Hematology	X	X	X	X				X	
Chemistry and Liver Panel	X	X	X	X				X	
Pharmacokinetics									
Blood sample collection		X[e]	X[e]						
Pharmacogenomics									
Blood sample collection<br>Mandatory for subjects who do not have a BRCA 1/2 status at screening.<br>A separate blood sample for subjects who consent to the optional pharmacogenomics research.	X	X[f]							
Ongoing Subject Review									
Concomitant therapy[g]	X	X	X	X			X	X	
Adverse events[g,h]	X	X	X	X			X	X	

BMI=body mass index; BSA=body surface area; ECG-electrocardiogram, ECOG=Eastern Cooperative Oncology Group; ICF=informed consent; LVEF=left ventricular ejection fraction; QLQ=quality of life questionnaire.

[a] It is expected that BRCA 1/2 analysis will require approximately 10 to 14 days. In the case of delayed BRCA 1/2 mutation reporting, the screening period may be extended for up to 14 days (with medical monitor approval). In such cases, only screening clinical laboratory assessments (hematology, clinical chemistry, and liver panel) will need to be repeated.

[b] Body surface area (BSA) to be calculated either during Screening or on Day 1 of Cycle 1. It is not necessary to recalculate BSA each cycle unless required to comply with institutional guidelines or a subject has a weight gain or loss of approximately >10% of body weight. For those subjects with a body mass index (BMI) ≥30, the use of ideal body weight for dose calculation and capping of BSA utilized for dosing calculations will be left to the discretion of the investigator and to institutional guidelines.

[c] For the left ventricular ejection fraction (LVEF) assessment, multigated acquisition scans (MUGA) or 2-dimensional echocardiograms (2D-ECHO) can be used. However, the same procedure should be used to assess LVEF during the study. The screening LVEF assessment and ECG must be performed within 30 days before randomization, and the LVEF must be within normal range according to the institutional guidelines. Each subject must also have a LVEF assessment after Cycle 3 and at the treatment termination visit. Subjects' who have received a cumulative dose of anthracyclines that exceeds 300 mg/m^2 (including previous and on-study treatments) or have a clinically significant history of cardiomyopathy, must have a follow-up LVEF assessment after every 2 cycles of therapy. In addition, subjects experiencing significant LVEF decline (defined as absolute decrease ≥15%, or less than lower limit of normal and absolute decrease ≥5%) not recovered to less than Grade 2 (or baseline) by the end of treatment should have follow up assessments of LVEF every 2 months until recovered or up to 6 months after discontinuation of study treatment (whichever occurs first).

[d] Perform disease assessments using the same radiographic technique (computed tomography [CT] scans or magnetic resonance imaging [MRI]) every 8 weeks (±5 days) after randomization for the first 4 assessments and then every 12 weeks (±5 days) until disease progression is radiographically confirmed, the start of subsequent anticancer therapy, withdrawal of subject consent, or the clinical cutoff date. The disease assessments should include radiographic imaging of the chest (with lung views), abdomen, and pelvis.

[e] Plasma samples obtained within 1 h before the start of trabectedin infusion, at 1 h after start of infusion, at 10 minutes before the end of infusion, and 0.5 h, 24 h and 168 h after the end of infusion on cycle 1 only.

[f] The optional pharmacogenomics blood sample collection is scheduled for Day 1 of the first cycle of treatment. However, it will not be considered a protocol deviation if the blood sample is collected at any time following this timepoint.

[g] To be reported from the time the informed consent document is signed until 30 days after the last dose of study drug.

[h] Any clinically significant abnormalities persisting 30 days after the last dose of study drug will be followed by the investigator until resolution or until a clinically stable endpoint is reached. Drug-related Grade 3 or Grade 4 toxicities will be monitored until Grade 2 or less, or for a maximum of 6 months after the last dose of study drug, whichever, occurs first. Grade 2 to 4 liver or cardiac toxicities will be monitored until Grade 1 or less, or for a maximum of 6 months after the last dose of study drug, whichever occurs first.

[i] For subjects who terminate treatment prior to disease progression, CA-125 samples will continue to be collected during the follow-up phase approximately every 8 weeks for the first 4 evaluations and then approximately every 12 weeks until disease progression is radiographically confirmed, the start of subsequent anticancer therapy, withdrawal of subject consent, or the clinical cutoff date. CA-125 sample collections should coincide (±5 days) with the scheduled tumor assessments.

TIME AND EVENTS SCHEDULE (No Longer Applicable After Amendment 6) - TREATMENT ARM B (50 MG/M^2 DOXIL ADMINISTERED AS AN IV INFUSION OVER APPROXIMATELY 90 MINUTES EVERY 4 WEEKS)

Phase	Screening[a]	Each Treatment Cycle								
Period	Within 30 days prior to randomization	Dosing on Day 1 (with a dosing window of +2 days) of each 28-day treatment cycle. Each treatment cycle being at least 28 days apart.				Cycle 2 (within the 28 day period after Cycle 2 Day 1)	Every 2 cycles when the cumulative dose of anthracyclines exceeds 300 mg/m^2	First 4 evaluations every 8 wks after randomization and then every 12 wks until disease progression	Treatment Termination Visit (30 to 33 days after the last dose)	Follow-up Phase
Day		Day 1	Day 8	Day 15	Day 22					
**Study Procedure**										
Screening/Administrative										
Informed consent(ICF)	X									
Pharmacogenomic ICF (optional)	X									
Inclusion/exclusion criteria	X									
Medical history and demographics	X									
Prestudy therapy	X									
Preplanned surgery/procedure(s)	X									
Pregnancy test	X									
Study Drug Administration										
Randomization	X									
Body Surface Area (BSA)[b]	X	X								
DOXIL 50 mg/m^2, IV infusion, approximately 90 min		X								
Safety Assessments										
Physical examination	X									
Vital signs	X									
ECOG Performance Status	X								X	
ECG[c]	X								X	
LVEF assessment[c]	X[c]					X[c]	X[c]		X[c]	X[c]
Efficacy Assessments										
Tumor assessments	X[d]							X[d]	X[d]	X[d]

Phase	Screening[a]	Each Treatment Cycle								
Period	Within 30 days prior to randomization	Dosing on Day 1 (with a dosing window of +2 days) of each 28-day treatment cycle. Each treatment cycle being at least 28 days apart.				Cycle 2 (within the 28 day period after Cycle 2 Day 1)	Every 2 cycles when the cumulative dose of anthracyclines exceeds 300 mg/m^2	First 4 evaluations every 8 wks after randomization and then every 12 wks until disease progression	Treatment Termination Visit (30 to 33 days after the last dose)	Follow-up Phase
Day		Day 1	Day 8	Day 15	Day 22					
**Study Procedure**										
Survival Status										At least every 8 weeks for the first 2 years after the treatment termination visit, then ≈ every 12 weeks
Subsequent anticancer therapy										X
Patient Reported Outcomes (PRO) - Always complete before any tests, procedures, drug administration, or other consultations										
QLQ-OV28		X							X	
EQ-5D-5L		X							X	≈ every 8 weeks until the start of subsequent anticancer treatment
Clinical Laboratory Assessments - Obtain within 2 days prior to drug administration on Day 1 and +/- 2 days on Days 8, 15, and 22 of each treatment cycle										
CA-125	X							X	X	X[h]
Hematology	X	X	X	X	X				X	
Chemistry and Liver Panel	X	X	X	X	X				X	

Phase	Screening[a]	Each Treatment Cycle								
Period	Within 30 days prior to randomization	Dosing on Day 1 (with a dosing window of +2 days) of each 28-day treatment cycle. Each treatment cycle being at least 28 days apart.				Cycle 2 (within the 28 day period after Cycle 2 Day 1)	Every 2 cycles when the cumulative dose of anthracyclines exceeds 300 mg/m^2	First 4 evaluations every 8 wks after randomization and then every 12 wks until disease progression	Treatment Termination Visit (30 to 33 days after the last dose)	Follow-up Phase
Day		Day 1	Day 8	Day 15	Day 22					
**Study Procedure**										
Pharmacogenomics										
Blood sample collection<br>Mandatory for subjects who do not have a BRCA 1/2 status at screening.<br>A separate blood sample for subjects who consent to the optional pharmacogenomics research.	X	X[e]								
Ongoing Subject Review										
Concomitant therapy[f]	X	X	X	X	X			X	X	
Adverse events[f,g]	X	X	X	X	X			X	X	

BMI=body mass index; BSA=body surface area; ECG-electrocardiogram, ECOG=Eastern Cooperative Oncology Group; ICF=informed consent form; MUGA=multigated acquisition scan; QLQ=quality of life questionnaire.

[a] It is expected that BRCA 1/2 analysis will require approximately 10 to 14 days. In the case of delayed BRCA 1/2 mutation reporting, the screening period may be extended for up to 14 days (with medical monitor approval). In such cases, only screening clinical laboratory assessments (hematology, clinical chemistry, and liver panel) will need to be repeated

[b] Body surface area (BSA) to be calculated either during Screening or on Day 1 of Cycle 1. It is not necessary to recalculate BSA each cycle unless required to comply with institutional guidelines or a subject has a weight gain or loss of approximately >10% of body weight. For those subjects with a body mass index (BMI) ≥30, the use of ideal body weight for dose calculation and capping of BSA utilized for dosing calculations will be left to the discretion of the investigator and to institutional guidelines.

[c] For the left ventricular ejection fraction (LVEF) assessment, multigated acquisition scans (MUGA) or 2-dimensional echocardiograms (2D-ECHO) can be used. However, the same procedure should be used to assess LVEF during the study. The screening LVEF assessment and ECG must be performed within 30 days before randomization, and the LVEF must be within normal range according to the institutional guidelines. Each subject must also have a LVEF assessment after Cycle 2 and at the treatment termination visit. Subjects' who have received a cumulative dose of anthracyclines that exceeds 300 mg/m^2 (including previous and on-study treatments) or have a clinically significant history of cardiomyopathy, must have a follow-up LVEF assessment after every 2 cycles of therapy. In addition, Subjects experiencing significant LVEF decline (defined as absolute decrease ≥15%, or less than lower limit of normal and absolute decrease ≥5%) not recovered to less than

Grade 2 (or baseline) by the end of treatment should have follow up assessments of LVEF every 2 months until recovered or up to 6 months after discontinuation of study treatment (whichever occurs first).

[d] Perform disease assessments using the same radiographic technique (computed tomography [CT] scans or magnetic resonance imaging [MRI]) every 8 weeks (±5 days) after randomization for the first 4 assessments and then every 12 weeks (±5 days) until disease progression is radiographically confirmed, the start of subsequent anticancer therapy, withdrawal of subject consent, or the clinical cutoff date. The disease assessments should include radiographic imaging of the chest (with lung views), abdomen, and pelvis.

[e] The optional pharmacogenomics blood sample collection is scheduled for Day 1 of the first cycle of treatment. However, it will not be considered a protocol deviation if the blood sample is collected at any time following this timepoint.

[f] To be reported from the time the informed consent document is signed until 30 days after the last dose of study drug.

[g] Any clinically significant abnormalities persisting 30 days after the last dose of study drug will be followed by the investigator until resolution or until a clinically stable endpoint is reached. Drug-related Grade 3 or Grade 4 toxicities will be monitored until Grade 2 or less, or for a maximum of 6 months after the last dose of study drug, whichever, occurs first. Grade 2 to 4 liver or cardiac toxicities will be monitored until Grade 1 or less, or for a maximum of 6 months after the last dose of study drug, whichever occurs first.

[h] For subjects who terminate treatment prior to disease progression, CA-125 samples will continue to be collected during the follow-up phase approximately every 8 weeks for the first 4 evaluations and then approximately every 12 weeks until disease progression is radiographically confirmed, the start of subsequent anticancer therapy, withdrawal of subject consent, or the clinical cutoff date. CA-125 sample collections should coincide (±5 days) with the scheduled tumor assessments.

TIME AND EVENTS SCHEDULE – AMENDMENT 6

	Treatment Termination Visit Assessments (within 30 days after the last dose of study drug or by 18 January 2018)
**Study Procedure**	
ECOG Performance Status	X
ECG[a]	X
LVEF assessment[a]	X[a]
Tumor assessments[b]	X[b]
QLQ-OV28	X
EQ-5D-5L	X
CA-125	X
Hematology	X
Chemistry and Liver Panel	X
Concomitant therapy	X
Adverse events	X
Survival Status	
**Single-Agent DOXIL per Local Standard of Care[c]**	
Report serious adverse events[d]	

Note: See Attachment 7 for a consolidated summary study termination procedures.

ECG-electrocardiogram, ECOG=Eastern Cooperative Oncology Group; MUGA=multigated acquisition scan; QLQ=quality of life questionnaire.

[a] For the left ventricular ejection fraction (LVEF) assessment, multigated acquisition scans (MUGA) or 2-dimensional echocardiograms (2D-ECHO) can be used. However, the same procedure should be used to assess LVEF during the study. Each subject must also have a LVEF assessment at the treatment termination visit.

[b] Perform disease assessments using the same radiographic technique (computed tomography [CT] scans or magnetic resonance imaging [MRI]). The disease assessments should include radiographic imaging of the chest (with lung views), abdomen, and pelvis.

[c] Subjects continuing treatment with single-agent DOXIL as per the local standard of care. Treatment for these subjects may continue as long as the subjects comply with protocol-specified prohibitions and restrictions (Section 4.3) for treatment and as long as they experience clinical benefit in the opinion of the investigator. DOXIL will be provided by the JR&D as needed until post-trial access to DOXIL is available or until disease progression, whichever occurs first.

[d] Serious adverse events are to be reported as specified in Section 12.3.2.

ABBREVIATIONS

ALP	alkaline phosphatase
ALT	alanine aminotransferase
ANC	absolute neutrophil count
AST	aspartate aminotransferase
β-HCG	β-human chorionic gonadotropin
BMI	body mass index
BSA	body surface area
BUN	blood urea nitrogen
CI	confidence interval
CPK	creatine phosphokinase
CR	complete response
CRF	case report form
CT	computed tomography scan
D5W	5% dextrose in water
DNA	deoxyribonucleic acid
ECG	electrocardiogram
ECOG	Eastern Cooperative Oncology Group
eDC	electronic data capture
EMA	European Medicines Agency
EU	European Union
fax	facsimile
GCIG	Gynecologic Cancer Intergroup
GCP	Good Clinical Practice
G-CSF	granulocyte-colony stimulating factor
HCl	hydrochloride
HFS	hand and foot syndrome
HR	hazard ratio
HRR	homologous recombination repair
ICF	informed consent form
ICH	International Conference for Harmonisation
IDMC	Independent Data Monitoring Committee
IEC	Independent Ethics Committee
IRB	Institutional Review Board
IVRS	interactive voice response system
IWRS	interactive web response system
JR&D	Janssen Research & Development
LC-MS/MS	liquid chromatography-mass spectrometry/mass spectrometry
LLN	lower limit of normal
LVEF	left ventricular ejection fraction
MedDRA	Medical Dictionary for Regulatory Activities
MRI	magnetic resonance imaging
MUGA	multigated acquisition scan
NCI-CTCAE	National Cancer Institute – Common Terminology Criteria of Adverse Events
NER	nucleotide excision repair
NYHA	New York Heart Association
ORR	objective response rate
OS	overall survival
PD	progressive disease
PFI	platinum-free interval
PFS	progression free survival
PK	pharmacokinetics
PLD	pegylated liposomal doxorubicin
PQC	Product Quality Complaint
PR	partial response
PRO	patient-reported outcome(s)

QLQ	quality of life questionnaire
RECIST	Response Evaluation Criteria in Solid Tumors
STS	soft tissue sarcoma
SUSAR	suspected unexpected serious adverse reaction
TTP	time-to-progression
2D-ECHO	2-dimensional echocardiogram
ULN	upper limit of normal
USA	United States of America
US FDA	United States Food and Drug Administration
USP	United States Pharmacopeia
WBC	white blood cell

1. INTRODUCTION

Trabectedin (YONDELIS®) is a tris tetrahydroisoquinoline alkaloid originally isolated from the marine ascidian ecteinascidin turbinate, *Ecteinascidia turbinata*. It exerts its action by binding to the N2 position of guanine in the minor groove of deoxyribonucleic acid (DNA), unlike other DNA-binding agents that bind to the major groove. This bends the DNA towards the major groove, a property unique to this class of DNA-binding agents, thereby inhibiting inducible transcription.[12] In contrast to other DNA-binding cytotoxic agents, which are either equally or more effective in cells containing defects of the transcription-coupled nucleotide excision repair (NER) pathway, trabectedin is more effective in cells with an intact NER pathway.[6,18] Additionally, trabectedin has been proposed to have unique modulatory effects on the tumor micro-environment that has been attributed to its effect on tumor-associated macrophages and histiocytes. [1,5,7]

Trabectedin is approved in the European Union (EU) and in other countries outside the United States of America (USA) for the treatment of soft tissue sarcoma (STS,) and relapsed platinum-sensitive ovarian cancer. The requirement for platinum-sensitivity for the indication of ovarian cancer varies by country. In the USA, trabectedin is approved for the treatment of unresectable or metastatic liposarcoma or leiomyosarcoma in patients who received a prior anthracycline-containing regimen.

DOXIL®/CAELYX® is doxorubicin hydrochloride (HCl) encapsulated in STEALTH® liposomes for IV administration. Doxorubicin belongs to the anthracycline class and has a broad spectrum of antineoplastic activity. DOXIL® (also known as doxorubicin HCl liposome injection) is approved by the United States Food and Drug Administration (US FDA) and CAELYX® (also known as pegylated liposomal doxorubicin [PLD]) is approved by the European Medicines Agency (EMA) and other countries for the treatment of ovarian cancer. Generic doxorubicin HCl liposome injection was recently approved by the US FDA. Hereafter, in this document, DOXIL/doxorubicin HCl liposome injection (USA only) and CAELYX/PLD (non-USA) will collectively be referred to as DOXIL.

Trabectedin and DOXIL, 2 agents with different mechanisms of action, have shown efficacy in treating women with advanced ovarian cancer. The combination of trabectedin and DOXIL has previously been demonstrated in the ET743-OVA-301 Phase 3 study to improve progression-free survival (PFS), as compared with DOXIL monotherapy, in the second-line treatment of refractory ovarian cancer. Median overall survival (OS) was also increased by 3.3 months in the combined trabectedin+DOXIL treatment arm, but this result did not reach statistical significance ($p=0.0835$)[4]. Retrospective analysis suggested that the efficacy of the trabectedin+DOXIL combination was greatest in subjects with platinum-sensitive disease (subjects with a platinum-free interval [PFI] ≥6 months), and that the lack of statistical significance for OS may have resulted from an imbalance in prognostic factors. Thus, limiting treatment to patients with platinum-sensitive disease is expected to result in improved demonstrated efficacy, as well as an improved risk-benefit ratio.

Study ET743-OVC-3006 is designed to investigate the activity of trabectedin+DOXIL for the treatment of subjects with platinum-sensitive advanced-relapsed epithelial ovarian, primary peritoneal, or fallopian tube cancer. As second-line therapy with a platinum-based combination regimen is now considered superior to a single-agent in this patient population,[15] this study will compare the activity of trabectedin+DOXIL to that of DOXIL monotherapy in the third-line setting in subjects who have received 2 previous lines of platinum-based chemotherapy.

For the most comprehensive nonclinical and clinical information regarding the efficacy and safety of trabectedin, refer to the latest version of the Investigator's Brochure and Addenda for YONDELIS (trabectedin).[21]

The term "sponsor" used throughout this document refers to the entities listed in the Contact Information page(s), which will be provided as a separate document.

1.1. Trabectedin

Trabectedin is the first agent identified in a new cytotoxic class having a unique mechanism of action. Trabectedin binds to the minor groove of DNA. In contrast to other DNA-interacting chemotherapeutic agents, which are most active in the S phase and have an enhanced activity in NER-deficient cells, trabectedin is most active in the G1 phase and requires a functional transcription-coupled NER system to demonstrate activity. In vitro activity was observed at concentrations in the nanomolar range prompting studies in animal models and eventually evaluation in subjects with ovarian cancer.

1.1.1. Human Pharmacokinetics of Trabectedin

The pharmacokinetics (PK) of trabectedin were evaluated after administration of a 1.3 mg/m^2 3-h IV infusion to subjects with advanced breast cancer (Study ET743-INT-3).[20] Maximum plasma concentrations were generally observed at 1.5 h to 3 h after the start of the 3-h IV infusion. After the first dose in Cycle 1 (1.3 mg/m^2), mean maximum plasma concentrations were approximately 6.3 ng/mL. When the infusion was stopped, an initial rapid decline in plasma concentrations was observed, which was followed by a much slower decrease in concentrations. Mean systemic clearance rates of trabectedin ranged from 17 L/h to 69 L/h. The volume of distribution (mean: 4,860 L) was greater than total body water, indicating that trabectedin distributes extensively into peripheral tissues. The mean terminal half-life of this compound was 148 h. The range of values observed for the various PK parameters were comparable for the first 2 cycles of therapy.[21]

1.1.2. Metabolism of Trabectedin

Following dosing in humans, trabectedin undergoes extensive oxidative metabolism to numerous metabolites, most of which have not been identified. There is no evidence that trabectedin undergoes direct glucuronidation. On the basis of in vitro experiments, CYP3A4 is considered the predominant cytochrome P450 enzyme responsible for the hepatic metabolism of trabectedin.[21]

1.2. Combination Therapy - Trabectedin + DOXIL

1.2.1. Pivotal Efficacy Study

Study ET743-OVA-301 was a Phase 3, randomized, open-label, active-control, parallel-group, multicenter study designed to assess the efficacy and safety of the trabectedin+DOXIL combination therapy when used to treat subjects with advanced-relapsed epithelial ovarian cancer.[4] The study was designed to determine whether or not the non-platinum, non-taxane combination of trabectedin+DOXIL would provide an option for patients who may not be able to receive further platinum- or taxane-based therapies. Subjects received either the trabectedin+DOXIL combination therapy every 3 weeks (DOXIL 30 mg/m^2 as a 90 minute infusion followed by 1.1 mg/m^2 of trabectedin as a 3h infusion) or DOXIL as a monotherapy every 4 weeks (DOXIL 50 mg/m^2 as a 90 minute infusion). Subjects in the combination therapy arm were pretreated with IV dexamethasone (or an equivalent IV corticosteroid).

When compared with DOXIL monotherapy, the combination therapy of DOXIL and trabectedin prolonged PFS for women with advanced-relapsed epithelial ovarian cancer after failure of first-line platinum-based chemotherapy. The median PFS was 7.3 months (95% confidence interval [CI]: 5.9 to 7.9) for the trabectedin+DOXIL arm and 5.8 months (95% CI: 5.5 to 7.1) for the DOXIL monotherapy arm. The hazard ratio (HR) was 0.79 (95% CI: 0.65 to 0.96, p=0.0190) which represented a 21% risk reduction in disease progression or death in the combination arm compared with DOXIL monotherapy (Figure 1).

Figure 1: Kaplan-Meier Plot of Progression-Free Survival
(Study ET743-OVA-301: Independent Radiologist Review)

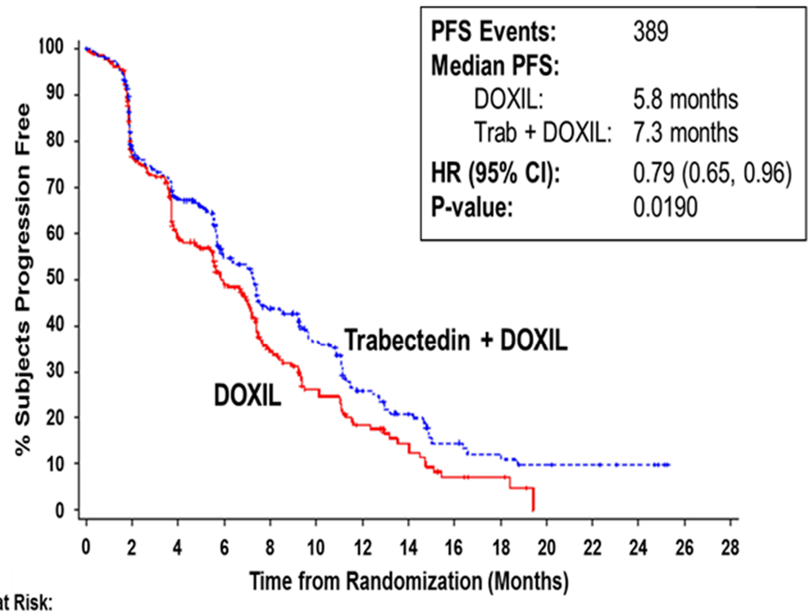

Subjects at Risk:	0	2	4	6	8	10	12	14	16	18	20	22	24	26	28
DOXIL	317	208	139	93	54	35	22	14	6	4	0	0	0	0	0
Trabectedin + DOXIL	328	225	176	121	86	63	33	22	13	10	7	6	4	0	0

Median survival was 22.2 months for the trabectedin+DOXIL arm and 18.9 months for the DOXIL arm, a 3.3 month improvement with the combination treatment. The HR was 0.86 with 95% CI: 0.72 to 1.02, and a p-value of 0.0835 (un-stratified log-rank test) (Figure 2). An unexpected but significant imbalance in PFI favoring the DOXIL arm (mean PFI: DOXIL=13.3 months; trabectedin+DOXIL=10.6 months) was identified. On the basis of this finding, a retrospective analysis adjusting for the PFI imbalance and other prognostic factors suggested an improvement in OS associated with the trabectedin+DOXIL arm (HR=0.82; 95% CI: 0.69 to 0.98; p=0.0285).

Figure 2: Kaplan-Meier Plot of Overall Survival

(Study ET743-OVA-301: All Randomized Subjects)

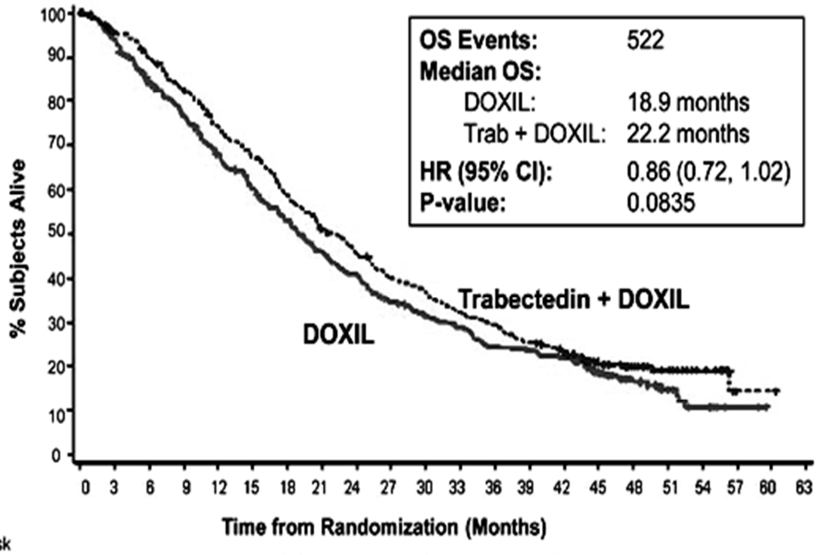

No. Subjects at Risk	0	3	6	9	12	15	18	21	24	27	30	33	36	39	42	45	48	51	54	57	60	63
DOXIL	335	311	273	246	214	190	166	143	127	106	94	87	74	71	64	39	23	12	7	2	0	0
Trab+DOXIL	337	314	293	268	240	217	190	163	145	127	116	102	93	80	68	50	33	22	15	1	1	0

Subgroups by Platinum-Free Interval

A subgroup analysis of OS by PFI (0 to 6 months, 6 to 12 months, and >12 months) was conducted for Study ET743-OVA-301 (Figure 3). Subjects with a PFI <6 months received no benefit from trabectedin+DOXIL second-line, combination chemotherapy in Study ET743-OVA-301. The benefit of the combination therapy (trabectedin+DOXIL) was evident in the subject population that was platinum sensitive (ie, patients with a PFI of 6 to 12 months and >12 months); the subset of subjects with a PFI of 6 to 12 months had the largest difference in OS (HR: 0.64; 95% CI: 0.47 to 0.86; p=0.0027).

Figure 3: Overall Survival by Platinum-Free Interval

(Study ET743-OVA-301: All Randomized Subjects Analysis Set)

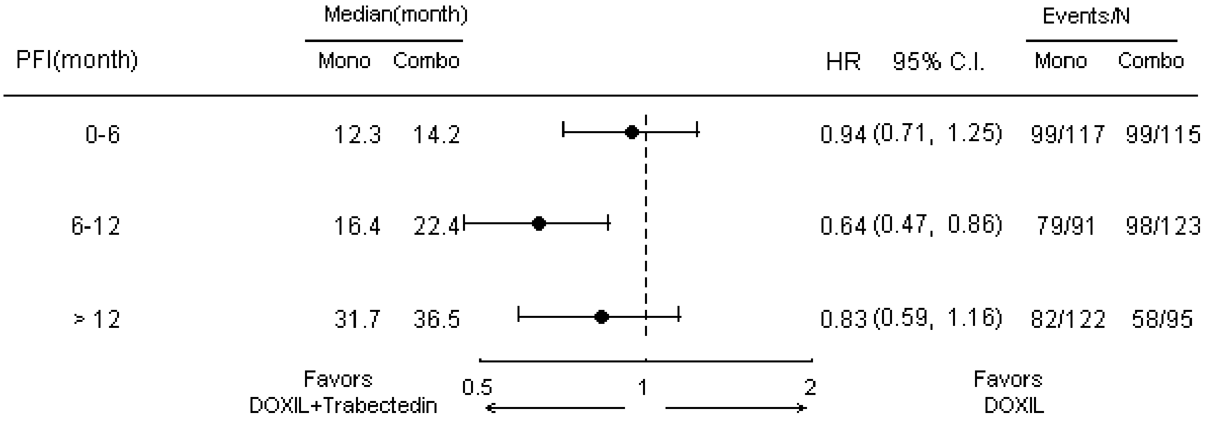

CI=confidence interval; Combo=combination therapy (trabectedin+DOXIL); HR=hazard ratio; Mono=monotherapy (DOXIL); PFI=platinum-free interval

1.2.2. Pivotal Safety Study

In Study ET743-OVA-301, the safety profile of the trabectedin+DOXIL combination therapy was consistent with the well-characterized toxicities seen with each agent alone.[4] The incidence of Grade 3 or 4 adverse events, drug-related serious adverse events, and adverse events leading to treatment termination were higher for the trabectedin+DOXIL combination arm than for the DOXIL monotherapy arm. Death due to adverse events was infrequent, occurring in 2 (1%) subjects in the DOXIL monotherapy arm and in 5 (2%) subjects in the trabectedin+DOXIL arm. No subjects developed myelodysplasias or acute myeloid leukemia during the study. Treatment with the combination of trabectedin+DOXIL primarily resulted in additional myelosuppression and liver enzyme abnormalities, both of which did not usually result in serious clinical sequelae. The incidence of Grades 3 and 4 adverse events for neutropenia was higher in the trabectedin+DOXIL arm (63%) compared with DOXIL monotherapy (23%). In the trabectedin+DOXIL arm 8% of subjects developed Grade 3 or 4 neutropenic fever compared with 2% in DOXIL monotherapy arm. Neutropenia was managed by dose delays and reductions, as well as the use of colony stimulating agents.

The incidence of Grade 3 or 4 alanine aminotransferase (ALT) was 1% in the DOXIL monotherapy arm and 32% in the trabectedin+DOXIL arm.[4] This was not unexpected, because acute, transient elevations in both ALT and aspartate aminotransferase (AST) have been associated with single-agent trabectedin. Transaminase elevations were generally of short duration, usually returning to normal before the next cycle. In addition, in most cases, it decreased in incidence and magnitude in subsequent cycles, and did not result in severe liver toxicity or failure. Transaminase elevations were generally managed by dose reductions and delays.[4,21]

The overall incidence of increased blood creatine phosphokinase (CPK) adverse events was 11 (3%) subjects in the DOXIL monotherapy arm and 24 (7%) subjects in the trabectedin+DOXIL arm. The incidence of rhabdomyolysis, a potential complication of

increased CPK, was 0% and <1% in the DOXIL monotherapy and trabectedin + DOXIL arms, respectively. The overall incidence of renal and urinary disorder adverse events was 8% of subjects in the DOXIL monotherapy arm and 10% of subjects in the trabectedin+DOXIL arm.

The incidence of cardiac dysfunction, measured as a decrease in left ventricular ejection fraction (LVEF) from baseline to treatment termination, were comparable between the trabectedin+DOXIL combination therapy arm and the DOXIL monotherapy arm (6% vs 8%, respectively). The incidence of hand and foot syndrome (HFS) (26% vs 54%), stomatitis (20% vs 33%), mucosal inflammation (13% vs 20%), and abdominal pain (22% vs 24%) were all reduced, respectively, as might be expected with the lower dose of DOXIL (30 mg/m^2) in the trabectedin+DOXIL arm compared with the DOXIL monotherapy arm (50 mg/m^2).[4]

1.3. Comparator DOXIL

DOXIL is doxorubicin HCl encapsulated in STEALTH liposomes for IV administration. Doxorubicin belongs to the anthracycline class and has a broad spectrum of antineoplastic activity. The STEALTH liposome escapes instant recognition and uptake by the mononuclear phagocyte system resulting in a longer circulation time. These liposomes eventually extravasate through the abnormally permeable vessels characteristic of many tumors. Once concentrated in tumors, DOXIL can deliver high levels of doxorubicin to malignant cells. The safety profile is improved, with less cardiotoxicity, nausea and vomiting, and alopecia compared with standard doxorubicin.

The major dose-limiting toxicity of DOXIL is bone marrow suppression. Other toxicities include HFS and irreversible cumulative cardiac toxicity that limits the total deliverable dose. Special attention must be given to the myocardial damage that may be associated with cumulative doses of doxorubicin and other anthracyclines. In addition, very rare cases of secondary oral cancer have been observed in patients with long-term exposure to DOXIL (>1 year or those receiving a cumulative dose of ≥720 mg/m^2). Refer to the package insert for safety information.

DOXIL® is approved by the US FDA for the treatment of patients with ovarian cancer whose disease has progressed or recurred after platinum-based chemotherapy. It is approved by the EMA and other countries as CAELYX® for the treatment of advanced ovarian cancer in women after failure of first-line platinum-based chemotherapy regimen. Generic doxorubicin HCl liposome injection was recently approved by the US FDA. For the treatment of ovarian carcinoma, DOXIL, as a single agent, is administered at a dose or 50 mg/m^2 every 4 weeks.

1.4. Overall Rationale for the Study

Single-agent activity of trabectedin has been studied in several Phase 1 and 2 clinical studies. Trabectedin had a favorable safety profile and promising activity in recurrent ovarian cancer patients.[10,17] Pooled analysis of 3 Phase 2 studies showed a response rate of 34% and time-to-progression (TTP) of 5.8 months in subjects who had a PFI of 6 months or more after the last platinum-based therapy.[11] No efficacy difference was seen between the patients with 2 prior lines of platinum-based therapy and patients with only 1 prior platinum-based therapy.

DOXIL has demonstrated efficacy for the treatment of metastatic ovarian carcinoma after failure of first-line chemotherapy. The long-term follow-up from a multicenter Phase 3 study comparing DOXIL with topotecan demonstrated an 18% reduction in the risk of death for subjects treated with DOXIL (median survival 62.7 weeks compared with 59.7 weeks for topotecan-treated subjects; HR: 1.216; 95% CI: 1.000 to 1.478; p=0.050). [8,9]

In the Phase 1 Study ET743-USA-11, the trabectedin+DOXIL combination therapy was administered to subjects with a variety of tumor types. The recommended dose level was trabectedin 1.1 mg/m^2 and DOXIL 30 mg/m^2, administered once every 3 weeks (q3wk).[19]

In the Phase 3 Study ET743-OVA-301, the efficacy and safety of the trabectedin+DOXIL combination therapy was assessed in subjects with first-relapsed ovarian cancer after platinum-based chemotherapy. In this study, 672 subjects were randomly assigned to receive DOXIL 30 mg/m^2 followed by a 3-h infusion of trabectedin 1.1 mg/m^2 every 3 weeks or DOXIL 50 mg/m^2 every 4 weeks as a monotherapy. The median PFS was 7.3 months with the trabectedin+DOXIL combination therapy vs 5.8 months with the DOXIL monotherapy (HR: 0.79; 95% CI: 0.65 to 0.96; p=0190). The median OS for trabectedin+DOXIL and DOXIL monotherapy arms was 22.2 months and 18.9 months, respectively (HR: 0.86; 95% CI: 0.72 to 1.02; p=0.0835 [un-stratified log-rank test]).[4,13] In an unplanned subset analysis, an unexpected but significant imbalance in the PFI favoring the DOXIL monotherapy arm was identified (mean PFI: DOXIL arm=13.3 months; trabectedin+DOXIL arm=10.6 months). On the basis of this finding, a retrospective analysis adjusting for the PFI imbalance and other prognostic factors was performed, which suggested an improvement in OS associated with the trabectedin+DOXIL arm (HR: 0.82; 95% CI:0.69 to 0.98; p=0.0285).[14] Within the trabectedin+DOXIL combination arm, neutropenia (78% vs 39%) and Grade 3 to 4 transaminase elevations (ie, ALT: 32% vs 1%) were more commonly observed, while HFS (26% vs 54%) and mucositis (13% vs 20%) were less frequently observed than within the DOXIL monotherapy arm.[13]

On the basis of prior clinical experience, the combination of non-platinum agents, trabectedin and DOXIL, is expected to provide improved efficacy compared with DOXIL monotherapy and an acceptable safety profile in subjects with platinum-sensitive advanced-relapsed epithelial ovarian, primary peritoneal, or fallopian tube cancer who have received 2 prior lines of platinum-based chemotherapy.

2. OBJECTIVES AND HYPOTHESIS

2.1. Objectives

Primary Objective

To compare the OS after treatment with trabectedin+DOXIL combination therapy to that observed after treatment with DOXIL monotherapy for subjects with platinum-sensitive advanced-relapsed epithelial ovarian, primary peritoneal, or fallopian tube cancer who have received 2 previous lines of platinum-based chemotherapy.

Secondary Objectives

- To evaluate PFS.
- To evaluate the objective response rate (ORR).
- To characterize the plasma PK of trabectedin using a sparse sampling scheme in the trabectedin+DOXIL treatment group.
- To evaluate the safety of the trabectedin+DOXIL combination therapy and DOXIL monotherapy.

Exploratory Objectives

- To conduct pharmacogenomic evaluations of OS, PFS and other endpoints in subjects with and without mutations in BRCA1 or BRCA2.
- To evaluate patient-reported outcomes (PROs).

2.2. Hypothesis

Trabectedin in combination with DOXIL will improve OS compared with DOXIL monotherapy in the treatment of subjects with platinum-sensitive advanced-relapsed epithelial ovarian, primary peritoneal, or fallopian tube cancer who received 2 previous lines of platinum-based chemotherapy.

3. STUDY DESIGN AND RATIONALE

3.1. Overview of Study Design

This is a randomized, open-label, active-controlled, multicenter study designed to assess the efficacy and safety of trabectedin+DOXIL as a third-line chemotherapy in subjects with platinum-sensitive advanced-relapsed epithelial ovarian, primary peritoneal, or fallopian tube cancer who received 2 previous lines of platinum-based chemotherapy. Approximately 670 adult subjects will be enrolled in this study, with 335 subjects per planned treatment group. At randomization, subjects will be stratified by 4 criteria: 1) the time from the last dose of first-line platinum therapy to disease progression (6 months to 12 months vs >12 months to 24 months vs >24 months), 2) Eastern Cooperative Oncology Group (ECOG) performance status grade (0 vs 1), 3) BRCA 1/2 status (mutation vs no mutation), and 4) prior DOXIL therapy (no vs yes). Subjects will then be randomly assigned in a 1:1 ratio to the trabectedin+DOXIL combination therapy group (Arm A) or to the DOXIL monotherapy group (Arm B).

During the treatment phase, subjects will receive study drug by IV infusion on Day 1 of a 21-day cycle in Arm A and on Day 1 of a 28-day cycle in Arm B. Treatment will continue until the occurrence of disease progression or unacceptable treatment toxicity, or until 2 cycles after assessment of a complete response (CR). Subjects assigned to the trabectedin+DOXIL group (Arm A) will be pretreated with 20 mg of dexamethasone IV, or an equivalent IV corticosteroid, approximately 30 minutes prior to initiation of infusion of DOXIL on Day 1 of each treatment cycle.

Subjects will be followed for survival at least every 8 weeks for the first 2 years after the treatment termination visit and approximately every 12 weeks thereafter. This study will end 2 months after the last subject has received the last dose of study drug, when 514 deaths have been observed, or until the clinical cutoff date.

The analysis of the primary endpoint, OS, will be conducted after at least 514 events (deaths) are observed or up to the clinical cutoff date. An interim analysis is planned for this study (after observing 60% [308 death events] of the total number of required [514] death events) to determine whether efficacy has been adequately demonstrated, allowing for early study termination and subject crossover from the monotherapy arm. Given the findings from Study ET743-OVA-301, which demonstrated superior PFS and a favorable survival trend for the trabectedin+DOXIL combination therapy, there are no plans to conduct a futility analysis at the interim analysis.

Secondary efficacy endpoints of PFS and ORR will be assessed using the Response Evaluation Criteria in Solid Tumors (RECIST, Version 1.1). Scheduled assessments of disease status will be performed within 30 days before randomization, every 8 weeks (±5 days) after randomization for the first 4 assessments, and then every 12 weeks (±5 days) thereafter. Disease assessments, including assessments for subjects who discontinue treatment for reasons other than disease progression, will be performed until disease progression is radiographically confirmed, the start of subsequent anticancer therapy, withdrawal of subject consent, or the clinical cutoff date. For subjects who discontinue study treatment, documentation of all subsequent anticancer therapy, survival status, and safety evaluations as outlined in the Time and Events Schedule will be required. Survival status should be recorded at least every 8 weeks for the first 2 years after the treatment termination visit and approximately every 12 weeks thereafter. Collection of survival status will continue until at least 514 deaths have been observed or until the clinical cutoff date.

The primary efficacy endpoint is OS, defined as the time between randomization and death from any cause. Secondary endpoints are PFS (defined as the time between the date of randomization and the date of disease progression or death), and ORR (defined as the proportion of subjects who achieve CR or partial response [PR]).

Safety will be assessed using laboratory test results and the incidence and severity of adverse events. Left ventricular ejection fraction assessments (either multigated acquisition [MUGA] scans or 2-dimensional echocardiograms [2D-ECHO]) and electrocardiograms (ECGs) will be used to monitor cardiac function.

An IDMC will assess safety and efficacy data (Section 11.9, Independent Data Monitoring Committee). Safety data will be reviewed approximately every 6 months. A final cardiac analysis will be conducted and a report will be provided. An interim analysis of OS will be performed after approximately 308 subjects have died. The final analysis of OS will occur when at least 514 deaths have been observed or until the clinical cutoff date.

A Study Steering Committee will periodically review issues related to study conduct and offer advice as needed. The Steering Committee is composed of physicians who have extensive

experience with both trabectedin and the treatment of ovarian cancer. The Steering Committee will meet with the Study Team periodically to review the study status and make recommendations for actions to take with regard to study recruitment, data gathering, and data review. The Steering Committee will not have access to unblinded data listings or summary analyses during the study.

Supportive care (eg, antibiotics, analgesics, transfusions) may be provided according to institutional standards. Appropriate prophylactic antiemetic regimens may be provided, as necessary.[2] No concurrent investigational agents or antineoplastic agents will be permitted.

It is anticipated that the volumes of blood drawn for each subject for routine laboratory evaluations throughout the study will be approximately 15 mL for screening serum chemistry and hematology tests; 10 mL for BRCA 1/2 testing at screening (for subjects who do not have a known BRCA 1/2 status); 4 mL for subjects consenting to the optional pharmacogenomics component of the study (where local regulations permit); 45 mL and 60 mL for treatment Arms A and B, respectively, per cycle for serum biochemistry and hematology tests; 4 mL per scheduled CA-125 sample; and 24 mL from those subjects who provide PK samples.

All subjects who discontinue study treatment will be monitored for survival. The subject will have completed the study when the study is terminated or the subject has died or has withdrawn consent for follow-up.

A diagram of the study design is provided below (Figure 4).

Figure 4: Study Design

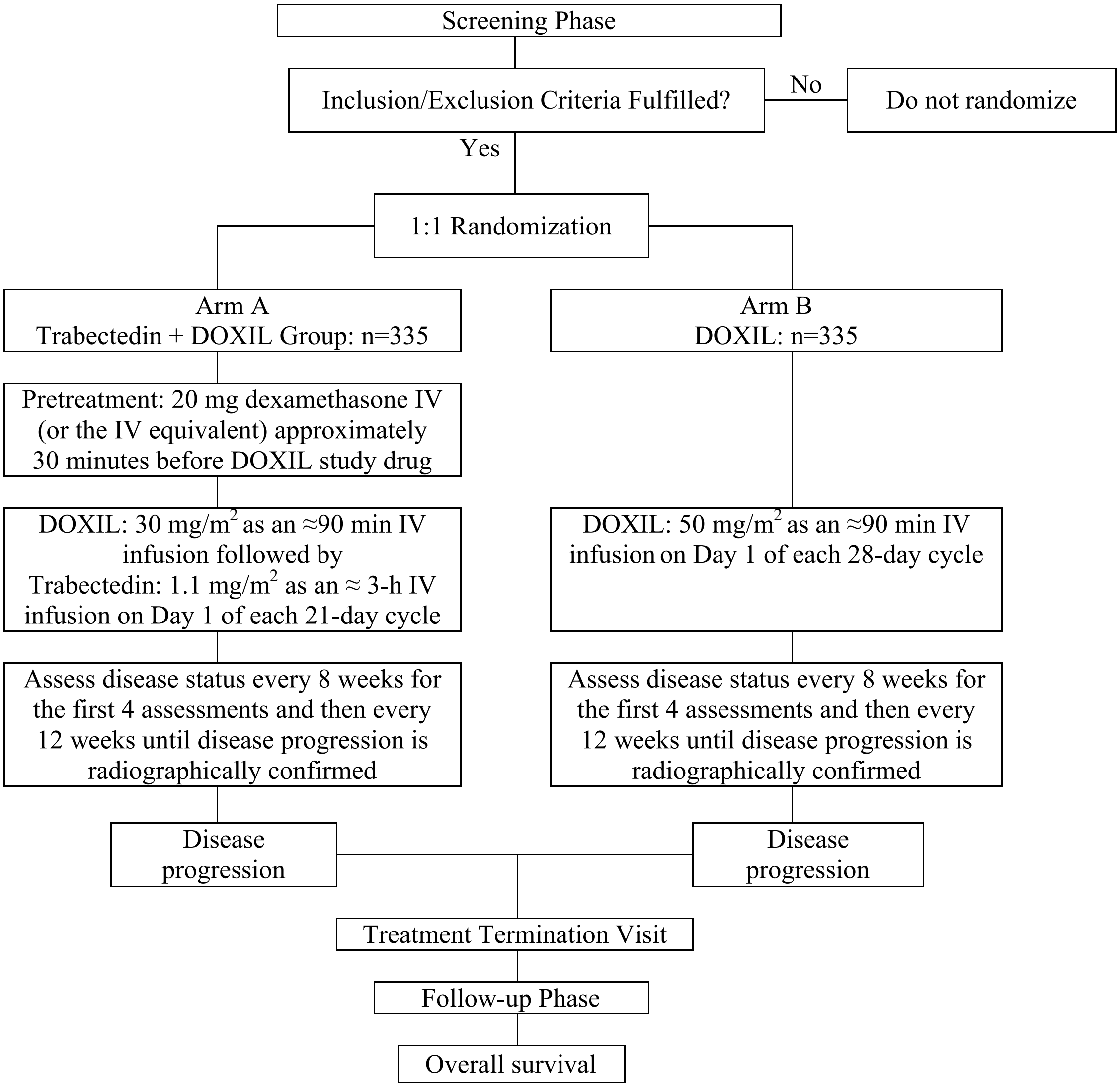

Note: On 26 June 2017, the IDMC requested a futility analysis of OS to be performed at the time of the next meeting 6 months later. In view of the IDMC request, study data up to 20 September 2017 were evaluated for safety and OS (45% rather than 60% of the required death events at the planned interim analysis). Following the review of the study data by the IDMC on 15 December 2017, the HR for OS was 0.962, crossing the previously agreed upon threshold for futility of 0.93. In view of this result and the observed and expected higher toxicity in Arm A as compared with Arm B, the IDMC recommended discontinuing the study. Therefore, as of Amendment 6, no new subjects will be randomized to study treatment, and treatment with trabectedin should be immediately discontinued for subjects assigned to Arm A (trabectedin+DOXIL). All study subjects (Arm A or Arm B) currently on study who, in the opinion of the investigator, are deriving clinical benefit may continue treatment with single-agent DOXIL as per the local standard of care. Treatment for these subjects may continue as long as the subjects comply with protocol-specified prohibitions and restrictions for treatment

(Section 4.3) and as long as they experience clinical benefit in the opinion of the investigator. DOXIL will be provided by JR&D as needed until post-trial access to DOXIL is available or until disease progression, whichever occurs first.

Accordingly, the end of study data collection is defined as when all subjects on study treatment have completed treatment termination visit assessments as specified in the Time and Events Schedule for Amendment 6 or by 18 January 2018, whichever occurs first. For subjects continuing treatment with single-agent DOXIL, as per the local standard of care, only serious adverse events should be reported to JR&D as outlined in Section 12.3.2.

3.2. Study Design Rationale

Most ovarian cancer patients are diagnosed with advanced disease, resulting in poor prognosis with inferior survival. Despite high rates of objective responses to the combination of cytoreductive surgery and platinum-based chemotherapy, disease often relapses. The results of second-line treatment depend largely on the PFI after the last dose of first-line therapy, with patients exhibiting a longer PFI having a better response to subsequent chemotherapy. Unfortunately, the response is of relatively short duration, and most patients require a third line of chemotherapy. There are very few published studies of chemotherapy administered after 2 lines of platinum-containing regimens to inform the choice of chemotherapy in the third-line setting, even though relapse of epithelial ovarian cancer is common. Study ET743-OVC-3006 is designed to investigate the activity of trabectedin+DOXIL for the treatment of subjects with platinum-sensitive advanced-relapsed epithelial ovarian, primary peritoneal, or fallopian tube cancer who have received 2 previous lines of platinum-based chemotherapy.

Randomization

Randomization will be used to minimize bias in the assignment of subjects to treatment groups, to increase the likelihood that known and unknown subject attributes (eg, demographic and baseline characteristics) are evenly balanced across treatment groups, and to enhance the validity of statistical comparisons across treatment groups.

Following Amendment 6, subjects will not be randomized to study treatment, and no new subjects will be screened. Subjects currently in screening are be eligible to receive treatment with single-agent DOXIL, if the investigator confirms that such treatment would be a clinically appropriate therapeutic option.

Pharmacokinetic Sample Collection

A population PK model has been developed using data from 15 studies performed in patients with solid malignancies, including 3 studies (ET-743-INT-11, ET-B-009-9, and ET-OVA-301) in subjects with ovarian cancer. Blood samples for population PK analysis will be collected using a sparse sampling scheme from at least 40 subjects in the trabectedin+DOXIL combination treatment group. These data will be analyzed using a population PK model to characterize the PK of trabectedin in the patient population enrolled in the present study.

BRCA 1 and BRCA 2 Mutations

The mechanisms of action of both trabectedin and DOXIL involve DNA damage. BRCA 1/2 is part of the homologous recombination repair (HRR) machinery responsible for DNA repair. This study will attempt to build on existing data by investigating the association of BRCA 1/2 mutation status with OS, PFS, and other endpoints by stratification based on BRCA 1/2 mutation status (mutation vs no mutation) prior to randomization.

4. SUBJECT POPULATION

Following Amendment 6, in those instances where the investigator confirms that single-agent treatment with DOXIL would be a clinically appropriate therapeutic option for subjects in screening, those subjects in screening would need to meet study inclusion and exclusion criteria as specified in Sections 4.1 and 4.2.

The inclusion and exclusion criteria are described in the following 2 subsections. If there is a question about the inclusion or exclusion criteria below, the investigator should consult with the appropriate sponsor representative and resolve any issues before enrolling a subject in the study. Waivers are not allowed.

Prior to randomization, inclusion criteria #4 through #8 must be reviewed by sponsor's medical monitor. Note: As of Amendment 6, subjects will not be randomized to study treatment.

4.1. Inclusion Criteria

Each potential subject must satisfy all of the following criteria to be enrolled in the study. Each subject must:

1. Be a woman 18 years of age or older.
2. Have histologically proven advanced-relapsed epithelial ovarian, primary peritoneal, or fallopian tube cancer.
3. Have an ECOG performance status grade of 0 or 1.
4. Have received first-line treatment with a platinum-based regimen and had no evidence of disease progression for ≥6 months after the last dose.
5. Criterion modified per amendment
    5.1 Have received second-line treatment with a platinum-based regimen, with progression of disease after attaining a response. A response could have been determined by imaging or by serum CA-125 levels.
6. Criterion modified per amendment
    6.1. Have progression of disease based on imaging after the second-line platinum-based regimen.
        - Subjects treated with a DOXIL-containing regimen as a second-line therapy are eligible if subsequent disease progression occurs ≥9 months from the first dose.

7. Criterion modified per amendment

   7.1 Per Amendment 6, subjects no longer need to have disease response and disease progression events as noted in criteria #5 and #6 reviewed by the sponsor's medical monitor prior to randomization.

8. Have evidence of measurable disease at screening as evaluated by RECIST (Version 1.1) criteria (Attachment 2).

9. Criterion modified per amendment

   9.1 Per Amendment 6, subjects no longer need to be able to receive IV dexamethasone or an equivalent IV corticosteroid.

10. Have a known BRCA 1/2 mutation status. For subjects who do not have a known BRCA 1/2 status at screening, a blood sample will be collected to determine the status with the results available prior to randomization.

11. Criterion modified per amendment

    11.1 Criterion modified per amendment

    11.2 Have all of the following:

    – hemoglobin ≥9 g/dL (without transfusion in the prior 7 days). Subjects may be enrolled into the study while receiving recombinant erythropoietin provided they have received recombinant erythropoietin for at least 4 weeks before the first dose of study drug.
    – albumin ≥25 g/L
    – absolute neutrophil count (ANC) ≥1,500/µL
    – platelet count ≥100,000/µL (without transfusion in the prior 7 days)
    – either a serum creatinine ≤1.5 mg/dL or a calculated glomerular filtration rate ≥60 mL/min/1.73 m^2 (Cockcroft-Gault) (Attachment 6)
    – CPK ≤2.5 x upper limit of normal (ULN)

12. Have total bilirubin ≤ULN. If total bilirubin is >ULN, measure direct and indirect bilirubin to evaluate for Gilbert's syndrome (if direct bilirubin is within normal range, subject may be eligible).

13. Have alkaline phosphatase (ALP) ≤2.5xULN; if the ALP is >2.5xULN, then an ALP liver fraction or 5' nucleotidase must be ≤ULN (as reported in absolute units of measure).

14. Have AST and ALT ≤2.5xULN.

15. Have LVEF by MUGA scan or 2D-ECHO within normal limits for the institution.

16. Have side effects (except alopecia) of prior treatment resolved to at least Grade 1 according to the National Cancer Institute – Common Terminology Criteria of Adverse Events (NCI-CTCAE) Version 4.0 (except for those laboratory criteria listed in eligibility criterion #11).

17. Have a negative pregnancy test (urinary or serum β-human chorionic gonadotropin [HCG]) at screening (applicable to women of child bearing potential).

18. Criterion modified per amendment

    18.1 Criterion modified per amendment

    18.2 Be postmenopausal (>45 years of age with amenorrhea for at least 2 years or any age with amenorrhea for at least 6 months and a serum follicle stimulating hormone level >40 IU/L or mIU/mL), surgically sterile (have had a hysterectomy or bilateral oophorectomy, bilateral salpingectomy, bilateral tubal occlusion ligation [which includes tubal ligation procedures as consistent with local regulations], or otherwise be incapable of pregnancy), abstinent (acceptability of this method is at the discretion of the investigator who will ensure and document that the subject understands the definition of "abstinence", and who will periodically remind and counsel the subject on this topic), or if heterosexually active, be practicing two effective methods of birth control (eg, prescription hormonal contraceptive, intrauterine device, double-barrier method [eg, condoms, occlusive cap (diaphragm or cervical/vault caps) with spermicidal foam, cream, gel, film, or suppository]), before enrollment, and must agree to continue to use the same two methods of contraception throughout the study and for 6 months thereafter. Note: a female condom and a male condom should not be used together as friction between the two can result in either product failing.

    Note: If the childbearing potential changes after the start of the study (eg, woman who is not heterosexually active becomes active), then the woman must begin two effective methods of birth control, as described above.

19. Be willing and able to adhere to the prohibitions and restrictions specified in this protocol. (Section 4.3, Prohibitions and Restrictions).
20. Each subject (or their legally acceptable representative) must sign an informed consent form (ICF) indicating that she understands the purpose of and procedures required for the study and is willing to participate in the study.
21. Each subject (or their legally acceptable representative) must sign a separate ICF if she agrees to provide an optional blood sample for pharmacogenomics research (where local regulations permit). Refusal to give consent for the optional research sample does not exclude a subject from participation in the study.
22. Must agree not to donate eggs (ova, oocytes) for the purposes of assisted reproduction.

4.2. Exclusion Criteria

Any potential subject who meets any of the following criteria will be excluded from participating in the study. A subject who:

1. Has a diagnosis of ovarian carcinoma with mucinous histology.
2. Criterion modified per amendment

    2.1 Had more than 2 prior lines of systemic therapy. Maintenance therapies and hormonal therapies are not considered additional lines of therapy.

3. Criterion modified per amendment

   3.1 Per Amendment 6, subjects who had a prior exposure to trabectedin or hypersensitivity to any of the excipients will not be excluded from receiving single-agent DOXIL.

4. Had prior treatment with doxorubicin or other anthracycline at cumulative doses greater than 300 mg/m^2 (calculated using doxorubicin equivalent doses: 1 mg doxorubicin = 1 mg DOXIL = 1.8 mg epirubicin = 0.3 mg mitoxantrone = 0.25 mg idarubicin).

5. Criterion modified per amendment:

   5.1 Per Amendment 6, subjects unwilling or unable to have a central venous catheter placed will not be excluded from receiving single-agent DOXIL.

6. Is pregnant or breast-feeding.

7. Criterion modified per amendment

   7.1 Would receive study treatment within 3 weeks from radiation therapy, experimental therapy, hormonal therapy, prior chemotherapy, or biological therapy; use an invasive investigational device; or is currently enrolled in an investigational study.

8. Criterion modified per amendment

   8.1 Has a history of another invasive malignancy (except non-metastatic basal cell carcinoma or squamous cell carcinoma of the skin adequately treated) unless in remission for ≥5 years, or a non-invasive malignancy requiring ongoing therapy.

9. Has known allergies, hypersensitivity, or intolerance to DOXIL, dexamethasone, or their excipients.

10. Has a known history of central nervous system metastasis.

11. Has known significant chronic liver disease, such as cirrhosis or active hepatitis (potential subjects who test positive for hepatitis B surface antigen or hepatitis C antibodies are allowed provided they do not have active disease requiring antiviral therapy).

12. Had a myocardial infarct within 6 months before enrollment, New York Heart Association (NYHA) Class II or greater heart failure (Attachment 5), uncontrolled angina, severe uncontrolled ventricular arrhythmias, clinically significant pericardial disease, or electrocardiographic evidence of acute ischemic or active conduction system abnormalities.

13. Has any of the following medical conditions:

   – Uncontrolled diabetes
   – Psychiatric disorder (including dementia) that prevents compliance with protocol
   – Uncontrolled seizures
   – Newly diagnosed deep vein thrombosis
   – Active systemic infection that is likely to interfere with study procedure or results

14. Has any condition that, in the opinion of the investigator, would compromise the well-being of the subject or the study or prevent the subject from meeting or performing study requirements.

15. Is an employee of the investigator or study site, with direct involvement in the proposed study or other studies under the direction of that investigator or study site, as well as family members of the employees or the investigator.

NOTE: Investigators should ensure that all study enrollment criteria have been met at screening. Section 17.4, Source Documentation, describes the required documentation to support meeting the enrollment criteria.

4.3. Prohibitions and Restrictions

Potential subjects must be willing and able to adhere to the following prohibitions and restrictions during the course of the study to be eligible for participation:

1. No concurrent investigational agents are permitted.
2. No concurrent antineoplastic therapy is permitted.
3. No concurrent radiotherapy is permitted.
4. No concurrent maintenance therapy for the disease being treated is permitted.
5. Subjects may not receive dexrazoxane (Zinecard) during this study.
6. Subjects may not receive yellow fever vaccine during this study.

The sponsor should be notified in advance (or as soon as possible thereafter) of any instances in which prohibited therapies are administered.

Investigators should take caution when administering inducers or inhibitors of cytochrome CYP3A4 (Section 8, Prestudy and Concomitant Therapy).

5. TREATMENT ALLOCATION AND BLINDING

Following Amendment 6, subjects will not be randomized to study treatment.

Treatment Allocation

Procedures for Stratification and Randomization

Prior to randomization, inclusion criteria #4 through #8 must be reviewed by sponsor's medical monitor (Section 4.1, Inclusion Criteria). Following sponsor review, subjects will be stratified by the time from the last dose of first-line platinum therapy to disease progression (6 months to 12 months vs >12 months to 24 months vs >24 months), ECOG performance status grade (0 vs 1), BRCA 1/2 status (mutation vs no mutation), and prior DOXIL therapy (no vs yes). Central randomization will be implemented in this study. Randomization of subjects should occur as close in time as possible before administration of the first dose of study drug(s) and must occur within 7 days of the subject receiving the first dose of study treatment. This maximum 7-day window is given to allow time for provision of central venous access for those subjects assigned to Treatment Arm A (Section 6, Dosage and Administration). Subjects will be randomly assigned, in a 1:1 ratio, to 1 of 2 treatment groups based on a computer-generated randomization schedule prepared before the study by or under the supervision of the sponsor. The interactive voice response system (IVRS) and/or interactive web response system (IWRS)

will assign a unique treatment code, which will dictate the treatment assignment and matching study drug kit for the subject. The requestor must use his or her own user identification and personal identification number when contacting the IVRS/IWRS, and will then give the relevant subject details to uniquely identify the subject.

Blinding

The requirement of a central venous catheter, limited to the combination trabectedin+DOXIL arm, precludes blinded treatment in this study. This study will therefore be conducted as an open-label study.

6. DOSAGE AND ADMINISTRATION

Janssen Research & Development (JR&D) will provide trabectedin and DOXIL as IV formulations. The amount (in mg) of trabectedin and DOXIL to be administered will be determined by subject body surface area (BSA), which will be calculated according to the standard nomogram used at each center. Body surface area will be calculated either at Screening or on Day 1 of Cycle 1. It will not be necessary to recalculate BSA at each cycle unless the institution requires it or the subject has a significant weight change (approximately >10%). For those subjects with a body mass index (BMI) ≥30, the use of ideal body weight for dose calculation and capping of BSA utilized for dosing calculations will be left to the discretion of the investigator and to institutional guidelines. The calculated dose of DOXIL may be rounded to the nearest full mg that can be administered accurately. The dose of trabectedin may be rounded to the nearest second decimal place (eg, 1.721 can be rounded to 1.72) that can be administered accurately. The first dose of study drug may be administered at any time within 7 days of randomization. This period is to allow for the placement of central venous access, if a subject is assigned to the trabectedin+DOXIL arm of the study.

Treatment Arm A: DOXIL 30 mg/m^2 administered as an IV infusion over approximately 90 minutes (per package insert) followed by trabectedin 1.1 mg/m^2 administered as an IV infusion over approximately 3h, via central venous access, every 3 weeks. Central venous access is required for administration of trabectedin. If no infusion-related reaction is observed with DOXIL after the first infusion, DOXIL may be administered as a 60- to 90-minute infusion in subsequent cycles.

All subjects assigned to Treatment Arm A will be premedicated with 20 mg dexamethasone, administered IV approximately 30 minutes before the start of the DOXIL infusion, to reduce the incidence of transaminase elevation related to trabectedin. If dexamethasone is not available, an equivalent IV dose of another corticosteroid may be substituted. For subjects in Treatment Arm A, trabectedin will be administered as an IV infusion over approximately 3h immediately after the catheter is flushed well with 5% dextrose in water (D5W). Trabectedin should be diluted in normal saline or D5W to a volume of at least 500 mL and infused via a central venous catheter. Any calibrated infusion pump may be used to deliver the drug. The administration of both drugs will be repeated every 3 weeks. The prophylactic use of granulocyte-colony stimulating factor (G-CSF) is strongly recommended for those subjects assigned to Treatment Arm A.

Treatment Arm B: DOXIL, 50 mg/m^2 administered as an IV infusion over approximately 90 minutes (per package insert) every 4 weeks. If no infusion-related reaction is observed with DOXIL after the first infusion, DOXIL may be administered as a 60- to 90-minute infusion in subsequent cycles. DOXIL may be administered through a central or peripheral venous catheter.

Treatment Arms A and B:

For both treatment arms, a scheduled dosing window of +2 days will be allowed for administrative purposes (eg, clinic scheduling, national holiday, patient's daughter is getting married). Administration of study drug within this window will not be considered a dosing delay and should not be recorded as such on the case report form (CRF) unless the delay is to allow for recovery from an adverse event. Detailed information on the requirements for storage, reconstitution and dilution, accountability, and disposal of trabectedin can be found in the Investigator Brochure.

Treatment Options – Amendment 6:

For subjects assigned to Arm A (trabectedin+DOXIL), treatment with trabectedin should be immediately discontinued. All study subjects (Arm A or Arm B) currently on study who, in the opinion of the investigator, are deriving clinical benefit may continue treatment with single-agent DOXIL as per the local standard of care. Treatment for these subjects may continue as long as the subjects comply with protocol-specified prohibitions and restrictions for treatment (Section 4.3) and as long as they experience clinical benefit in the opinion of the investigator. DOXIL will be provided by the JR&D as needed until post-trial access to DOXIL is available or until disease progression, whichever occurs first.

6.1. Criteria for Continuation of Treatment

On Day 1 of each subsequent treatment cycle (Cycle 2 and beyond), the criteria specified in Table 1 must be met in order for treatment to occur. If the criteria in Table 1 are not met on Day 1 of a new cycle, the subject must be evaluated at least weekly and the new cycle will start upon recovery to the Day 1 criteria specified in Table 1. Dosing may be delayed for a maximum of 3 weeks to allow for recovery of toxicity. If toxicity does not resolve after 3 weeks of delay, the subject must discontinue treatment. If it is determined that a subject can continue treatment, it may be necessary to reduce the dose of study drug based on maximum toxicity or critical analytes or toxicities, that occurred since the previous dose as described in Section 6.2, Dose Modification.

Table 1: Criteria for Continuation of Treatment

Variable	Day 1
Platelets	≥100,000/µL
ANC	≥1,500/µL
Hemoglobin	≥9 g/dL
Bilirubin	≤ ULN
ALP	≤2.5 x ULN; unless ALP liver fraction or 5' nucleotidase is normal[a]
Albumin	≥25 g/L
Transaminases	≤2.5 x ULN
Creatine phosphokinase	≤2.5 x ULN
Serum creatinine	≤1.5 mg/dL
LVEF[b]	≥LLN
Other nonhematologic, drug-related effects	Grade 2 or lower

ALP=alkaline phosphatase; ANC=absolute neutrophil count; LVEF=left ventricular ejection fraction; LLN=institutional lower limit of normal; ULN=upper limit of normal

[a] If the results of the ALP liver fraction or 5' nucleotidase are not yet available because of the length of time required to obtain these results, the subject may receive the study drugs to avoid a treatment delay only if on an earlier assessment, total ALP had a similar value and the ALP liver fraction or 5' nucleotidase was ≤ULN, confirming that the ALP elevation was of osseous origin. ALP liver fraction should be reported and evaluated in absolute units of measure.

[b] As determined by the most recent LVEF assessment.

Treatment should be discontinued for subjects who experience liver abnormalities that include all of the following within the same cycle:

- bilirubin ≥2 x ULN,
- transaminases (ALT or AST) ≥3 x ULN, and
- ALP (liver fraction) <2 x ULN (as reported in absolute units of measure).

Subjects must discontinue DOXIL for clinical evidence of cardiac toxicity or if the LVEF is compromised (ie, any absolute decrease in LVEF ≥10% and an LVEF value below the institutional lower limit of normal [LLN]). However, dosing with trabectedin should continue if the criteria for continuation of treatment are met (Table 1) and at the same dose. For those subjects on treatment Arm A who continue with trabectedin as a monotherapy after a cumulative anthracycline dose of 300 mg/m^2 has been exceeded or due to cardiotoxicity, regular monitoring of LVEF every 2 cycles of treatment should continue, regardless of the cumulative dose of anthracycline. All subjects in Arm A should undergo LVEF assessments after Cycle 3 of study treatment, even if DOXIL has been discontinued.

6.2. Dose Modification

Dose reductions will be made on the basis of the worst drug-related toxicity. However, investigators may also reduce the dose of either or both study drugs for toxicity, as clinically warranted. Dose reductions will be made on the basis of the worst drug-related toxicity that occurred since the previous dose, except for reductions due to AST or ALT increases that should only be based on Day 15 toxicity; see Section 6.2.1, Dose Reductions Due to Hematologic

Toxicity, and Section 6.2.2, Dose Reductions Due to Nonhematologic Toxicity. Table 2 and Table 3 specify the dose levels that will be used when dose reductions are required for subjects in the trabectedin+DOXIL combination therapy group or DOXIL monotherapy group, respectively. Subjects who experience toxicities that require more than 2 dose reductions of the same drug must discontinue treatment with that drug. If trabectedin is discontinued for a subject in Treatment Arm A, premedication with dexamethasone is no longer required. Dose escalations are not allowed following a dose reduction for toxicity.

Table 2: Dose Level Reductions – Trabectedin + DOXIL Group

Dose Level	Trabectedin	DOXIL
Starting level	1.1 mg/m^2	30 mg/m^2
Level –1	0.9 mg/m^2	25 mg/m^2
Level –2	0.75 mg/m^2	20 mg.m^2

Table 3: Dose Level Reductions – DOXIL Group

Dose Level	DOXIL
Starting level	50 mg/m^2
Level –1	37.5 mg/m^2
Level –2	28 mg/m^2

6.2.1. Dose Reductions Due to Hematologic Toxicity

Subjects in either treatment group who experience hematologic toxicity meeting either criteria cited in Table 4 will have a dose level reduction as specified in Table 2 (trabectedin+DOXIL group) or Table 3 (DOXIL group). Investigators may also reduce the dose of either or both study drugs for any toxicity, as clinically warranted. Only 2 dose reductions will be allowed. Subjects who experience toxicities that require more than 2 dose reductions must discontinue study treatment.

Table 4: Dose Reductions for Hematologic Toxicity

Nadir Toxicity	Nadir Value	Trabectedin	DOXIL
Neutrophil count	<500/µL with fever (≥38.5°C)/infection	Decrease 1 level	Decrease 1 level
	<500/µL lasting more than 5 days	Decrease 1 level	Decrease 1 level
Platelet count	<25,000/µL	Decrease 1 level	Decrease 1 level

The prophylactic use of G-CSF is strongly recommended for those subjects randomized to treatment Arm A (Section 8, Prestudy and Concomitant Therapy).[3]

6.2.2. Dose Reductions Due to Nonhematologic Toxicity

Subjects in either treatment group who experience non-hematologic toxicity meeting any of the criteria cited in Table 5 will have a dose level reduction as specified in Table 2 (trabectedin+DOXIL group) or Table 3 (DOXIL group). Investigators may also reduce the dose of either or both study drugs for any toxicity, as clinically warranted. Only 2 dose reductions of each study drug will be allowed. Subjects who experience toxicities that require more than 2 dose reductions of the same drug must discontinue study treatment with that drug.

Table 5: Dose Reductions for Nonhematologic Toxicity

Toxicity[a]	Worst CTC Grade	Trabectedin	DOXIL
**Nausea or vomiting despite adequate treatment**	≥3	Decrease 1 level	Decrease 1 level
**Stomatitis**	≥3	No reduction	Decrease 1 level
**Hand and foot syndrome (HFS)**			
• First occurrence	≥3	No reduction	Decrease 1 level
• Any occurrence after first occurrence of Grade 3/4 HFS	≥1	No reduction	Decrease 1 level
**Bilirubin >ULN at any time**	≥1	Decrease 1 level	No reduction
**Transaminase elevation (Day 15 of each cycle)**			
• Recovery to ≤Grade 1 by Day 1 of the next cycle or up to 3 weeks after this date	≥3[b]	Decrease 1 level	Decrease 1 level
• No recovery to ≤Grade 1 by Day 1 of the next cycle or up to 3 weeks after this date	≥3[b]	Treatment termination	Treatment termination
**Alkaline phosphatase (ALP)**	≥2[c]	Decrease 1 level	No reduction
**Creatine phosphokinase**	≥3	Decrease 1 level	No reduction
**Liver abnormalities within the same treatment cycle**			
• Bilirubin ≥2 x ULN, transaminases (ALT or AST) ≥3 x ULN, and ALP <2 x ULN		Treatment termination	Treatment termination
**LVEF decline ≥10% and <LLN or clinical evidence of cardiomyopathy**			
• First occurrence		No change	Treatment termination
• Subsequent occurrence		Decrease 1 level	Not applicable

ALP=alkaline phosphatase; ALT=alanine aminotransferase; AST=aspartate aminotransferase; CTC=common terminology criteria; HFS=hand and foot syndrome; LLN=institutional lower limit of normal; ULN=upper limit of normal

[a] Dose reductions for any other severe toxicity not listed in this table should be discussed with the sponsor.

[b] Worst CTC grade refers to measurements on Day 15 only.

[c] Unless non-hepatic source can be documented by ALP liver fraction ≤ULN or by 5'-nucleotidase ≤ULN.

For subjects in Treatment Arm A who are required to discontinue treatment with either trabectedin or DOXIL, treatment with the other agent may continue if the subject is benefiting from therapy. Subjects in Treatment Arm A who receive 2 dose reductions of trabectedin because of toxicity or can no longer receive dexamethasone or use a central line, may receive DOXIL as a single agent. For subjects in Treatment Arm A who receive 2 dose reductions of DOXIL or can no longer receive DOXIL because of cardiac or other toxicity, may receive trabectedin as a single agent. If the dose has not been previously reduced for related toxicity, the dose of the continued single-agent drug may be increased as monotherapy to 1.3 mg/m^2 for trabectedin every 3 weeks or 35 mg/m^2 for DOXIL every 3 weeks. The new dose will be subject to the same rules for adjustment as described above in this section. If DOXIL is discontinued due to cardiac toxicity, then refer to Table 5 for treatment guidelines for trabectedin monotherapy.

Any significant decrease in LVEF (defined as absolute decrease ≥15%, or less than LLN and absolute decrease ≥5%) should be captured as an adverse event regardless of assessed clinical significance or action taken. The supplemental cardiac adverse events form should also be completed for these adverse events.

7. TREATMENT COMPLIANCE

Study drugs will be administered as IV infusions by qualified staff at the study sites and the details of each administration will be recorded in the CRF (including date, start and stop times of the IV infusion, and volume infused). Treatment compliance (administration of the correct dose

according to the assigned schedule) will be documented in the hospital charts or notes and assessed on the basis of the completed treatment page of the CRF.

8. PRESTUDY AND CONCOMITANT THERAPY

Prestudy therapies administered up to 30 days before first dose of study drug must be recorded at screening.

Concomitant therapies must be recorded throughout the study beginning from the time the ICF is signed to 30 days after the last dose of study drug or until 18 January 2018, whichever occurs first. Concomitant therapies should be recorded beyond 30 days after the last dose of study drug only in conjunction with serious adverse events that meet the criteria outlined in Section 12.3.2, Serious Adverse Events.

All therapies (prescription or over-the-counter medications, including vaccines, vitamins, herbal supplements; non-pharmacologic therapies such as electrical stimulation, acupuncture, special diets, exercise regimens) different from the study drug must be recorded in the CRF. Recorded information will include a description of the type of the drug, treatment period, dosing regimen, route of administration, and its indication. Modification of an effective preexisting therapy should not be made for the explicit purpose of entering a subject into the study.

Premedication with IV dexamethasone (or an equivalent IV corticosteroid) is required for subjects in the trabectedin group. The use of IV dexamethasone (or an equivalent IV corticosteroid) for this purpose is not considered concomitant therapy and will be documented as a required pretreatment medication on the study drug administration section of the CRF. Additional corticosteroid administration is allowed for antiemetic purposes. The use of IV dexamethasone (or an equivalent IV corticosteroid) for reasons other than the required pretreatment of subjects in the trabectedin group should be documented in the concomitant therapy section of the CRF, and the associated indication should be recorded as an adverse event, if appropriate.

Any new condition or a worsening of an existing condition that requires the use of new or altered doses of concomitant therapies must be documented on the adverse events section of the CRF. Subjects may receive supportive care while receiving study drug, including transfusions, hematopoietic growth factors, antibiotics, analgesics, and antidiarrheal agents. Megestrol acetate may be used only for appetite stimulation.

Concomitant medications or therapies should be documented in the CRF. This includes:

- Antiinfective medications (eg, antibiotics, antifungals, antivirals)
- Hematopoietic cytokines
- Any medications the subject was receiving when a serious adverse event started and medications administered to treat the serious adverse event.
- Blood product transfusions received (the events that lead to the need for blood product transfusion should be recorded as adverse events, but not the transfusions themselves).

- Analgesics, antiemetics, antidiarrheal agents
- All inhibitors or inducers of cytochrome CYP3A4 (see below)

The metabolism of trabectedin may be modified by concomitant administration of compounds that induce or inhibit CYP3A4. Caution should be exercised if administration of such agents becomes necessary. Inhibitors of CYP3A4 include but are not limited to the following: aprepitant, clarithromycin, clotrimazole, diltiazem, erythromycin, fluconazole, grapefruit juice, indinavir, itraconazole, ketoconazole, nefazodone, nelfinavir, ritonavir, saquinavir, telithromycin, troleandomycin, and verapamil. Inducers of CYP3A4 include but are not limited to the following: barbiturates, phenytoin, carbamazepine, rifabutin, rifampin, and St. John's wort.

Supportive Care Measures

- Prophylactic use of G-CSF is strongly recommended for subjects assigned to the combination arm (Arm A).
- Subjects may be enrolled into the study while receiving recombinant erythropoietin provided they have received recombinant erythropoietin for at least 4 weeks before the first dose of study drug.
- Subjects may continue receiving hormone replacement therapy while on study.
- Subjects may receive pyridoxine (vitamin B6) at 200 mg by mouth daily to help prevent HFS due to DOXIL. If given, treatment should begin before Day 1, Cycle 1.
- All subjects receiving DOXIL should be cautioned to avoid vigorous activity, pressure on the skin, and exposure to extreme heat, hot water, and sun to reduce HFS-related symptoms.

For any concomitant therapy administered as a treatment for a new condition or a worsening of an existing condition, the condition must be documented on the adverse events page in the CRF.

9. STUDY EVALUATIONS

Following Amendment 6, study data collection will cease when all subjects on study treatment have completed the treatment termination visit assessments as specified in the Time and Events Schedule for Amendment 6 or by 18 January 2018, whichever occurs first. For subjects continuing treatment with single-agent DOXIL, as per the local standard of care, only serious adverse events should be reported to JR&D as outlined in Section 12.3.2.

9.1. Study Procedures

9.1.1. Overview

The Time and Events Schedule for Amendment 6 summarizes the treatment termination visit assessments.

All visit-specific PRO assessments during a visit should be conducted before any tests, procedures, or other consultations for that visit to prevent influencing subject perceptions.

Additional serum or urine pregnancy tests may be performed, as determined necessary by the investigator or required by local regulation, to establish the absence of pregnancy at any time during the subject's participation in the study.

The blood volumes collected from each subject for laboratory evaluations throughout this study are approximately 15 mL for screening serum chemistry and hematology tests; 10 mL for BRCA 1/2 testing at screening (for subjects who do not have a known BRCA 1/2 status); 4 mL for subjects consenting to the optional pharmacogenomics component of the study (where local regulations permit); 45 mL and 60 mL for treatment Arms A and B, respectively, per cycle for serum biochemistry and hematology tests; 4 mL per scheduled CA-125 sample; and 24 mL from those subjects who provide PK samples. Repeat or unscheduled samples may be taken for safety reasons.

9.1.2. Screening Phase

Informed consent must be obtained before the conduct of any protocol-specific procedure. All screening procedures must be completed and results obtained, where applicable, before random assignment to treatment. It should be noted, however, that clinical assessments performed as part of the subject's routine clinical evaluation and not specifically for this study need not be repeated after signed informed consent has been obtained, provided that these assessments fulfill the study's requirements.

The Screening Phase begins with the signing of the informed consent and should not last longer than 30 days. During the Screening Phase, the medical monitors will confirm subject eligibility based on previous therapy history, according to inclusion criteria #4 through #8 [Section 4.1, Inclusion Criteria]). Response to second-line platinum-based therapy will be confirmed either based on imaging demonstrating at least a 30% decline in tumor burden or a 50% reduction of elevated baseline serum CA-125 levels (≥2 x ULN, measured within approximately 4 weeks prior to the first dose of second-line platinum-based therapy that is maintained after at least 28 days as modified from Gynecologic Cancer Intergroup [GCIG] criteria [Attachment 1]).

A complete medical history and review of systems will be taken, including concomitant medications, prior therapy, and a description of key features of the disease including disease histology. Each subject must have an ECG and LVEF assessment (MUGA scan or 2D-ECHO) to document the LVEF is within normal range according to the institutional guidelines. A physical examination (including vital signs, weight, and height) will be performed, and BSA will be calculated either during screening or on Day 1 of Cycle 1. Laboratory evaluations (CA-125 measurement at a local laboratory, hematology and serum chemistry) will be done, and a urine or serum pregnancy test will be done for women of childbearing potential. Eastern Cooperative Oncology Group performance status will be assessed. BRCA 1/2 status will be collected on the CRF for subjects who have previously been tested for BRCA 1/2 mutations. For subjects who do not have a known BRCA 1/2 status, a blood sample will be collected to determine BRCA 1/2 status during the screening phase. It is expected that BRCA 1/2 analysis will require approximately 10 to 14 days. In the case of delayed BRCA 1/2 mutation reporting, the screening period may be extended for up to 14 days (with medical monitor approval). In such cases, only

screening clinical laboratory assessments (hematology, clinical chemistry, and liver panel) will need to be repeated. On Day 1 of Cycle 1, subjects will also be offered the option of providing a whole blood sample for pharmacogenomics analysis. Subject participation in the pharmacogenomic research component of the study is voluntary and refusal to participate will not result in ineligibility for the main part of the study. Consent for this is independent of the subject's consent for the main body of the study. Either consent or refusal to participate in the pharmacogenomic analysis must be documented within the pharmacogenomics consent form.

Screening procedures to be completed within 30 days before randomization include:

- Signed informed consent (including the optional pharmacogenomics informed consent, where applicable).
- Review of eligibility criteria
- Review of medical history and demographics
- Review of prestudy therapy
- Review of preplanned surgery/procedures(s)
- LVEF assessment (MUGA or 2D-ECHO) and ECG
- Tumor assessments, including radiographic imaging of the chest (with lung views), abdomen, and pelvis.
- BRCA 1/2 status (In case of delayed BRCA 1/2 mutation reporting, the screening period may be extended for up to 14 days, with medical monitor approval.)
- Pregnancy testing
- Physical examination
- Vital signs
- ECOG performance status
- Laboratory tests including CA-125, hematology, and clinical chemistry
- Review of concomitant therapy
- Review of adverse events
- Submission of redacted source documents supporting subject eligibility relevant to inclusion criteria #4 through #8.
- On or prior to Day 1 of Cycle 1, all subjects will be provided with a "wallet (study) card" and instructed to carry this card with them for the duration of the study.

9.1.3. Open-Label Treatment Phase

Following Amendment 6, study data collection including laboratory tests, cardiovascular monitoring, physical examinations, tumor assessments, PROs, and ECOG status will cease when all subjects on study treatment have completed the treatment termination visit assessments as specified in the Time and Events Schedule for Amendment 6 or by 18 January 2018, whichever occurs first. For subjects continuing treatment with single-agent DOXIL, as per the local

standard of care, only serious adverse events should be reported to JR&D as outlined in Section 12.3.2.

9.1.3.1. Clinical Laboratory Tests

Blood samples for serum chemistry and hematology may be obtained from a central venous catheter or a peripheral vein. Blood samples will be obtained on Days 1, 8, and 15 for subjects assigned to Arm A and on Days 1, 8, 15, and 22 for subjects assigned to Arm B. Day 1 blood samples should be obtained before administration of the study drugs. For both treatment arms, CA-125 samples will be collected at screening, approximately every 8 weeks after randomization for the first 4 evaluations, then approximately every 12 weeks until disease progression is radiographically confirmed, and at the treatment termination visit. For subjects who terminate treatment prior to disease progression, CA-125 samples will be collected during the follow-up phase according to schedule until disease progression is radiographically confirmed, the start of subsequent anticancer therapy, withdrawal of subject consent, or the clinical cutoff date. All CA-125 sample collections should coincide (±5 days) with the scheduled tumor assessments. All blood samples are to be obtained within 2 days prior to drug administration on Day 1 and ±2 days on Days 8 and 15 (Treatment Arm A) or Days 8, 15, and 22 (Treatment Arm B) of each treatment cycle. If a subject has an ANC of <500/µL, the ANC for this subject should be repeated, at most 5 days later to document recovery to >500/µL, and the scheduled visit can occur up to 3 days earlier in order to obtain the blood sample to perform this assessment.

Laboratory reports will be maintained at the study sites as source documents. Any clinically significant changes occurring during the study must be recorded on the adverse event page in the CRF.

Serum Chemistry

A sample of approximately 10 mL of venous blood will be collected for the following assessments: total protein, ALP, AST, ALT, CPK, total (direct and indirect) bilirubin, blood urea nitrogen (BUN), creatinine, sodium, potassium, glucose, and albumin.

Hematology

A sample of approximately 5 mL of venous blood will be collected for the following assessments: hemoglobin, ANC, white blood cell count (WBC), and platelet count.

9.1.3.2. Cardiovascular Safety

Left ventricular ejection fraction assessments (MUGA scans or 2D-ECHOs) will be performed for all subjects at screening (baseline), after an approximately 100 mg/m^2 cumulative dose of planned anthracycline treatment with DOXIL (ie, after Cycle 3 for treatment Arm A and after Cycle 2 for treatment Arm B) and as part of end-of-treatment assessments (ie, end-of-treatment termination visit). In addition, those subjects who receive a total cumulative dose of anthracycline exceeding 300 mg/m^2 (including previous and on-study treatments) or have a clinically significant history of cardiomyopathy should have follow-up LVEF assessments after every 2 cycles of therapy, or as clinically warranted. The same methodology (MUGA scans or

2D-ECHOs) should be used to assess scheduled LVEF assessments throughout the study. Additional LVEF assessments performed during the study should be reported as unscheduled LVEF assessments.

9.1.3.3. Physical Examination

A physical exam is to be performed and the results recorded only once during screening.

9.1.3.4. Tumor Assessment

Measurable disease and the response criteria used in this protocol are defined in the RECIST guidelines (Version 1.1) (Attachment 2) and will be based on radiologic assessment only.

Every effort should be made to ensure that the same radiographic method of disease assessment (computed tomography [CT] scans or magnetic resonance imaging [MRI]) be used throughout the study. Disease assessment procedures to evaluate objective response must be consistent with RECIST (Version 1.1) guidelines for radiologic assessment, as presented in Attachment 2, RECIST Guidelines. The disease assessments should include, at a minimum, imaging of the chest (with lung views) abdomen, and pelvis. All disease assessments, including CA-125 measurements, will be performed within 30 days before randomization, every 8 weeks (±5 days) from the date of randomization for the first 4 assessments, and then every 12 weeks (±5 days) until disease progression is radiographically confirmed, the start of subsequent anticancer therapy, withdrawal of subject consent, or the clinical cutoff date. Disease assessments should be performed at the treatment termination visit and according to schedule thereafter if disease progression or follow-up therapy has not been documented. Every effort should be made to ensure that these assessments are done on the required date, although a window of ±5 days will be accommodated.

In case of clinical progression, radiologic assessments should proceed according to the original Time and Events Schedule or as clinically indicated. The date of progression will be defined as the date of the first imaging study that documents progression.

Elevated CA-125 in the absence of radiographic progression is not to be considered evidence of disease progression. Therefore, it should not trigger an unscheduled assessment of tumor status nor result in treatment discontinuation. Other signs or symptoms deemed related to disease when suggestive of progression, including escalating pain not referable to another cause, increased ascites, protracted nausea and vomiting despite treatment, declining performance status, and examination findings consistent with disease progression, are valid reasons to consider an unscheduled assessment of tumor status. In this case, the assessment would include both imaging and CA-125. To the extent possible, it is important that all subjects have radiologic documentation of progression before study withdrawal or the initiation of follow-up therapy.

If a subject requests discontinuation of the study treatment but consents to continued evaluation, follow-up according to study procedure should be performed. In particular, tumor status assessment should be done according to the study timetable.

9.1.3.5. Patient Reported Outcomes Assessments

Health-related quality of life will be assessed using the quality of life questionnaire (QLQ) OV28 (Attachment 3). An additional PRO instrument, EQ-5D-5L (Attachment 4), will also be administered to perform preference based utility analysis. These questionnaires will be collected before any other tests or procedures are conducted on Day 1 of each treatment cycle and at the end-of-treatment evaluations. In addition, the EQ-5D-5L questionnaire will be collected approximately every 8 weeks thereafter until start of subsequent anticancer therapy.

9.1.3.6. Other Assessments

Eastern Cooperative Oncology Group performance status will be assessed during screening and at treatment discontinuation. Adverse events and changes in concomitant medications should be recorded at every visit.

9.1.4. Posttreatment Phase (Follow-Up)

Termination of Treatment

As of Amendment 6, all subjects who are on study treatment should have treatment termination visit assessments as specified in the Time and Events Schedule for Amendment 6 (ie, within 30 days of the last dose of study drug) or by 18 January 2018, whichever occurs first.

For subjects who discontinue treatment for reasons other than radiographic disease progression, tumor assessments (including CA-125) will continue on schedule until: 1) disease progression is radiographically confirmed, 2) the subject withdraws consent, 3) the subject begins subsequent anticancer therapy, or 4) the study is terminated (ie, clinical cutoff). For subjects who discontinue study treatment, documentation of all subsequent anticancer therapy, survival status, and safety evaluations as outlined in the Time and Events Schedule will be required. Survival status should be recorded at least every 8 weeks for the first 2 years after the treatment termination visit and approximately every 12 weeks thereafter. Collection of survival status will continue until at least 514 deaths have been observed or until the clinical cutoff date. The access to study treatment will remain available for subjects who are consenting and eligible for treatment continuation. Once the study has been terminated, reporting will be limited to unexpected, drug-related serious adverse events during treatment or within 30 days of last dose of the study treatment.

Assessments at Treatment Termination Visit

When it has been determined that a subject should permanently discontinue study treatment, she will be asked to return for a treatment termination visit. This visit should occur 30 days (+3 days) after the last dose of study drug. At this visit, the following assessments will be recorded: ECOG performance status, ECG, MUGA scan or 2D-ECHO, assessment of tumor status including CA-125 measurement, PRO assessments, and hematology and clinical chemistry assessments. Adverse events and concomitant therapies until 30 days after the last dose of study drug will be recorded.

Follow-up Data Collection after Treatment Termination

As of Amendment 6, follow-up data will no longer be collected.

Follow-up assessments for survival status should occur at least every 8 weeks for the first 2 years after the treatment termination visit and then approximately every 12 weeks thereafter, until death or the clinical data cutoff date. Any clinically significant abnormalities persisting at 30 days after the last dose of study drug will be followed by the investigator until resolution or until a clinically stable endpoint is reached. Drug-related Grade 3 or Grade 4 toxicities will be monitored until Grade 2 or less, or for a maximum of 6 months after the last dose of study drug, whichever, occurs first. Grade 2 to 4 liver or cardiac toxicities will be monitored until Grade 1 or less, or for a maximum of 6 months after the last dose of study drug, whichever occurs first.

Disease assessments (including CA-125) will be performed using the same radiographic technique (CT scans or MRI) approximately every 8 weeks after randomization for the first 4 assessments and then approximately every 12 weeks until disease progression is radiographically confirmed, the start of subsequent anticancer therapy, withdrawal of subject consent, or the clinical cutoff date. Subjects who have disease progression at treatment termination should continue to be followed for survival and the start of subsequent anticancer therapy. Every effort should be made to record all subsequent anticancer therapies, along with their dates of administration.

9.2. Efficacy

9.2.1. Evaluations

Efficacy will be assessed by determination of the OS, PFS, and ORR end points (Attachment 2).

Appropriate radiological disease assessments (CT scans or MRI) of the chest (with lung views), abdomen, and pelvis and will be performed within 30 days before randomization, approximately every 8 weeks (±5 days) after randomization for the first 4 assessments, and then every 12 weeks (±5 days) until disease progression is radiographically confirmed, the start of subsequent anticancer therapy, withdrawal of subject consent, or the clinical cutoff date (18 January 2018). Assessments must be done consistently (every 8 weeks [±5 days] for the first 4 assessments and then every 12 weeks [±5 days]) in both treatment arms to ensure an unbiased assessment of tumor response and progression.

CA-125 analysis will be done at screening, approximately every 8 weeks after randomization for the first 4 evaluations and then approximately every 12 weeks until disease progression is radiographically confirmed, the start of subsequent anticancer therapy, withdrawal of subject consent, or the clinical cutoff date (18 January 2018). CA-125 sample collections should coincide (±5 days) with the scheduled tumor assessments. Analyses of CA-125 will be done at a local laboratory.

CA-125 shall not be used as a basis to assess disease response or progression during study treatment and should not guide treatment decisions.

Survival follow up will continue for all enrolled subjects until at least 514 deaths are observed or until the clinical cutoff date.

9.2.2. Endpoints

Primary Endpoint

- Overall survival (OS), is defined as the time between the date of randomization and the date of death.

Secondary Endpoints

- Progression-free survival (PFS), is defined as the time between the date of randomization and the date of disease progression or death;
- Objective response rate (ORR), defined as the total number of subjects with either a CR or PR
- Population PK using a sparse sampling scheme
- Safety will be described according to the frequency of adverse events that occur in the treatment arms.

Exploratory Endpoints

- Pharmacogenomic evaluation of BRCA 1 or BRCA 2 mutation status (mutation vs no mutation).
- Patient reported outcomes using the QLQ-OV28 and EQ-5D-5L questionnaires.

9.3. Pharmacokinetics

9.3.1. Evaluations

Sparse samples for measurement of trabectedin concentrations in plasma will be collected from at least 40 subjects in the trabectedin+DOXIL treatment group.

Venous blood samples of 4 mL each will be collected for determination of plasma concentrations of trabectedin within 1 h before the start of trabectedin infusion, at 1 h after start of infusion, at 10 minutes before the end of infusion, and 0.5 h, 24 h, and 168 h after the end of infusion on Cycle 1. The actual dates and times of sample collection must be recorded on the laboratory requisition form. Blood samples will be obtained through an indwelling peripheral venous catheter; therefore, an appropriate amount (1 mL) of serosanguineous fluid slightly greater than the dead space volume of the lock will be removed from the cannula and discarded before each blood sample is taken. Blood samples will be obtained directly from the lock in a slow manner into the Vacutainer tube. After blood sample collection, the cannula will be flushed with 0.9% sodium chloride, United States Pharmacopeia (USP) (or equivalent) and charged with a volume equal to the dead space volume of the lock. Instructions for blood collection, sample handling, and shipment are provided in the laboratory manual.

Genetic analyses will not be performed on these plasma samples, and subject confidentiality will be maintained.

9.3.2. Analytical Procedures

Pharmacokinetics

Plasma samples will be analyzed to determine concentrations of trabectedin using a validated, specific, and sensitive liquid chromatography/mass spectrometry/mass spectrometry (LC-MS/MS) method by or under the supervision of the sponsor.

9.3.3. Pharmacokinetic Parameters

Pharmacokinetic analysis will be the responsibility of the sponsor in accordance with the current Clinical Pharmacokinetics Guideline. Clearance and volume of distribution will be the primary parameters of interest for the population PK analysis. Additional PK parameters will be calculated, if deemed appropriate.

9.4. Pharmacogenomic Evaluations

9.4.1. Mandatory BRCA 1 and BRCA 2 Mutation Analysis

Reporting of BRCA 1/2 status will be required prior to randomization and is to be collected on the CRF. For subjects who do not have a known BRCA 1/2 status, a blood sample will be collected, and BRCA 1/2 status determined within 30 days prior to randomization. It is expected that BRCA 1/2 analysis will require approximately 10 to 14 days. In the case of delayed BRCA 1/2 mutation reporting, the screening period may be extended for up to 14 days (with medical monitor approval). In such cases, only screening clinical laboratory assessments (hematology, clinical chemistry, and liver panel) will need to be repeated. Because there are multiple possible mutations for any one subject, stratification will be based on the presence of a BRCA 1/2 mutation (mutation vs no mutation).

For subjects with a known mutation status for either BRCA 1 or BRCA 2, but not for both genes, the result from the single gene tested will be captured in the CRF. If the status of the reported gene indicates a mutation, the status will be used for randomization. However, if the single gene tested indicates no mutation, then BRCA testing for the other gene is to be conducted and reported within 30 days prior to randomization.

9.4.2. Optional Pharmacogenomic Analyses

Subjects will be given the opportunity to participate in the optional pharmacogenomic component of this study, where local regulations permit. Collection of pharmacogenomic samples will allow for genetic research to help understand trabectedin/DOXIL activity in advanced-relapsed epithelial ovarian, primary peritoneal, and fallopian tube cancers. Genetic analysis will be conducted if it is hypothesized that this may help resolve issues with the clinical data. DNA samples will be used for research related to trabectedin/DOXIL and advanced-relapsed epithelial ovarian, primary peritoneal, and fallopian tube cancers. They may also be used to develop tests/assays related to trabectedin/DOXIL and advanced-relapsed

epithelial ovarian, primary peritoneal, and fallopian tube cancers. Genetic research may consist of the analysis of one or more candidate genes or of the analysis of genetic markers throughout the genome (as appropriate) in relation to trabectedin/DOXIL and advanced-relapsed epithelial ovarian, primary peritoneal, and fallopian tube cancer clinical endpoints.

9.5. Safety Evaluations

Details regarding the IDMC are provided in Section 11.9, Independent Data Monitoring Committee.

Any clinically significant abnormalities persisting 30 days after the last dose of study drug will be followed by the investigator until resolution, until a clinically stable endpoint is reached, or until the clinical cutoff date (18 January 2018). Drug-related Grade 3 or Grade 4 toxicities will be assessed until Grade 2 or less, for a maximum of 6 months after the last dose of study drug, or until the clinical cutoff date (18 January 2018), whichever occurs first. Grade 2 to 4 liver or cardiac toxicities will be monitored until the toxicity is Grade 1 or less, for a maximum of 6 months after the last dose of study drug, or until the clinical cutoff date (18 January 2018), whichever occurs first.

In selected cases of cardiac adverse events, investigators may be asked to complete a supplemental CRF section in order to provide more detailed information relating to the event. Any additional LVEF assessments should be reported as unscheduled assessments.

Note: Following Amendment 6, study data collection for adverse events (except for serious adverse events), laboratory tests, cardiovascular monitoring, vital signs, and physical examinations will cease when all subjects on study treatment have completed the treatment termination visit assessments as specified in the Time and Events Schedule for Amendment 6 or by 18 January 2018, whichever occurs first. For subjects continuing treatment with single-agent DOXIL, as per the local standard of care, only serious adverse events should be reported to JR&D as outlined in Section 12.3.2.

Adverse Events

Adverse events will be reported by the subject or investigator (or, when appropriate, by a caregiver, surrogate, or the subject's legally-acceptable representative) for the duration of the study. Adverse events will be followed by the investigator as specified in Section 12, Adverse Event Reporting.

Clinical Laboratory Tests

Blood samples for serum chemistry and hematology will be collected. The investigator must review the laboratory report, document this review, and record any clinically relevant changes occurring during the study in the adverse event section of the CRF. The following tests will be performed by the local laboratory and entered into the CRF:

- Hematology Panel

  -hemoglobin
  -absolute neutrophil count (ANC)
  -white blood cell (WBC) count
  -platelet count

- Serum Chemistry Panel

  -sodium
  -potassium
  -blood urea nitrogen (BUN)
  -creatinine
  -glucose
  -aspartate aminotransferase (AST)
  -alanine aminotransferase (ALT)
  -total (direct and indirect) bilirubin
  -alkaline phosphatase (ALP)
  -creatine phosphokinase (CPK)
  -albumin
  -total protein

- Serum or urine pregnancy testing for women of childbearing potential only.

Potential cases of hepatotoxicity will be thoroughly investigated. Investigators will complete a supplemental CRF section and provide additional supporting documentation for all subjects who experience all of the following within the same cycle: total bilirubin values ≥2 x ULN, elevated transaminase values (AST or ALT) ≥3 x ULN, and ALP values ≤2 x ULN.

Electrocardiogram and Left Ventricular Ejection Fraction Assessments

The study will include evaluations of ECGs and LVEF. The same procedure for the assessment of LVEF (MUGA scan or 2D-ECHO) should be used for all scheduled assessments throughout the study. The screening (baseline) LVEF assessment and ECG must be performed within 30 days before randomization, and the LVEF must be within normal range according to the institutional guidelines. Left ventricular ejection fraction assessments will be performed after Cycle 3 for treatment Arm A and after Cycle 2 for treatment Arm B (ie, within the 21 day period after Cycle 3 Day 1 and the next scheduled dose and within the 28 day period after Cycle 2 Day 1 and the next scheduled dose, respectively). In addition, subjects' who have received a cumulative dose of anthracyclines that exceeds 300 mg/m^2 (including previous and on-study treatments) or have a clinically significant history of cardiomyopathy, must have follow-up LVEF assessments after every 2 cycles of therapy. Each subject must also have a LVEF assessment at the treatment termination visit. Subsequent LVEF assessments may be indicated during the follow-up period if significant LVEF decline is noted (Section 9.1.4) at the time of end of treatment.

During the collection of ECGs and LVEF assessments, subjects should be in a quiet setting without distractions (eg, television, cell phones). Subjects should rest in a supine position for at least 5 minutes before the ECG and LVEF assessments and should refrain from talking or moving arms or legs. If blood sampling or vital sign measurement is scheduled for the same time point as ECG recording, the procedures should be performed in the following order: ECGs, LVEF assessments, vital signs, blood draw.

Vital Signs (temperature, pulse/heart rate, respiration rate, and blood pressure)

Blood pressure and heart rate measurements will be assessed while the subject is in a seated position with a completely automated device. Manual techniques will be used only if an automated device is not available.

Blood pressure and heart rate measurements should be preceded by at least 5 minutes of rest in a quiet setting without distractions (eg, television, cell phones).

Physical Examination (including height and weight measurements)

9.6. Sample Collection and Handling

The actual dates and times of sample collection must be recorded in the CRF or laboratory requisition form. For the pharmacokinetic samples, the actual dates and times of sample collection must be recorded on the laboratory requisition form. If blood samples are collected via an indwelling cannula, an appropriate amount (1 mL) of serosanguineous fluid slightly greater than the dead space volume of the lock will be removed from the cannula and discarded before each blood sample is taken. After blood sample collection, the cannula will be flushed with 0.9% sodium chloride, USP (or equivalent) and charged with a volume equal to the dead space volume of the lock. Refer to the Time and Events Schedule for the timing and frequency of all sample collections.

Instructions for the collection, handling, and shipment of samples are found in the laboratory manual that will be provided. Collection, handling, storage, and shipment of samples must be under the specified, and where applicable, controlled temperature conditions as indicated in the laboratory manual.

10. SUBJECT COMPLETION/WITHDRAWAL

10.1. Completion

A subject will be considered to have completed the study if she has experienced a clinical endpoint that precludes further study (eg, mortality in a mortality endpoint study) or has withdrawn consent for follow-up. Following the completion of the treatment termination visit assessments on or by 18 January 2018, per protocol Amendment 6, data collection will end. For subjects continuing treatment with single-agent DOXIL, as per the local standard of care, only serious adverse events should be reported to JR&D as outlined in Section 12.3.2.

10.2. Discontinuation of Treatment

As of Amendment 6, treatment with trabectedin should be immediately discontinued for subjects assigned to Arm A (trabectedin+DOXIL). All study subjects (Arm A or Arm B) currently on study who, in the opinion of the investigator, are deriving clinical benefit may continue treatment with single-agent DOXIL as per the local standard of care. Treatment for these subjects may continue as long as the subjects comply with protocol-specified prohibitions and restrictions for treatment (Section 4.3) and as long as they experience clinical benefit in the opinion of the investigator. DOXIL will be provided by JR&D as needed until post-trial access to DOXIL is available or until disease progression, whichever occurs first.

If a subject's study treatment must be discontinued before disease progression has been documented, this will not result in automatic withdrawal of the subject from the study.

A subject's study treatment should be discontinued if:

- The subject has disease progression,
- The subject meets protocol prescribed criteria for drug discontinuation due to toxicity after appropriate dose reductions (Section 6.2),
- The subject does not meet the criteria for continuation of treatment (Section 6.1),
- The subject develops a concurrent medical condition (adverse event) that precludes further participation,
- The subject begins a subsequent anticancer therapy,
- The subject completes 2 cycles of treatment after assessment of a CR,
- The subject requests to stop receiving treatment, or
- The subject becomes pregnant.

If a subject discontinues study treatment before disease progression has occurred, end-of-treatment assessments should be obtained, and follow-up for scheduled assessments (eg, survival and radiographic disease assessments) should be continued.

10.3. Withdrawal From the Study

A subject will be withdrawn from the study for any of the following reasons:

- Lost to follow-up
- Withdrawal of consent (no further treatment and no further follow-up)

If a subject is lost to follow-up, every reasonable effort must be made by the study site personnel to contact the subject and determine the reason for discontinuation/withdrawal and survival status. The measures taken to follow up must be documented.

When a subject withdraws before completing the study, the reason for withdrawal is to be documented in the CRF and in the source document. Study drug assigned to the withdrawn subject may not be assigned to another subject. Subjects who withdraw will not be replaced.

A subject who withdraws from the study will have the following options regarding the optional pharmacogenomic research sample:

- The collected sample will be retained and used in accordance with the subject's original separate informed consent for optional research samples.
- The subject may withdraw consent for optional research samples, in which case the sample will be destroyed and no further testing will take place. To initiate the sample destruction process, the investigator must notify the sponsor site contact of withdrawal of consent for the optional research samples and to request sample destruction. The sponsor study site contact will, in turn, contact the biomarker representative to execute sample destruction. If requested, the investigator will receive written confirmation from the sponsor that the sample has been destroyed.

Withdrawal From the Optional Research Samples While Remaining in the Main Study

The subject may withdraw consent for optional research samples while remaining in the study. In such a case, the optional research sample will be destroyed. The sample destruction process will proceed as described above. However, all samples will be made nonidentifiable after the Clinical Study Report is issued and thereafter cannot be identified for destruction. If the sample has already undergone conversion to the nonidentifiable format, the sponsor will notify the investigator in writing.

11. STATISTICAL METHODS

Statistical analysis will be done by the sponsor or under the authority of the sponsor. A general description of the statistical methods to be used to analyze the efficacy and safety data is outlined below. Specific details will be provided in the Statistical Analysis Plan.

11.1. Subject Information

For all subjects who are randomly assigned to study drug descriptive statistics will be provided.

11.2. Sample Size Determination

This study is designed to demonstrate a statistically significant difference in OS between subjects treated with the trabectedin+DOXIL combination therapy and those treated with the DOXIL monotherapy in the third-line setting. The OS endpoint will incorporate group sequential design by including 1 interim analysis and 1 final analysis using the O'Brien-Fleming boundaries as implemented by Lan-DeMets α-spending method. It is assumed that failure will follow an exponential distribution with a constant hazard rate. Assuming a median OS of 16 months for the active control group (DOXIL monotherapy), a planned sample size of approximately 670 subjects will provide 80% power to detect a HR of 0.78 (16 months vs 20.5 months) at a 2-tailed significance level of 0.05 and an enrollment duration of approximately 52 months

(13 subjects/month enrollment) over a total study duration of 64 months to obtain the required 514 events.

11.3. Efficacy Analyses

All efficacy endpoints will be analyzed using the All Randomized set of the population. The Kaplan-Meier product limit method and un-stratified Cox proportional hazards model will be used to estimate the time-to-event variables and to obtain the HR and their 95% CI. The un-stratified log-rank test will be used as the primary analysis for treatment comparison. The response rate variables will be evaluated using Chi-square statistic (un-stratified).

Sensitivity analyses for the primary endpoint using the stratified log-rank test will also be performed. Subgroup analyses will be carried out to assess if the treatment effect is consistent across clinically relevant subgroups.

Primary Endpoint

Overall survival is defined as the time between the date of randomization and the date of death. Subjects who die, regardless of the cause of death, will be considered to have had an event. Subjects who are still being treated, who are lost to follow-up prior to the end of the study, or who have withdrawn consent from the study will be censored at the last available date where the subject is known to be alive.

Overall survival will be compared between both treatment arms by the un-stratified, 2-sided, log-rank test. The analysis of the primary endpoint, OS, will be conducted after at least 514 events (deaths) are observed or until the clinical cutoff date. An interim analysis is planned for this study; after observing 60% (308 death events) of the total number of required (514) death events. The Kaplan-Meier method will be used to estimate the distribution of functions of OS for each treatment arm. The number of events, subjects censored, the estimate of medians, and 95% CI for the medians will be presented. Survival rates will be calculated using the Kaplan-Meier method. The un-stratified Cox proportional hazards model will be used to obtain the HR and its 95% CI.

Overall survival will also be compared between the treatment arms by a 2-sided log-rank test, stratified for: the time from the last dose of first-line platinum therapy to disease progression (6 months to 12 months vs >12 months to 24 months vs >24 months), ECOG performance status grade (0 vs 1), BRCA 1/2 status (mutation vs no mutation), and prior DOXIL therapy (no vs yes).

In case an imbalance in baseline prognostic factors is observed, especially PFI, a Cox proportional hazards model will also be used to compare the 2 treatment arms. The following baseline information includes as covariates: baseline ECOG (0 vs 1), PFI (as continuous), BRCA 1/2 status (mutation vs no mutation), prior DOXIL therapy (no vs yes), and any imbalanced factors. From the Cox proportional hazards regression, HR estimates and their 95% CIs will be estimated for treatment and for the prognostic factors.

Secondary Endpoints

Progression-free survival is defined as the time between the date of randomization and the date of disease progression or death. Subjects who progressed or died will be considered to have had an event, except if the event occurs after the start of subsequent therapy for ovarian cancer, in which case the subject will be censored at the time of the last tumor assessment (prior to or on the first day of the first subsequent therapy for ovarian cancer). Subjects who do not progress or die (ie, lost to follow-up, or receiving treatment without documented disease progression, or started subsequent therapy for ovarian cancer and still alive) will be censored at the date of the last tumor assessment (prior to or on the first day of the first subsequent therapy for ovarian cancer).

Progression-free survival will be compared between both treatment arms using the un-stratified log-rank test. The Kaplan-Meier method will be used to estimate the distribution function of PFS for each treatment arm. The number of events, subjects censored, the estimate of medians, and 95% CIs for the medians will be presented. Six-month and 1-year progression-free rates will be calculated using the Kaplan-Meier product limit method.

Objective response rate is defined as the total number of subjects with either a CR or PR as defined by RECIST (Version 1.1). All other subjects will be considered non-responders in the analysis. The response rate will be evaluated using the Chi-square statistic (un-stratified).

The ECOG value at the end of treatment visit will be tabulated (not cross-tabulated against the baseline value). The listing will contain both ECOG values.

Hierarchical Testing

In the event that the primary endpoint of OS is significant, the Hochberg procedure will be used to hierarchically test the secondary endpoints at the overall (2-sided) significance level 0.05.

11.4. Pharmacokinetic Analyses

Sparse PK data will be listed in the population PK-report, for all subjects with available concentrations in the trabectedin+DOXIL treatment group. Subjects will be excluded from the PK analysis if their data do not allow for accurate assessment of the PK (eg, improper handling of PK samples, incomplete administration of the study agent; missing time or dosing information). All concentrations below the lowest quantifiable concentration or missing data will be labeled as such in the concentration data presentation. All subjects and samples excluded from the analysis will be retained in the dataset but will be flagged out and the criteria for exclusion documented.

Population PK analysis of plasma concentration-time data of trabectedin will be performed using nonlinear mixed-effects modeling. Data may be combined with those of a selection of Phase 1, 2, and 3 studies to support a relevant structural model. Available subject characteristics (demographics, laboratory variables, genotypes, concurrent dexamethasone use, etc.) will be tested as potential covariates affecting PK parameters. Details will be given in a population PK analysis plan and the results of the population PK analysis will be presented in a separate report.

11.5. Patient Reported Outcomes

Patient reported outcome assessments based on the EQ-5D-5L and QLQ-OV28 questionnaires will be analyzed to determine if therapeutic response and/or side effects of therapy are accompanied by measurable changes in the PROs. The analysis will be performed on summary scores as well as subscales, and individual symptoms, with specific analytical methods outlined in a formal Statistical Analysis Plan developed prior to database lock.

Differences between the treatment arms will be evaluated using published methods. Specific interest centers on abdominal and gastrointestinal symptom scales of QLQ-OV28. The change in PRO scores between baseline and each postbaseline assessment will be described overall and according to the response to treatment.

11.6. Pharmacogenomic Analyses

BRCA 1/2 status will be evaluated for association with time-to-event endpoints (eg, OS and PFS) using the log-rank test. Response rate may also be evaluated using the appropriate categorical method. Analyses will be performed within and between each treatment group. The Kaplan-Meier method and Cox model will be used to obtain a HR and its 95% CI. Other clinical covariates, such as race, age, and tumor burden may also be included in the model.

11.7. Safety Analyses

All subjects who have received at least part of 1 dose of study drug will be included in the safety analysis, according to the actual treatment received. The safety parameters to be evaluated are the incidence, intensity, and type of adverse events, ECGs, LVEF assessments (MUGA scans or 2D-ECHOs), and clinical laboratory results. Exposure to the study drugs and reasons for discontinuation of study treatment will be tabulated.

Adverse Events

The verbatim terms used in the CRF by investigators to identify adverse events will be coded using the Medical Dictionary for Regulatory Activities (MedDRA) and will be graded according to the NCI-CTCAE, Version 4.0. All reported adverse events with onset during the treatment phase (ie, those events that occur or worsen on or after the first dose of study drug up through 30 days after the last dose of study drug) will be included in the analysis. Adverse events will be summarized by MedDRA system organ class and preferred term. For each adverse event, the percentage of subjects who experience at least 1 occurrence of the given event will be summarized overall and by treatment group. Serious adverse events and deaths will be listed. All adverse events resulting in discontinuation of study treatment, dose modification, the interruption of dosing, or a delay in treatment with the study drug will be listed and summarized by preferred term.

Clinical Laboratory Tests

Laboratory data will be summarized by type of laboratory test. The worst toxicity grade will be tabulated.

Parameters with predefined NCI-CTCAE, Version 4.0, toxicity grades will be summarized. Change from baseline to the worst adverse event grade experienced by the subject during the study will be provided as shift tables.

Electrocardiogram and Multigated Acquisition Scans or 2-Dimensional Echocardiograms

The effects on cardiovascular variables will be evaluated by means of descriptive statistics and frequency tabulations. A separate report on cardiac safety will also be prepared.

11.8. Interim Analysis

One interim analysis is planned for this study after observing 60% (308 events) of the total number of required (514) events (deaths). The α spent will be 0.008 for the interim analysis. The O'Brien-Fleming boundaries as implemented by Lan-DeMets α-spending functions are used for the boundary. The α spent will be adjusted accordingly based on the actual number of events observed. Operating characteristics for the boundaries are presented in Table 6.

Table 6: Statistical Operating Characteristics

	Interim (60% of Total Events)	Final
Projected Observed Events	308	514
Efficacy Boundary (HR)	0.74	0.84
Cumulative α spent	0.008	0.05

HR=Hazard ratio

11.9. Independent Data Monitoring Committee

An IDMC will be established to review the efficacy and safety data according to the prescribed statistical plan. The committee will meet periodically (approximately every 6 months) to review safety data. After the review, the IDMC will make recommendations regarding the continuation of the study. The details will be provided in a separate IDMC charter.

The committee will consist of members who are not employees of the sponsor and are not otherwise involved in the conduct of the study. The membership will include one or more statisticians and medical oncologists with experience in treating subjects with ovarian cancer.

Note: On 26 June 2017, the IDMC requested a futility analysis of OS to be performed at the time of the next meeting 6 months later. In view of the IDMC request, study data up to 20 September 2017 were evaluated for safety and OS (45% rather than 60% of the required death events at the planned interim analysis). Following the review of the study data by the IDMC on 15 December 2017, the HR for OS was 0.962, crossing the previously agreed upon threshold for futility of 0.93. In view of this result and the observed and expected higher toxicity in Arm A as compared with Arm B, the IDMC recommended discontinuing the study. Therefore, as of Amendment 6, no new subjects will be randomized to study treatment, and treatment with trabectedin should be immediately discontinued for subjects assigned to Arm A (trabectedin+DOXIL). All study subjects (Arm A or Arm B) currently on study who, in the opinion of the investigator, are deriving clinical benefit may continue treatment with single-agent

DOXIL as per the local standard of care. Treatment for these subjects may continue as long as the subjects comply with protocol-specified prohibitions and restrictions for treatment (Section 4.3) and as long as they experience clinical benefit in the opinion of the investigator. DOXIL will be provided by JR&D as needed until post-trial access to DOXIL is available or until disease progression, whichever occurs first.

12. ADVERSE EVENT REPORTING

Following Amendment 6, only serious adverse events for subjects continuing to receive single-agent DOXIL should be reported to JR&D as outlined in Section 12.3.2.

Timely, accurate, and complete reporting and analysis of safety information from clinical studies are crucial for the protection of subjects, investigators, and the sponsor, and are mandated by regulatory agencies worldwide. The sponsor has established Standard Operating Procedures in conformity with regulatory requirements worldwide to ensure appropriate reporting of safety information; all clinical studies conducted by the sponsor or its affiliates will be conducted in accordance with those procedures.

12.1. Definitions

12.1.1. Adverse Event Definitions and Classifications

Adverse Event

An adverse event is any untoward medical occurrence in a clinical study subject administered a medicinal (investigational or non-investigational) product. An adverse event does not necessarily have a causal relationship with the treatment. An adverse event can therefore be any unfavorable and unintended sign (including an abnormal finding), symptom, or disease temporally associated with the use of a medicinal (investigational or non-investigational) product, whether or not related to that medicinal (investigational or non-investigational) product. (Definition per International Conference for Harmonisation [ICH])

This includes any occurrence that is new in onset or aggravated in severity or frequency from the baseline condition, or abnormal results of diagnostic procedures, including laboratory test abnormalities.

Note: The sponsor collects adverse events starting with the signing of the ICF (refer to Section 12.3.1, All Adverse Events, for time of last adverse event recording).

Serious Adverse Event

A serious adverse event based on ICH and EU Guidelines on Pharmacovigilance for Medicinal Products for Human Use is any untoward medical occurrence that at any dose:

- Results in death
- Is life-threatening
  (The subject was at risk of death at the time of the event. It does not refer to an event that hypothetically might have caused death if it were more severe.)

- Requires inpatient hospitalization or prolongation of existing hospitalization
- Results in persistent or significant disability/incapacity
- Is a congenital anomaly/birth defect
- Is a suspected transmission of any infectious agent via a medicinal product
- Is Medically Important*

*Medical and scientific judgment should be exercised in deciding whether expedited reporting is also appropriate in other situations, such as important medical events that may not be immediately life threatening or result in death or hospitalization but may jeopardize the subject or may require intervention to prevent one of the other outcomes listed in the definition above. These should usually be considered serious.

Unlisted (Unexpected) Adverse Event/Reference Safety Information

An adverse event is considered unlisted if the nature or severity is not consistent with the applicable product reference safety information. For trabectedin, the expectedness of an adverse event will be determined by whether or not it is listed in the Investigator's Brochure. For DOXIL, the expectedness of an adverse event will be determined by whether or not it is listed in the package insert or the Committee for Medicinal Products for Human Use Summary of Product Characteristics.

Adverse Event Associated With the Use of the Drug

An adverse event is considered associated with the use of the drug if the attribution is possible, probable, or very likely by the definitions listed in Section 12.1.2, Attribution Definitions.

12.1.2. Attribution Definitions

Not Related

An adverse event that is not related to the use of the drug.

Doubtful

An adverse event for which an alternative explanation is more likely, eg, concomitant drug(s), concomitant disease(s), or the relationship in time suggests that a causal relationship is unlikely.

Possible

An adverse event that might be due to the use of the drug. An alternative explanation, eg, concomitant drug(s), concomitant disease(s), is inconclusive. The relationship in time is reasonable; therefore, the causal relationship cannot be excluded.

Probable

An adverse event that might be due to the use of the drug. The relationship in time is suggestive (eg, confirmed by dechallenge). An alternative explanation is less likely, eg, concomitant drug(s), concomitant disease(s).

Very Likely

An adverse event that is listed as a possible adverse reaction and cannot be reasonably explained by an alternative explanation, eg, concomitant drug(s), concomitant disease(s). The relationship in time is very suggestive (eg, it is confirmed by dechallenge and rechallenge).

12.2. Special Reporting Situations

Safety events of interest on a sponsor study drug that may require expedited reporting and/or safety evaluation include, but are not limited to:

- Overdose of a sponsor study drug
- Suspected abuse/misuse of a sponsor study drug
- Inadvertent or accidental exposure to a sponsor study drug
- Medication error involving a sponsor product (with or without subject/patient exposure to the sponsor study drug, eg, name confusion)

Up to and including 18 January 2018, special reporting situations should be recorded in the CRF. After 18 January 2018, any special reporting situation that meets the criteria of a serious adverse event should be reported to JR&D using the Serious Adverse Event Form (Section 12.3.2).

12.3. Procedures

12.3.1. All Adverse Events

All adverse events and special reporting situations, whether serious or non-serious, will be reported from the time a signed and dated ICF is obtained until 30 days after the administration of the last dose of study drug or until the scheduled treatment termination visit on or prior to 18 January 2018. Any drug-related Grade 3 or 4 toxicities will be followed by the investigator until Grade 2 or less, for a maximum of 6 months after the last dose of study drug, or until the scheduled treatment termination visit on or prior to 18 January 2018, whichever occurs first. Grade 2 to 4 liver or cardiac toxicities will be monitored until Grade 1 or less, for a maximum of 6 months after the last dose of study drug, or until the scheduled treatment termination visit on or prior to 18 January 2018, whichever occurs first.

Serious adverse events, including those spontaneously reported to the investigator within 30 days after the last dose of study drug, must be reported using the Serious Adverse Event Form. The sponsor will evaluate any safety information that is spontaneously reported by an investigator beyond the time frame specified in the protocol. All events that meet the definition of a serious adverse event will be reported as serious adverse events, regardless of whether they are protocol-specific assessments.

All adverse events, regardless of seriousness, severity, or presumed relationship to study drug, must be recorded using medical terminology in the source document and the CRF. Whenever possible, diagnoses should be given when signs and symptoms are due to a common etiology (eg, cough, runny nose, sneezing, sore throat, and head congestion should be reported as "upper respiratory infection"). Investigators must record in the CRF their opinion concerning the

relationship of the adverse event to study therapy. All measures required for adverse event management must be recorded in the source document and reported according to sponsor instructions.

The sponsor assumes responsibility for appropriate reporting of adverse events to the regulatory authorities. The sponsor will also report to the investigator (and the head of the investigational institute where required) all suspected unexpected serious adverse reactions (SUSARs). The investigator (or sponsor where required) must report SUSARs to the appropriate Independent Ethics Committee/Institutional Review Board (IEC/IRB) that approved the protocol unless otherwise required and documented by the IEC/IRB.

Subjects (or their designees, if appropriate) must be provided with a "wallet (study) card" and instructed to carry this card with them for the duration of the study indicating the following:

- Study number
- Investigator's name and 24-hour contact telephone number
- Local sponsor's name and 24-hour contact telephone number (for medical staff only)
- Statement, in the local language(s), that the subject is participating in a clinical study.
- Site number
- Subject number

12.3.2. Serious Adverse Events

All serious adverse events occurring during the study must be reported to the appropriate sponsor contact person by study-site personnel within 24 hours of their knowledge of the event.

Information regarding serious adverse events will be transmitted to the sponsor using the Serious Adverse Event Form, which must be completed and signed by a physician from the study site, and transmitted to the sponsor within 24 hours. The initial and follow-up reports of a serious adverse event should be made by facsimile (fax).

All serious adverse events that have not resolved upon discontinuation of the subject's participation in the study, must be followed until any of the following occurs:

- The event resolves
- The event stabilizes (as noted in Section 9.5).
- The event returns to baseline, if a baseline value/status is available
- The event can be attributed to agents other than the study drug or to factors unrelated to study conduct
- It becomes unlikely that any additional information can be obtained (subject or health care practitioner refusal to provide additional information, lost to follow-up after demonstration of due diligence with follow-up efforts)

- The study ends (when post-trial access to DOXIL is available for subjects remaining on single-agent DOXIL or until disease progression, whichever occurs first).

Suspected transmission of an infectious agent by a medicinal product will be reported as a serious adverse event. Any event requiring hospitalization (or prolongation of hospitalization) that occurs during the course of a subject's participation in a study must be reported as a serious adverse event, except hospitalizations for the following:

- Hospitalizations not intended to treat an acute illness or adverse event (eg, social reasons such as pending placement in long-term care facility).
- Surgery or procedure planned before entry into the study (must be documented in the CRF). Note: Hospitalizations that were planned before the signing of the ICF, and where the underlying condition for which the hospitalization was planned has not worsened, will not be considered serious adverse events. Any adverse event that results in a prolongation of the originally planned hospitalization is to be reported as a new serious adverse event.
- For reasons described in the protocol, eg, study drug administration, protocol-required testing.
- For the administration of blood or platelet transfusion. Hospitalization or prolonged hospitalization for a complication of such transfusion remains a reportable serious adverse event.
- As part of a procedure for protocol/disease related investigations (eg, surgery, scans, endoscopy, sampling for laboratory tests, bone marrow sampling). Hospitalization or prolonged hospitalization for a complication of such a procedure remains a reportable serious adverse event.

The cause of death of a subject in a study within 30 days of last dose of study drug, whether or not the event is expected or associated with the study drug, is considered a serious adverse event.

Disease progression should not be recorded as an adverse event or serious adverse event term; instead, signs and symptoms of clinical sequelae resulting from disease progression/lack of efficacy will be reported if they fulfill the serious adverse event definition (see Section 12.1.1, Adverse Event Definitions and Classifications).

12.3.3. Pregnancy

All initial reports of pregnancy must be reported to the sponsor by the study-site personnel within 24 hours of their knowledge of the event using the Pregnancy Notification Form. Abnormal pregnancy outcomes (eg, spontaneous abortion, fetal death, stillbirth, congenital anomalies, and ectopic pregnancy) are considered serious adverse events and must be reported using the Serious Adverse Event Form. Any subject who becomes pregnant during the study must be promptly withdrawn from the study. Follow-up information regarding the outcome of the pregnancy and any postnatal sequelae in the infant will be required.

12.4. Contacting Sponsor Regarding Safety

The names (and corresponding telephone numbers) of the individuals who should be contacted regarding safety issues or questions regarding the study are listed on the Contact Information page(s), which will be provided as a separate document.

13. PRODUCT QUALITY COMPLAINT HANDLING

A product quality complaint (PQC) is defined as any suspicion of a product defect related to manufacturing, labeling, or packaging, ie, any dissatisfaction relative to the identity, quality, durability, or reliability of a product, including its labeling or package integrity. PQCs may have an impact on the safety and efficacy of the product. Timely, accurate, and complete reporting and analysis of PQC information from studies are crucial for the protection of subjects, investigators, and the sponsor, and are mandated by regulatory agencies worldwide. The sponsor has established procedures in conformity with regulatory requirements worldwide to ensure appropriate reporting of PQC information; all studies conducted by the sponsor or its affiliates will be conducted in accordance with those procedures.

13.1. Procedures

All initial PQCs must be reported to the sponsor by the study-site staff within 24 hours after being made aware of the event.

If the defect is combined with a serious adverse event, the study-site personnel must report the PQC to the sponsor according to the serious adverse event reporting timelines (refer to Section 12.3.2, Serious Adverse Events). A sample of the suspected product should be maintained for further investigation if requested by the sponsor.

13.2. Contacting Sponsor Regarding Product Quality

The names (and corresponding telephone numbers) of the individuals who should be contacted regarding product quality issues are listed on the Contact Information page(s), which will be provided as a separate document.

14. STUDY DRUG INFORMATION

As of Amendment 6, subjects in treatment Arm A will discontinue treatment with trabectedin. All study subjects (Arm A or Arm B) currently on study treatment who, in the opinion of the investigator, are deriving clinical benefit may continue treatment with single-agent DOXIL as per the local standard of care. Treatment for these subjects may continue as long as the subjects comply with protocol-specified prohibitions and restrictions for treatment (Section 4.3) and as long as they experience clinical benefit in the opinion of the investigator. DOXIL will be provided by JR&D as needed until post-trial access to DOXIL is available or until disease progression, whichever occurs first.

14.1. Physical Description of Study Drug(s)

The trabectedin (YONDELIS® IV formulation) supplied for this study is a white to off-white powder for reconstitution, dilution, and IV infusion. Each vial of trabectedin for injection is a

single-use vial. It will be manufactured and provided under the responsibility of the sponsor. Refer to the Investigator's Brochure for a list of excipients.

DOXIL will be supplied as a sterile, translucent, red liposomal dispersion. Refer to the Investigator's Brochure and package insert for a list of excipients.

14.2. Packaging

The study drug will be packaged in individual kits. Each kit will consist of 1 vial of study drug (trabectedin or DOXIL). Trabectedin is provided by the sponsor as a sterile lyophilized product in vials containing 1.0 mg of trabectedin.

DOXIL is provided by the sponsor in glass, single-use vials. Each vial contains either 20 mg of DOXIL at a concentration of 2 mg/mL or 50 mg of DOXIL at a concentration of 2 mg/mL. See the package insert for more details.

14.3. Labeling

Study drug labels will contain information to meet the applicable regulatory requirements.

14.4. Preparation, Handling, and Storage

Trabectedin

Refer to the Investigator's Brochure of YONDELIS (provided as a separate document) for instructions regarding drug inventory, handling, reconstitution, dilution, storage, accountability, and disposal.

Trabectedin is a cytotoxic anticancer medicinal product, and caution should be exercised during handling.

No incompatibilities have been observed between YONDELIS and Type 1 glass vials, polyvinylchloride, polyethylene, polyethylene and polypropylene mixture bags, polyvinylchloride or polyethylene tubing, or titanium or plastic resin implantable vascular access systems.

Only calibrated infusion pumps with the above product contact surfaces should be used to deliver the drug.

All trabectedin study drug must be stored at controlled temperatures ranging from 2ºC to 8ºC (36ºF to 46ºF) in a refrigerator.

DOXIL

The vials contain either 20 mg of doxorubicin HCl in 10 mL or 50 mg of doxorubicin HCl in 25 mL (both at a concentration of 2 mg doxorubicin HCl/mL), unless specified otherwise on the label. Determine the volume of DOXIL to be administered (based on the dose specified in the protocol, see Section 6, Dosage and Administration).

Take the appropriate volume of DOXIL up into a sterile syringe. Aseptic technique must be strictly observed since no preservative or bacteriostatic agent is present in DOXIL. Each vial is for single use only.

Dilute the DOXIL in D5W. For doses ≥12 mg and <90 mg, dilute in 250 mL of D5W. For doses ≥90 mg, dilute in 500 mL of D5W. Once the DOXIL has been diluted, it must be kept refrigerated at 2°C to 8°C and administered within 24 hours of mixing. See Section 6, Dosage and Administration, for detailed instructions on administering DOXIL in this study. Infusion may be given through a peripheral vein or a central line.

DOXIL should not be mixed with other drugs until specific compatibility data are available.

The presence of any bacteriostatic agent, such as benzyl alcohol, may cause precipitation of DOXIL.

Caution should be exercised in handling the DOXIL solution. The use of gloves is recommended. If DOXIL comes into contact with skin or mucosa, immediately wash thoroughly with soap and water.

DOXIL should be handled and disposed of in a manner consistent with other anticancer drugs.

All DOXIL study drug must be stored at controlled temperatures ranging 2°C to 8°C (36°F to 46°F) in a refrigerator. Do not freeze.

14.5. Drug Accountability

The investigator is responsible for ensuring that all study drug received at the site is inventoried and accounted for throughout the study. The study drug administered to the subject must be documented on the drug accountability form. All study drug will be stored and disposed of according to the sponsor's instructions. Study-site personnel must not combine contents of the study drug containers.

Study drug must be handled in strict accordance with the protocol and the container label, and must be stored at the study site in a limited-access area or in a locked cabinet under appropriate environmental conditions. Unused study drug must be available for verification by the sponsor's site monitor during on-site monitoring visits. The return to the sponsor of unused study drug, or used returned study drug for destruction, will be documented on the Drug Return Form. When the study site is an authorized destruction unit and study drug supplies are destroyed on site, this must also be documented on the Drug Return Form.

Potentially hazardous materials such as used ampules, needles, syringes and vials containing hazardous liquids, should be disposed of immediately in a safe manner and therefore will not be retained for drug accountability purposes.

Study drug should be dispensed under the supervision of the investigator or a qualified member of the study-site personnel, or by a hospital/clinic pharmacist. Study drug will be supplied only

to subjects participating in the study. Returned study drug must not be dispensed again, even to the same subject. Study drug may not be relabeled or reassigned for use by other subjects. The investigator agrees neither to dispense the study drug from, nor store it at, any site other than the study sites agreed upon with the sponsor.

15. STUDY-SPECIFIC MATERIALS

The investigator will be provided with the following supplies:

- Investigator's Brochure (trabectedin and DOXIL)
- Case report forms
- Laboratory supplies to process samples that will be sent to the central laboratory, including study manuals
- Package insert for DOXIL
- NCI-CTCAE, Version 4.0
- QLQ-OV28 and Euro EQ-5D-5L questionnaires
- Investigator file binder to keep all documents concerning the trial.
- RECIST guidelines Version 1.1
- Patient Qualification Form
- IVRS/IWRS Manual and codes. These are part of the Investigator file but are supplied in a separate binder.
- An IDMC has been commissioned for this study. Details regarding objectives and procedures for this committee are contained in a separate Charter that will accompany the protocol in regions where this is required.
- Electronic data capture (eDC) Manual

16. ETHICAL ASPECTS

16.1. Study-Specific Design Considerations

The current trial is a randomized, open-label study. As the experimental agent trabectedin is administered via a central catheter, for ethical reasons the comparator control is generally administered via a peripheral vein. The study is open label because blinding of the treatment arms is not possible due to the mode of administration and administration schedules of the study drugs. The single-agent arm (DOXIL) has a slightly higher dose of DOXIL (dose as per the product package insert) while the combination (trabectedin+DOXIL) combines the 2 agents at lower doses. Criteria for further dose adjustments are outlined for each study arm. The study population is well defined (subjects with platinum-sensitive advanced-relapsed epithelial ovarian, primary peritoneal, or fallopian tube cancer who have received 2 previous lines of platinum-based chemotherapy) and will receive the standard of care therapy in both arms of the trial. Subjects will be fully informed of the potential risks of treatment with the study drugs, and can withdraw consent at any time.

The trabectedin+DOXIL combination therapy has shown better efficacy (prolonged PFS) than DOXIL monotherapy in the second-line treatment of refractory ovarian cancer in Study ET743-OVA-301. Based on a retrospective analysis of OS in Study ET743-OVA-301, subjects with platinum-sensitive disease who receive the trabectedin+DOXIL combination therapy may also experience prolonged survival. Because trabectedin is added to DOXIL, the combination therapy is expected to result in a higher incidence of some adverse events when compared to the DOXIL monotherapy comparator arm.

The retrospective analysis of OS data in Study ET743-OVA-301 indicates that the lack of statistical significance for OS resulted from an imbalance in prognostic factors (PFI) at randomization. BRCA 1/2 mutation status is a key prognostic factor in ovarian cancer[15,16]. Therefore, subjects in this study will be stratified by BRCA 1/2 mutation status (mutation vs no mutation). The mutation status will only be used to stratify the subjects to treatment (trabectedin+DOXIL or DOXIL). Depending upon the region, it is expected that as many as 50% of potential subjects will have a known BRCA 1/2 status, as this testing is routinely used in standard medical practice to assess genetic risk to specific cancers[15,16]. For those subjects without a known BRCA 1/2 status, genetic analysis of BRCA 1/2 will be required and, therefore, performed as a study-related procedure. The results of this analysis will be reported to the investigator.

All participating subjects will receive full supportive care and will be followed closely for safety and efficacy throughout the trial. Efficacy assessments will occur according to the internationally accepted response criteria. Safety assessments will occur through regular clinic visits including laboratory analyses. It is anticipated that the volumes of blood drawn for each subject for routine laboratory evaluations throughout the study will be approximately 15 mL for screening serum chemistry and hematology tests; 10 mL for BRCA 1/2 testing at screening (for subjects who do not have a known BRCA 1/2 status); 4 mL for subjects consenting to the optional pharmacogenomics component of the study (where local regulations permit); 45 mL and 60 mL for treatment Arms A and B, respectively, per cycle for serum biochemistry and hematology tests; 4 mL per scheduled CA-125 sample; and 24 mL from those subjects who provide PK samples. Overall, the volume of blood to be drawn is deemed reasonable over the time frame of the study. An IDMC will follow safety and efficacy during the study at regular intervals (approximately every 6 months) and in the prespecified interim analysis, respectively.

Note: Following the review of the study data by the IDMC on 15 December 2017, the HR for OS was 0.962, crossing the previously agreed upon threshold for futility of 0.93. In view of this result and the observed and expected higher toxicity in Arm A as compared with Arm B, the IDMC recommended discontinuing the study. Therefore, as of Amendment 6, no new subjects will be randomized to study treatment, and treatment with trabectedin should be immediately discontinued for subjects assigned to Arm A (trabectedin+DOXIL). All study subjects (Arm A or Arm B) currently on study treatment who, in the opinion of the investigator, are deriving clinical benefit may continue treatment with single-agent DOXIL as per the local standard of care. Treatment for these subjects may continue as long as the subjects comply with protocol-specified prohibitions and restrictions for treatment (Section 4.3) and as long as they experience clinical

benefit in the opinion of the investigator. DOXIL will be provided by JR&D as needed until post-trial access to DOXIL is available or until disease progression, whichever occurs first.

All subjects still on study treatment should be informed of the reasons for the discontinuation of the study, and the potential risks and benefits of continued treatment with single-agent DOXIL should be discussed.

Potential subjects will be fully informed of the risks and requirements of the study and, during the study, subjects will be given any new information that may affect their decision to continue participation. They will be told that their consent to participate in the study is voluntary and may be withdrawn at any time with no reason given and without penalty or loss of benefits to which they would otherwise be entitled. Only subjects who are fully able to understand the risks, benefits, and potential adverse events of the study, and provide their consent voluntarily will be enrolled.

16.2. Regulatory Ethics Compliance

16.2.1. Investigator Responsibilities

The investigator is responsible for ensuring that the clinical study is performed in accordance with the protocol, current ICH guidelines on Good Clinical Practice (GCP), and applicable regulatory and country-specific requirements.

Good Clinical Practice is an international ethical and scientific quality standard for designing, conducting, recording, and reporting studies that involve the participation of human subjects. Compliance with this standard provides public assurance that the rights, safety, and well-being of study subjects are protected, consistent with the principles that originated in the Declaration of Helsinki, and that the clinical study data are credible.

16.2.2. Independent Ethics Committee or Institutional Review Board

Before the start of the study, the investigator (or sponsor where required) will provide the IEC/IRB with current and complete copies of the following documents (as required by local regulations):

- Final protocol and, if applicable, amendments
- Sponsor-approved ICF (and any other written materials to be provided to the subjects)
- Investigator's Brochure (or equivalent information) and amendments/addenda
- Sponsor-approved subject recruiting materials
- Information on compensation for study-related injuries or payment to subjects for participation in the study, if applicable
- Investigator's curriculum vitae or equivalent information (unless not required, as documented by the IEC/IRB)
- Information regarding funding, name of the sponsor, institutional affiliations, other potential conflicts of interest, and incentives for subjects

- Any other documents that the IEC/IRB requests to fulfill its obligation

This study will be undertaken only after the IEC/IRB has given full approval of the final protocol, amendments (if any, excluding the ones that are purely administrative, with no consequences for subjects, data, or study conduct, unless required locally), the ICF, applicable recruiting materials, and subject compensation programs, and the sponsor has received a copy of this approval. This approval letter must be dated and must clearly identify the IEC/IRB and the documents being approved.

Approval for the collection of optional samples for research and for the corresponding ICF must be obtained from the IEC/IRB. Approval for the protocol can be obtained independent of this optional research component.

During the study, the investigator (or sponsor where required) will send the following documents and updates to the IEC/IRB for their review and approval, where appropriate:

- Protocol amendments (excluding the ones that are purely administrative, with no consequences for subjects, data, or study conduct)
- Revision(s) to ICF and any other written materials to be provided to subjects
- If applicable, new or revised subject recruiting materials approved by the sponsor
- Revisions to compensation for study-related injuries or payment to subjects for participation in the study, if applicable
- New edition(s) of the Investigator's Brochure and amendments/addenda
- Summaries of the status of the study at intervals stipulated in guidelines of the IEC/IRB (at least annually)
- Reports of adverse events that are serious, unlisted/unexpected, and associated with the study drug
- New information that may adversely affect the safety of the subjects or the conduct of the study
- Deviations from or changes to the protocol to eliminate immediate hazards to the subjects
- Report of deaths of subjects under the investigator's care
- Notification if a new investigator is responsible for the study at the site
- Development Safety Update Report and Line Listings, where applicable
- Any other requirements of the IEC/IRB

For all protocol amendments (excluding the ones that are purely administrative, with no consequences for subjects, data, or trial conduct), the amendment and applicable ICF revisions must be submitted promptly to the IEC/IRB for review and approval before implementation of the change(s).

At least once a year, the IEC/IRB will be asked to review and reapprove this study. The reapproval should be documented in writing (excluding the ones that are purely administrative, with no consequences for subjects, data, or study conduct).

At the end of the study, the investigator (or sponsor where required) will notify the IEC/IRB about the study completion.

16.2.3. Informed Consent

Each subject (or a legally acceptable representative) must give written consent according to local requirements after the nature of the study has been fully explained. The ICF(s) must be signed before performance of any study-related activity. The ICF(s) that is/are used must be approved by both the sponsor and by the reviewing IEC/IRB and be in a language that the subject can read and understand. The informed consent should be in accordance with principles that originated in the Declaration of Helsinki, current ICH and GCP guidelines, applicable regulatory requirements, and sponsor policy.

Before enrollment in the study, the investigator or an authorized member of the study-site personnel must explain to potential subjects or their legally acceptable representatives the aims, methods, reasonably anticipated benefits, and potential hazards of the study, and any discomfort participation in the study may entail. Subjects will be informed that their participation is voluntary and that they may withdraw consent to participate at any time. They will be informed that choosing not to participate will not affect the care the subject will receive for the treatment of her disease. Subjects will be told that alternative treatments are available if they refuse to take part and that such refusal will not prejudice future treatment. Finally, they will be told that the investigator will maintain a subject identification register for the purposes of long-term follow up if needed and that their records may be accessed by health authorities and authorized sponsor personnel without violating the confidentiality of the subject, to the extent permitted by the applicable law(s) or regulations. By signing the ICF the subject or legally acceptable representative is authorizing such access, including permission to obtain information about her survival status, and agrees to allow her study physician to recontact the subject for the purpose of obtaining consent for additional safety evaluations, if needed, and subsequent disease-related treatments, or to obtain information about her survival status.

The subject or legally acceptable representative will be given sufficient time to read the ICF and the opportunity to ask questions. After this explanation and before entry into the study, consent should be appropriately recorded by means of either the subject's or her legally acceptable representative's personally dated signature. After having obtained the consent, a copy of the ICF must be given to the subject.

Subjects will be asked to consent to provide optional samples for research (where local regulations permit). After informed consent for the clinical study is appropriately obtained, the subject or her legally acceptable representative will be asked to sign and personally date a separate ICF indicating agreement to participate in optional research component. Refusal to

participate in the optional research will not result in ineligibility for the study. A copy of this signed ICF will be given to the subject.

Where local regulations require, a separate ICF may be used for the required DNA component of the study.

If the subject or legally acceptable representative is unable to read or write, an impartial witness should be present for the entire informed consent process (which includes reading and explaining all written information) and should personally date and sign the ICF after the oral consent of the subject or legally acceptable representative is obtained.

16.2.4. Privacy of Personal Data

The collection and processing of personal data from subjects enrolled in this study will be limited to those data that are necessary to fulfill the objectives of the study.

These data must be collected and processed with adequate precautions to ensure confidentiality and compliance with applicable data privacy protection laws and regulations. Appropriate technical and organizational measures to protect the personal data against unauthorized disclosures or access, accidental or unlawful destruction, or accidental loss or alteration must be put in place. Sponsor personnel whose responsibilities require access to personal data agree to keep the identity of study subjects confidential.

The informed consent obtained from the subject (or her legally acceptable representative) includes explicit consent for the processing of personal data and for the investigator/institution to allow direct access to his or her original medical records (source data/documents) for study-related monitoring, audit, IEC/IRB review, and regulatory inspection. This consent also addresses the transfer of the data to other entities and to other countries.

The subject has the right to request through the investigator access to her personal data and the right to request rectification of any data that are not correct or complete. Reasonable steps will be taken to respond to such a request, taking into consideration the nature of the request, the conditions of the study, and the applicable laws and regulations.

Exploratory DNA research is not conducted under standards appropriate for the return of data to subjects. In addition, the sponsor cannot make decisions as to the significance of any findings resulting from exploratory research. Therefore, exploratory research data will not be returned to subjects or investigators, unless required by law or local regulations. Privacy and confidentiality of data generated in the future on stored samples will be protected by the same standards applicable to all other clinical data.

16.2.5. Long-Term Storage of Samples for Future Research

Samples collected in this study may be stored for up to 15 years (or according to local regulations) for additional research. Samples will only be used to understand trabectedin/DOXIL or advanced-relapsed epithelial ovarian, primary peritoneal, or fallopian tube cancer, to understand differential drug responders, and to develop tests/assays related to

trabectedin/DOXIL or advanced-relapsed epithelial ovarian, primary peritoneal, or fallopian tube cancer. The research may begin at any time during the study or the post-study storage period.

Stored samples will be coded throughout the sample storage and analysis process and will not be labeled with personal identifiers. Subjects may withdraw their consent for their samples to be stored for research (See Section 10.3, Withdrawal From the Study).

16.2.6. Country Selection

This study will only be conducted in those countries where the intent is to launch or otherwise help ensure access to the developed product, unless explicitly addressed as a specific ethical consideration in Section 16.1, Study-Specific Design Considerations.

17. ADMINISTRATIVE REQUIREMENTS

17.1. Protocol Amendments

Neither the investigator nor the sponsor will modify this protocol without a formal amendment by the sponsor. All protocol amendments must be issued by the sponsor, and signed and dated by the investigator. Protocol amendments must not be implemented without prior IEC/IRB approval, or when the relevant competent authority has raised any grounds for non-acceptance, except when necessary to eliminate immediate hazards to the subjects, in which case the amendment must be promptly submitted to the IEC/IRB and relevant competent authority. Documentation of amendment approval by the investigator and IEC/IRB must be provided to the sponsor. When the change(s) involves only logistic or administrative aspects of the study, the IRB (and IEC where required) only needs to be notified.

During the course of the study, in situations where a departure from the protocol is unavoidable, the investigator or other physician in attendance will contact the appropriate sponsor representative listed in the Contact Information page(s), which will be provided as a separate document. Except in emergency situations, this contact should be made before implementing any departure from the protocol. In all cases, contact with the sponsor must be made as soon as possible to discuss the situation and agree on an appropriate course of action. The data recorded in the CRF and source documents will reflect any departure from the protocol, and the source documents will describe this departure and the circumstances requiring it.

17.2. Regulatory Documentation

17.2.1. Regulatory Approval/Notification

This protocol and any amendment(s) must be submitted to the appropriate regulatory authorities in each respective country, if applicable. A study may not be initiated until all local regulatory requirements are met.

17.2.2. Required Prestudy Documentation

The following documents must be provided to the sponsor before shipment of study drug to the study site:

- Protocol and amendment(s), if any, signed and dated by the principal investigator
- A copy of the dated and signed (or sealed, where appropriate per local regulations), written IEC/IRB approval of the protocol, amendments, ICF, any recruiting materials, and if applicable, subject compensation programs. This approval must clearly identify the specific protocol by title and number and must be signed (or sealed, where appropriate per local regulations) by the chairman or authorized designee.
- Name and address of the IEC/IRB, including a current list of the IEC/IRB members and their function, with a statement that it is organized and operates according to GCP and the applicable laws and regulations. If accompanied by a letter of explanation, or equivalent, from the IEC/IRB, a general statement may be substituted for this list. If an investigator or a member of the study-site personnel is a member of the IEC/IRB, documentation must be obtained to state that this person did not participate in the deliberations or in the vote/opinion of the study.
- Regulatory authority approval or notification, if applicable
- Signed and dated statement of investigator (eg, Form FDA 1572), if applicable
- Documentation of investigator qualifications (eg, curriculum vitae)
- Completed investigator financial disclosure form from the principal investigator, where required
- Signed and dated clinical trial agreement, which includes the financial agreement
- Any other documentation required by local regulations

The following documents must be provided to the sponsor before enrollment of the first subject:

- Completed investigator financial disclosure forms from all clinical sub-investigators
- Documentation of sub-investigator qualifications (eg, curriculum vitae)
- Name and address of any local laboratory conducting tests for the study, and a dated copy of current laboratory normal ranges for these tests, if applicable
- Local laboratory documentation demonstrating competence and test reliability (eg, accreditation/license), if applicable.

17.3. Subject Identification, Enrollment, and Screening Logs

The investigator agrees to complete a subject identification and enrollment log to permit easy identification of each subject during and after the study. This document will be reviewed by the sponsor study site contact for completeness.

The subject identification and enrollment log will be treated as confidential and will be filed by the investigator in the study file. To ensure subject confidentiality, no copy will be made. All

reports and communications relating to the study will identify subjects by subject identification and date of birth. In cases where the subject is not randomized into the study, the date seen and date of birth will be used.

The investigator must also complete a subject screening log, which reports on all subjects who were seen to determine eligibility for inclusion in the study.

17.4. Source Documentation

At a minimum, source documentation must be available for the following to confirm data collected in the CRF: subject identification, eligibility, and study identification; study discussion and date of informed consent; dates of visits; results of safety and efficacy parameters as required by the protocol; record of all adverse events and follow-up of adverse events; concomitant medication; drug receipt/dispensing/return records; study drug administration information; and date of study completion and reason for early discontinuation of study drug or withdrawal from the study, if applicable.

The author of an entry in the source documents should be identifiable.

Specific details required as source data for the study and source data collection methods will be reviewed with the investigator before the study and will be described in the monitoring guidelines (or other equivalent document).

Inclusion and exclusion criteria not requiring documented medical history must be verified at a minimum by subject interview or other protocol required assessment (eg, physical examination, laboratory assessment) and documented in the source documents.

17.5. Case Report Form Completion

Case report forms are provided for each subject in printed or electronic format. All CRF entries, corrections, and alterations must be made by the investigator or authorized study-site personnel. The investigator must verify that all data entries in the CRF are accurate and correct.

Electronic Data Capture (eDC) will be used for this study. The study data will be transcribed by study-site personnel from the source documents onto an electronic CRF, and transmitted in a secure manner to the sponsor within the timeframe agreed upon between the sponsor and the study site. The electronic file will be considered to be the CRF.

Worksheets may be used for the capture of some data to facilitate completion of the CRF. Any such worksheets will become part of the subject's source documentation. All data relating to the study must be recorded in CRFs prepared by the sponsor. Data must be entered into CRFs in English. Study-site personnel must complete the CRF as soon as possible after a subject visit, and the forms should be available for review at the next scheduled monitoring visit.

The investigator must verify that all data entries in the CRFs are accurate and correct. All CRF entries, corrections, and alterations must be made by the investigator or other authorized study-site personnel. If necessary, queries will be generated in the eDC tool. If corrections to a

CRF are needed after the initial entry into the CRF, this can be done in either of the following ways:

- Investigator and study-site personnel can make corrections in the eDC tool at their own initiative or as a response to an auto query (generated by the eDC tool).
- Sponsor or sponsor delegate can generate a query for resolution by the investigator and study-site personnel.

17.6. Data Quality Assurance/Quality Control

Steps to be taken to ensure the accuracy and reliability of data include the selection of qualified investigators and appropriate study sites, review of protocol procedures with the investigator and study-site personnel before the study, and periodic monitoring visits by the sponsor. Written instructions will be provided for collection, preparation, and shipment of samples.

Guidelines for CRF completion will be provided and reviewed with study-site personnel before the start of the study. The sponsor will review CRFs for accuracy and completeness during on-site monitoring visits and after transmission to the sponsor; any discrepancies will be resolved with the investigator or designee, as appropriate. After the upload of the data into the study database, they will be verified for accuracy and consistency with the data sources.

17.7. Record Retention

In compliance with the ICH/GCP guidelines, the investigator/institution will maintain all CRFs and all source documents that support the data collected from each subject, as well as all study documents as specified in ICH/GCP Section 8, Essential Documents for the Conduct of a Clinical Trial, and all study documents as specified by the applicable regulatory requirement(s). The investigator/institution will take measures to prevent accidental or premature destruction of these documents.

Essential documents must be retained until at least 2 years after the last approval of a marketing application in an ICH region and until there are no pending or contemplated marketing applications in an ICH region or until at least 2 years have elapsed since the formal discontinuation of clinical development of the investigational product. These documents will be retained for a longer period if required by the applicable regulatory requirements or by an agreement with the sponsor. It is the responsibility of the sponsor to inform the investigator/institution as to when these documents no longer need to be retained.

If the responsible investigator retires, relocates, or for other reasons withdraws from the responsibility of keeping the study records, custody must be transferred to a person who will accept the responsibility. The sponsor must be notified in writing of the name and address of the new custodian. Under no circumstance shall the investigator relocate or dispose of any study documents before having obtained written approval from the sponsor.

If it becomes necessary for the sponsor or the appropriate regulatory authority to review any documentation relating to this study, the investigator/institution must permit access to such reports.

17.8. Monitoring

The sponsor will use a combination of monitoring techniques (central, remote, or on-site monitoring) to monitor the study.

The sponsor will perform on-site monitoring visits as frequently as necessary. The monitor will record dates of the visits in a study site visit log that will be kept at the study site. The first post-initiation visit will be made as soon as possible after enrollment has begun. At these visits, the monitor will compare the data entered into the CRFs with the source documents (eg, hospital/clinic/physician's office medical records). The nature and location of all source documents will be identified to ensure that all sources of original data required to complete the CRF are known to the sponsor and study-site personnel and are accessible for verification by the sponsor study-site contact. If electronic records are maintained at the study site, the method of verification must be discussed with the study-site personnel.

Direct access to source documentation (medical records) must be allowed for the purpose of verifying that the data recorded in the CRF are consistent with the original source data. Findings from this review of CRFs and source documents will be discussed with the study-site personnel. The sponsor expects that, during monitoring visits, the relevant study-site personnel will be available, the source documentation will be accessible, and a suitable environment will be provided for review of study-related documents. The monitor will meet with the investigator on a regular basis during the study to provide feedback on the study conduct.

In addition, to on-site monitoring visits, remote contact can occur. It is expected that during these remote contacts, study-site personnel will be available to provide an update on the progress of the study at the site.

Central monitoring will take place for data identified by the sponsor as requiring central review.

17.9. Study Completion/Termination

17.9.1. Study Completion

The study is considered completed with the last visit for the last subject participating in the study. The final data from the study site will be sent to the sponsor (or designee) after completion of the final subject visit at that study site, in the time frame specified in the Clinical Trial Agreement.

17.9.2. Study Termination

The sponsor reserves the right to close the study site or terminate the study at any time for any reason at the sole discretion of the sponsor. Study sites will be closed upon study completion. A

study site is considered closed when all required documents and study supplies have been collected and a study-site closure visit has been performed.

The investigator may initiate study-site closure at any time, provided there is reasonable cause and sufficient notice is given in advance of the intended termination.

Reasons for the early closure of a study site by the sponsor or investigator may include but are not limited to:

- Failure of the investigator to comply with the protocol, the requirements of the IEC/IRB or local health authorities, the sponsor's procedures, or GCP guidelines
- Inadequate recruitment of subjects by the investigator
- Discontinuation of further drug development

17.10. On-Site Audits

Representatives of the sponsor's clinical quality assurance department may visit the study site at any time during or after completion of the study to conduct an audit of the study in compliance with regulatory guidelines and company policy. These audits will require access to all study records, including source documents, for inspection and comparison with the CRFs. Subject privacy must, however, be respected. The investigator and study-site personnel are responsible for being present and available for consultation during routinely scheduled study-site audit visits conducted by the sponsor or its designees.

Similar auditing procedures may also be conducted by agents of any regulatory body, either as part of a national GCP compliance program or to review the results of this study in support of a regulatory submission. The investigator should immediately notify the sponsor if he or she has been contacted by a regulatory agency concerning an upcoming inspection.

17.11. Use of Information and Publication

All information, including but not limited to information regarding trabectedin and DOXIL or the sponsor's operations (eg, patent application, formulas, manufacturing processes, basic scientific data, prior clinical data, formulation information) supplied by the sponsor to the investigator and not previously published, and any data, including pharmacogenomic research data, generated as a result of this study, are considered confidential and remain the sole property of the sponsor. The investigator agrees to maintain this information in confidence and use this information only to accomplish this study, and will not use it for other purposes without the sponsor's prior written consent.

The investigator understands that the information developed in the study will be used by the sponsor in connection with the continued development of trabectedin and DOXIL, and thus may be disclosed as required to other clinical investigators or regulatory agencies. To permit the information derived from the clinical studies to be used, the investigator is obligated to provide the sponsor with all data obtained in the study.

The results of the study will be reported in a Clinical Study Report generated by the sponsor and will contain CRF data from all study sites that participated in the study. Recruitment performance or specific expertise related to the nature and the key assessment parameters of the study will be used to determine a coordinating investigator. Results of pharmacogenomics analyses performed after the Clinical Study Report has been issued will be reported in a separate report and will not require a revision of the Clinical Study Report. Study subject identifiers will not be used in publication of results. Any work created in connection with performance of the study and contained in the data that can benefit from copyright protection (except any publication by the investigator as provided for below) shall be the property of the sponsor as author and owner of copyright in such work.

Consistent with Good Publication Practices and International Committee of Medical Journal Editors guidelines, the sponsor shall have the right to publish such primary (multicenter) data and information without approval from the investigator. Subject to the publication terms under the Clinical Trial Agreement, the investigator has the right to publish study site-specific data after the primary data are published. If an investigator wishes to publish information from the study, a copy of the manuscript must be provided to the sponsor for review at least 60 days before submission for publication or presentation. Expedited reviews will be arranged for abstracts, poster presentations, or other materials. If requested by the sponsor in writing, the investigator will withhold such publication for up to an additional 60 days to allow for filing of a patent application. In the event that issues arise regarding scientific integrity or regulatory compliance, the sponsor will review these issues with the investigator. For multicenter study designs and substudy approaches, secondary results generally should not be published before the primary endpoints of a study have been published. Similarly, investigators will recognize the integrity of a multicenter study by not submitting for publication data derived from the individual study site until the combined results from the completed study have been submitted for publication, within 12 months of the availability of the final data (tables, listings, graphs), or the sponsor confirms there will be no multicenter study publication. Authorship of publications resulting from this study will be based on the guidelines on authorship, such as those described in the Uniform Requirements for Manuscripts Submitted to Biomedical Journals, which state that the named authors must have made a significant contribution to the design of the study or analysis and interpretation of the data, provided critical review of the paper, and given final approval of the final version.

Registration of Clinical Studies and Disclosure of Results

The sponsor will register and/or disclose the existence of and the results of clinical studies as required by law.

REFERENCES

1. Allavena P (2005), Signorelli M, Chieppa M, et al. Anti-inflammatory properties of the novel antitumor agent yondelis (trabectedin): inhibition of macrophage differentiation and cytokine production. Cancer Res 65:2964-71.

2. ASCO Guidelines (1999a). Recommendations for the use of antiemetics: evidence-based clinical practice guidelines. Health services research committee of the American Society of Clinical Oncology. J Clin Oncol; 17(9): 2971.

3. ASCO Guidelines (1999b) Use of hematopoietic colony-stimulating factors: comparison of the 1994 and 1997 American Society of Clinical Oncology surveys regarding ASCO clinical practice guidelines. Health Services Research Committee of the American Society of Clinical Oncology. J Clin Oncol 1999 17:3676-3681.

4. Clinical Study Report ET743-OVA-301 (2011). An open-label, multicenter, randomized, phase 3 study comparing the combination of YONDELIS® with DOXIL®/CAELYX® or DOXIL/CAELYX alone in subjects with advanced relapsed ovarian cancer. Document IDs No.: EDMS-PSDB-39155220:3.0 (30 September 2009), EDMS-ERI-10558930:1.0 (30 June 2010), EDMS-ERI-21283092:2.0 (28 April 2011) Janssen Research & Development, LLC.

5. D'Incalci M (2010) and Galmarini CM. A review of trabectedin (ET-743): a unique mechanism of action. Mol Cancer Ther. 9(8):2157-2163.

6. Erba E (2001), Bergamaschi D, Bassano L, et al. Ecteinascidin-743 (ET-743), a natural marine compound, with a unique mechanism of action. Eur J Cancer;37:97-105.

7. Germano G (2010), Frapolli R, Simone M, et al. Antitumor and anti-inflammatory effects of trabectedin on human myxoid liposarcoma cells. Cancer Res 70:2235–44.

8. Gordon AN (2001), Fleagle JT, Guthrie D, et al. Recurrent epithelial ovarian carcinoma: A randomized phase III study of pegylated liposomal doxorubicin versus topotecan. J Clin Oncol; 19(14): 3312-3322.

9. Gordon AN (2004), Tonda M, Sun S, et al. Long-term survival advantage for women treated with pegylated liposomal doxorubicin compared with topotecan in a phase 3 randomized study of recurrent and refractory epithelial ovarian cancer. Gyn Onc, 95:1-8.

10. Krasner CN (2007), Mc Meekin DS, Chan S, et al. Phase II study of trabectedin single agent in patients with recurrent ovarian cancer previously treated with platinum-based regimens. British Journal of Cancer; 97:1618-1624.

11. McMeekin S (2007), Del Campo JM, Colombo N, et al. Trabectedin in relapsed ovarian cancer (ROC): a pooled analysis of three phase II studies. J Clin Oncol, ASCO Annual Meeting Proceedings, Part 1 25:5579.

12. Minuzzo M (2000), Marchini S, Broggini M, et al. Interference of transcriptional activation by the antineoplastic drug ecteinascidin-743. Proc Natl Acad Sci USA; 97:6780-6784.

13. Monk BJ (2010), Herzog TJ, Kaye SB, et al. Trabectedin plus pegylated doxorubicin in recurrent ovarian cancer. J Clin Oncol; 28:3107-3114.

14. Monk BJ (2012), Herzog TJ, Kaye SB, et al. Trabectedin plus pegylated liposomal doxorubicin (PLS) versus PLD in recurrent ovarian cancer: Overall survival analysis. Eur J Cancer; 48:2361-2368.

15. NCCN Guidelines (2012). Genetic/Familial High-Risk Assessment: Breast and Ovarian Versioun1.2012. Accessed on 13 November 2012. http://www.nccn.org/professionals/physician_gls/PDF/genetics_screening.pdf

16. NCI Fact Sheet (2012). National Cancer Institute Fact Sheet, BRCA1 and BRCA2 Cancer Risk and Genetic Testing. Accessed on 13 November 2012. t http://www.cancer.gov/cancertopics/factsheet/Risk/BRCA

17. Sessa C (2005), De Braud F, Perotti A, et al. Trabectedin for women with ovarian carcinoma after treatment with platinum and taxanes fails. J. Clin Oncol; 23:1867-1874.

18. Takebayashi Y (2001), Pourquier P, Zimonjic DB, et al. Antiproliferative activity of ecteinascidin 743 is dependent upon transcription-coupled nucleotide-excision repair. Nat Med;7:961-966.

19. Von Mehren M (2008), Schilder RJ, Cheng JD, et al. A phase I study of the safety and pharmacokinetics of trabectedin in combination with pegylated liposomal doxorubicin in patients with advanced malignancies. Ann Oncol; 19:1802-1809.

20. [redacted] et al. Phase 2 study of the efficacy and safety of 2 dosing regimens of single agent trabectedin for third line therapy in subjects with advanced breast cancer. Document No.: EDMS-PSDB-3636482, Clinical Study Report (ET743-INT-3)

21. YONDELIS (trabectedin) Investigator's Brochure. Document ID No.: EDMS-ERI-5957767, Janssen Research & Development, LLC.

Attachment 1: Evaluation of Response According to CA-125 (GCIG Criteria)

Definition of response. A response according to CA 125 has occurred if there is at least a 50% reduction in CA 125 levels from a pretreatment sample. The response must be confirmed and maintained for at least 28 days. Patients can be evaluated according to CA 125 only if they have a pretreatment sample that is at least twice the upper limit of normal and within 2 weeks prior to starting treatment.

To calculate CA 125 responses accurately, the following rules apply:

- intervening samples and the 28-day confirmatory sample must be less than or equal to (within an assay variability of 10%) the previous sample.
- variations within the normal range of CA 125 levels will not interfere with the response definition.
- For each patient, the same assay method must be used and the assay must be tested in a quality-control scheme.
- Patients are not evaluable by CA 125 if they have received mouse antibodies (unless the assay used has been shown not to be influenced by HAMA) or if there has been medical and/or surgical interference with their peritoneum or pleura during the previous 28 days. If assessing therapy that includes two treatment modalities for relapse (e.g., surgery and chemotherapy), any CA 125 response results from both treatments modalities. CA 125 cannot distinguish between the effects of the two treatments.

The date when the CA 125 level is first reduced by 50% is the date of the CA 125 response. To calculate response rates, an intent-to-treat analysis should be used that includes all patients with an initial CA 125 level of at least twice the upper limit of normal as eligible and evaluable. In addition, as a separate analysis, those patients who have both a CA 125 response and whose CA 125 level falls to within the normal range, can be classified as CA 125 complete responders. Patients who have a fall of CA 125 to within the normal range but whose initial CA 125 was less than twice the upper limit of normal, have not had a CA 125 response and cannot therefore be classified as a CA 125 complete responder.

Evaluation of response according to CA 125 in patients receiving maintenance or consolidation therapy. Patients whose CA 125 is greater than twice the upper limit of normal when they start maintenance or consolidation therapy can be evaluated according to the GCIG CA 125 response definition. It should be noted that there is no data to validate response evaluation in this situation. To prevent the prior therapy interfering with the response assessment the following requirement is recommended. Two pre-treatment samples no more than 8 weeks apart are required if test treatment is given as part of maintenance or consolidation therapy. For the test treatment to be evaluable according to CA 125 there must be no more than a 10% fall in CA125 between the two pretreatment samples. The sample closest in time to the test therapy should be considered the pre-treatment sample

Attachment 2: RECIST Guidelines

Response Evaluation Criteria in Solid Tumors (RECIST) Version 1.1

The following information was extracted from Section 3, Section 4, and Appendix I of the New response evaluation criteria in solid tumours: revised RECIST guideline (version 1.1) authored by Eisenhauer et al (2009). Refer to the European Journal of Cancer article (2009;45(2):228-247) for the complete publication.

Measurability of tumor at baseline

Definitions

At baseline, tumor lesions/lymph nodes will be categorized measurable or non-measurable as follows:

Measurable:

Tumor lesions: Must be accurately measured in at least one dimension (longest diameter in the plane of measurement is to be recorded) with a *minimum* size of:

- 10 mm by CT scan (CT scan slice thickness no greater than 5 mm)
- 10 mm caliper measurement by clinical exam (lesions which cannot be accurately measured with calipers should be recorded as non-measurable)
- 20 mm by chest X-ray

Non-measurable:

All other lesions, including small lesions (longest diameter <10 mm or pathological lymph nodes with ≥10 to <15 mm short axis) as well as truly non-measurable lesions. Lesions considered truly non-measurable include: leptomeningeal disease, ascites, pleural or pericardial effusion, inflammatory breast disease, lymphangitic involvement of skin or lung, abdominal masses/abdominal organomegaly identified by physical exam that is not measurable by reproducible imaging techniques.

Specifications by methods of measurements

The same method of assessment and the same technique should be used to characterize each identified and reported lesion at baseline and during follow-up. Imaging based evaluation should always be done rather than clinical examination unless the lesion(s) being followed cannot be imaged but are assessable by clinical exam.

Clinical lesions: Clinical lesions will only be considered measurable when they are superficial and ≥10 mm diameter as assessed using calipers (e.g. skin nodules). For the case of skin lesions, documentation by color photography including a ruler to estimate the size of the lesion is suggested. As noted above, when lesions can be evaluated by both clinical exam and imaging, imaging evaluation should be undertaken since it is more objective and may also be reviewed at the end of the study.

Chest X-ray: Chest CT is preferred over chest X-ray, particularly when progression is an important endpoint, since CT is more sensitive than X-ray, particularly in identifying new lesions. However, lesions on chest X-ray may be considered measurable if they are clearly defined and surrounded by aerated lung. See Appendix II for more details.

CT, MRI: CT is the best currently available and reproducible method to measure lesions selected for response assessment. This guideline has defined measurability of lesions on CT scan based on the assumption that CT slice thickness is 5 mm or less. As is described in Appendix II, when CT scans have slice thickness greater than 5 mm, the minimum size for a measurable lesion should be twice the slice

thickness. MRI is also acceptable in certain situations (e.g. for body scans). More details concerning the use of both CT and MRI for assessment of objective tumor response evaluation are provided in Appendix II.

Ultrasound: Ultrasound is not useful in assessment of lesion size and should not be used as a method of measurement. Ultrasound examinations cannot be reproduced in their entirety for independent review at a later date and, because they are operator dependent, it cannot be guaranteed that the same technique and measurements will be taken from one assessment to the next. If new lesions are identified by ultrasound in the course of the study, confirmation by CT or MRI is advised. If there is concern about radiation exposure at CT, MRI may be used instead of CT in selected instances.

Endoscopy, laparoscopy: The utilization of these techniques for objective tumor evaluation is not advised. However, they can be useful to confirm complete pathological response when biopsies are obtained or to determine relapse in trials where recurrence following complete response or surgical resection is an endpoint.

Tumor markers: Tumor markers alone cannot be used to assess objective tumor response. If markers are initially above the upper normal limit, however, they must normalize for a patient to be considered in complete response (CR).

Cytology, histology: These techniques can be used to differentiate between PR and CR in rare cases if required by protocol (for example, residual lesions in tumor types such as germ cell tumors, where known residual benign tumors can remain). When effusions are known to be a potential adverse effect of treatment (e.g. with certain taxane compounds or angiogenesis inhibitors), the cytological confirmation of the neoplastic origin of any effusion that appears or worsens during treatment can be considered if the measurable tumor has met criteria for response or stable disease in order to differentiate between response (or stable disease) and progressive disease.

Tumor response evaluation

Assessment of overall tumor burden and measurable disease

To assess objective response or future progression, it is necessary to estimate the *overall tumor burden at baseline* and use this as a comparator for subsequent measurements. Measurable disease is defined by the presence of at least one measurable lesion

Baseline documentation of ‘target’ and ‘non-target’ lesions

When more than one measurable lesion is present at baseline all lesions up to a maximum of five lesions total (and a maximum of two lesions per organ) representative of all involved organs should be identified as *target lesions* and will be recorded and measured at baseline.

Target lesions should be selected on the basis of their size (lesions with the longest diameter), be representative of all involved organs, but in addition should be those that lend themselves to *reproducible repeated measurements*. It may be the case that, on occasion, the largest lesion does not lend itself to reproducible measurement in which circumstance the next largest lesion, which can be measured reproducibly, should be selected.

A sum of the diameters (longest for non-nodal lesions,) for all target lesions will be calculated and reported as the *baseline sum diameters*. The baseline sum diameters will be used as reference to further characterize any objective tumor regression in the measurable dimension of the disease.

All other lesions (or sites of disease) should be identified as *non-target lesions* and should also be recorded at baseline. Measurements are not required and these lesions should be followed as ‘present’, ‘absent’, or in rare cases ‘unequivocal progression. In addition, it is possible to record multiple nontarget

lesions involving the same organ as a single item on the case record form (e.g. 'multiple enlarged pelvic lymph nodes' or 'multiple liver metastases').

Response criteria

This section provides the definitions of the criteria used to determine objective tumour response for target lesions.

Evaluation of target lesions

Complete Response (CR): Disappearance of all target lesions.

Partial Response (PR): At least a 30% decrease in the sum of diameters of target lesions, taking as reference the baseline sum diameters.

Progressive Disease: At least a 20% increase in the sum of diameters of target lesions, taking as reference the smallest sum on study (this includes the baseline sum if that is the smallest on study). In addition to the relative increase of 20%, the sum must also demonstrate an absolute increase of at least 5 mm. (Note: the appearance of one or more new lesions is also considered progression).

Stable Disease (SD): Neither sufficient shrinkage to qualify for PR nor sufficient increase to qualify for progressive disease, taking as reference the smallest sum diameters while on study.

Evaluation of non-target lesions

Complete Response (CR): Disappearance of all non-target lesions and normalization of tumor marker level.

Non-CR/Non-progressive disease: Persistence of one or more non-target lesion(s) and/or maintenance of tumor marker level above the normal limits.

Progressive Disease: Unequivocal progression of existing non-target lesions. (*Note*: the appearance of one or more new lesions is also considered progression).

Evaluation of best overall response

The best overall response is the best response recorded from the start of the study treatment until the end of treatment taking into account any requirement for confirmation. On occasion a response may not be documented until after the end of therapy so protocols should be clear if post-treatment assessments are to be considered in determination of best overall response. The patient's best overall response assignment will depend on the findings of both target and non-target disease and will also take into consideration the appearance of new lesions.

Timepoint response

It is assumed that at each protocol specified timepoint, a response assessment occurs. Table 1 on the next page provides a summary of the overall response status calculation at each timepoint for patients who have measurable disease at baseline.

When patients have non-measurable (therefore non-target) disease only, Table 2 is to be used.

Best overall response: all timepoints

The *best overall response* is determined once all the data for the patient is known.

Best response determination in trials where confirmation of complete or partial response IS NOT required: Best response in these trials is defined as the best response across all timepoints (for example, a patient who has SD at first assessment, PR at second assessment, and progressive disease on last

assessment has a best overall response of PR). When SD is believed to be best response, it must also meet the protocol specified minimum time from baseline. If the minimum time is not met when SD is otherwise the best timepoint response, the patient's best response depends on the subsequent assessments. For example, a patient who has SD at first assessment, progressive disease at second and does not meet minimum duration for SD, will have a best response of progressive disease. The same patient lost to follow-up after the first SD assessment would be considered inevaluable.

Table 1 - Timepoint Response: Patients With Target (+/- Non-Target) Disease

Target lesions	Non-target lesions	New lesions	Overall response
CR	CR	No	CR
CR	Non-CR/non-PD	No	PR
CR	Not evaluated	No	PR
PR	Non-PD or not all evaluated	No	PR
SD	Non-PD or not all evaluated	No	SD
Not all evaluated	Non-PD	No	NE
PD	Any	Yes or No	PD
Any	PD	Yes or No	PD
Any	Any	Yes	PD

CR=complete response; PR=partial response; SD=stable disease; PD=progressive disease; NE=\inevaluable.

Table 2 - Timepoint Response: Patients With Non-Target Disease only

Non-target lesions	New lesions	Overall response
CR	No	CR
Non-CR/non-PD	No	Non-CR/non-PD[a]
Not all evaluated	No	NE
Unequivocal PD	Yes or No	PD
Any	Yes	PD

CR=complete response; PD=progressive disease; NE=inevaluable.

[a] Non-CR/non-PD' is preferred over 'stable disease' for non-target disease since SD is increasingly used as endpoint for assessment of efficacy in some trials so to assign this category when no lesions can be measured is not advised.

Patients with a global deterioration of health status requiring discontinuation of treatment without objective evidence of disease progression at that time should be reported as 'symptomatic deterioration'. Every effort should be made to document objective progression even after discontinuation of treatment. Symptomatic deterioration is not a descriptor of an objective response: it is a reason for stopping study therapy. The objective response status of such patients is to be determined by evaluation of target and non-target disease as shown in Tables 1–2.

Conditions that define 'early progression, early death and inevaluability' are study specific and should be clearly described in each protocol (depending on treatment duration, treatment periodicity).

In some circumstances it may be difficult to distinguish residual disease from normal tissue. When the evaluation of complete response depends upon this determination, it is recommended that the residual lesion be investigated (fine needle aspirate/biopsy) before assigning a status of complete response. FDG-PET may be used to upgrade a response to a CR in a manner similar to a biopsy in cases where a residual radiographic abnormality is thought to represent fibrosis or scarring. The use of FDG-PET in this circumstance should be prospectively described in the protocol and supported by disease specific medical literature for the indication. However, it must be acknowledged that both approaches may lead to false positive CR due to limitations of FDG-PET and biopsy resolution/ sensitivity.

For equivocal findings of progression (e.g. very small and uncertain new lesions; cystic changes or necrosis in existing lesions), treatment may continue until the next scheduled assessment. If at the next scheduled assessment, progression is confirmed, the date of progression should be the earlier date when progression was suspected.

Duration of overall response

The duration of overall response is measured from the time measurement criteria are first met for CR/PR (whichever is first recorded) until the first date that recurrent or progressive disease is objectively documented (taking as reference for progressive disease the smallest measurements recorded on study).

The duration of overall complete response is measured from the time measurement criteria are first met for CR until the first date that recurrent disease is objectively documented.

Duration of stable disease

Stable disease is measured from the start of the treatment (in randomized trials, from date of randomization) until the criteria for progression are met, taking as reference the *smallest sum on study* (if the baseline sum is the smallest, this is the reference for calculation of progressive disease).

The clinical relevance of the duration of stable disease varies in different studies and diseases. If the proportion of patients achieving stable disease for a minimum period of time is an endpoint of importance in a particular trial, the protocol should specify the minimal time interval required between two measurements for determination of stable disease.

Summary of major changes RECIST 1.0 to RECIST 1.1[a]

	RECIST 1.0	RECIST 1.1	Rationale
Minimum size measurable lesions	CT: 10 mm spiral; 20 mm non-spiral	CT 10 mm; delete reference to spiral scan	Most scans used have 5 mm or less slice thickness Clearer to give instruction based on slice interval if it is greater than 5 mm
	Clinical: 20 mm	Clinical: 10 mm (must be measurable with calipers)	Caliper measurement will make this reliable.
	Lymph node: not mentioned	CT:<br>≥15 mm short axis for target<br>≥10 - <15 mm for non-target<br><10 mm is non-pathological	Since nodes are normal structure need to define pathological enlargement. Short axis is most sensitive.
Special considerations on lesion measurability	-	Notes included on bone lesions, cystic lesions	Clarify frequently asked questions.
Overall tumour burden	10 lesions (5 per organ)	5 lesions (2 per organ)	Data warehouse analysis shows no loss of information if lesion number reduced from 10 to 5. A maximum of 2 lesions per organ yields sufficient representation per disease site.
Response criteria target disease	CR lymph node not mentioned	CR lymph nodes must be <10 mm short axis	In keeping with normal size of nodes.
	PD 20% increase over smallest sum on study or new lesions	PD 20% increase over smallest sum on study (including baseline if that is smallest) and at least 5 mm increase or new lesions	Clarification that if baseline measurement is smaller than any on study measurement, it is reference against which PD is assessed 5 mm absolute increase to guard against over calling PD when total sum is very small and 20% increase is within measurement error.

Summary of major changes RECIST 1.0 to RECIST 1.1[a]

	RECIST 1.0	RECIST 1.1	Rationale
Response criteria non-target disease	'unequivocal progression' considered as PD	More detailed description of 'unequivocal progression' to indicate that it should not normally trump target disease status. It must be representative of overall disease status change, not a single lesion increase	Confusion with RECIST 1.0 where some were considering PD if 'increase' in any non-target lesion, even when target disease is stable or responding.
New lesions		New section on New lesions	To provide guidance on when a lesion is considered new (and thus PD).
Overall response	Table integrated target and non-target lesions	Two tables: one integrating target and non-target and the other of non-target only	To account for the fact that RECIST criteria are now being used in trials where PFS is the endpoint and not all patients have measurable (target) disease at baseline.
		Special notes:<br>How to assess and measure lymph nodes;<br>CR in face of residual tissue;<br>Discussion of 'equivocal' progression	Frequently asked questions on these topics.
Confirmatory measure	For CR and PR: criteria must be met again 4 weeks after initial documentation	Retain this requirement ONLY for non-randomised trials with primary endpoint of response	Data warehouse shows that response rates rise when confirmation is eliminated, but the only circumstance where this is important is in trials where there is no concurrent comparative control and where this measure is the primary endpoint.
Progression-free survival	General comments only	More specific comments on use of PFS (or proportion progression-free) as phase II endpoint.<br>Greater detail on PFS assessment in phase III trials	Increasing use of PFS in phase III trials requires guidance on assessment of PD in patients with non-measurable disease
Reporting of response results	9 categories suggested for reporting phase II results	Divided into phase II and phase III;<br>9 categories collapsed into 5;<br>In phase III, guidance given about reporting response	Simplifies reporting and clarifies how to report phase II and III data consistently.
Response in phase III trials	More relaxed guidelines possible if protocol specified	This section removed and referenced in section above: no need to have different criteria for phase II and III	Simplification of response assessment by reducing number of lesions and eliminating need for confirmation in randomized studies where response is not the primary endpoint makes separate 'rules' unnecessary.

Summary of major changes RECIST 1.0 to RECIST 1.1[a]

	RECIST 1.0	RECIST 1.1	Rationale
Imaging appendix	Appendix I	Appendix II: updated with detailed guidance on use of MRI, PET/CT.<br>Other practical guidance included	Evolving use of newer modalities addressed.<br>Enhanced guidance in response to frequent questions and from radiology review experience.
New appendices		Appendix I: comparison of RECIST 1.0 and 1.1;<br>Appendix III: frequently asked questions	

PD=progressive disease
[a] See publication for references applicable to this table.

Attachment 3: EORTC QLQ – OV28

EORTC QLQ - OV28

Patients sometimes report that they have the following symptoms or problems. Please indicate the extent to which you have experienced these symptoms or problems during the past week.

During the past week:	Not at All	A Little	Quite a Bit	Very Much
31. Did you have abdominal pain?	1	2	3	4
32. Did you have a bloated feeling in your abdomen / stomach?	1	2	3	4
33. Did you have problems with your clothes feeling too tight?	1	2	3	4
34. Did you experience change in bowel habit as a result of your disease or treatment?	1	2	3	4
35. Were you troubled by passing wind / gas / flatulence?	1	2	3	4
36. Have you felt full up too quickly after beginning to eat?	1	2	3	4
37. Have you had indigestion or heartburn?	1	2	3	4
38. Have you lost any hair?	1	2	3	4
39. Answer this question only if you had any hair loss: Were you upset by the loss of your hair?	1	2	3	4
40. Did food and drink taste different from usual?	1	2	3	4
41. Have you had tingling hands or feet?	1	2	3	4
42. Have you had numbness in your fingers or toes?	1	2	3	4
43. Have you felt weak in your arms or legs?	1	2	3	4
44. Did you have aches or pains in your muscles or joints?	1	2	3	4
45. Did you have problems with hearing?	1	2	3	4
46. Did you urinate frequently?	1	2	3	4
47. Have you had skin problems (e.g. itchy, dry)?	1	2	3	4
48. Did you have hot flushes?	1	2	3	4
49. Did you have night sweats?	1	2	3	4

Specimen

Please go on to next page

**During the past week:**	**Not at All**	**A Little**	**Quite a Bit**	**Very Much**
50. Have you felt physically less attractive as a result of your disease or treatment?	1	2	3	4
51. Have you been dissatisfied with your body?	1	2	3	4
52. How much has your disease been a burden to you?	1	2	3	4
53. How much has your treatment been a burden to you?	1	2	3	4
54. Were you worried about your future health?	1	2	3	4

**During the past 4 weeks:**	**Not at All**	**A Little**	**Quite a Bit**	**Very Much**
55. To what extent were you interested in sex?	1	2	3	4
56. To what extent were you sexually active?	1	2	3	4

Answer the following two questions only if you were sexually active:

57. To what extent was sex enjoyable for you?	1	2	3	4
58. Did you have a dry vagina during sexual activity?	1	2	3	4

Specimen

© Copyright 1997 EORTC Quality of Life Group. All rights reserved. (Validated module)

Attachment 4: EQ–5D-5L Health Questionnaire

EQ-5D-5L

Health Questionnaire

English version for the USA

USA (English) © 2009 EuroQol Group. EQ-5D™ is a trade mark of the EuroQol Group

Under each heading, please check the ONE box that best describes your health TODAY

MOBILITY

- I have no problems walking ❑
- I have slight problems walking ❑
- I have moderate problems walking ❑
- I have severe problems walking ❑
- I am unable to walk ❑

SELF-CARE

- I have no problems washing or dressing myself ❑
- I have slight problems washing or dressing myself ❑
- I have moderate problems washing or dressing myself ❑
- I have severe problems washing or dressing myself ❑
- I am unable to wash or dress myself ❑

USUAL ACTIVITIES *(e.g. work, study, housework, family or leisure activities)*

- I have no problems doing my usual activities ❑
- I have slight problems doing my usual activities ❑
- I have moderate problems doing my usual activities ❑
- I have severe problems doing my usual activities ❑
- I am unable to do my usual activities ❑

PAIN / DISCOMFORT

- I have no pain or discomfort ❑
- I have slight pain or discomfort ❑
- I have moderate pain or discomfort ❑
- I have severe pain or discomfort ❑
- I have extreme pain or discomfort ❑

ANXIETY / DEPRESSION

- I am not anxious or depressed ❑
- I am slightly anxious or depressed ❑
- I am moderately anxious or depressed ❑
- I am severely anxious or depressed ❑
- I am extremely anxious or depressed ❑

USA (English) © 2009 EuroQol Group. EQ-5D™ is a trade mark of the EuroQol Group

- We would like to know how good or bad your health is TODAY.
- This scale is numbered from 0 to 100.
- 100 means the <u>best</u> health you can imagine.
  0 means the <u>worst</u> health you can imagine.
- Mark an X on the scale to indicate how your health is TODAY.
- Now, please write the number you marked on the scale in the box below.

YOUR HEALTH TODAY = ☐

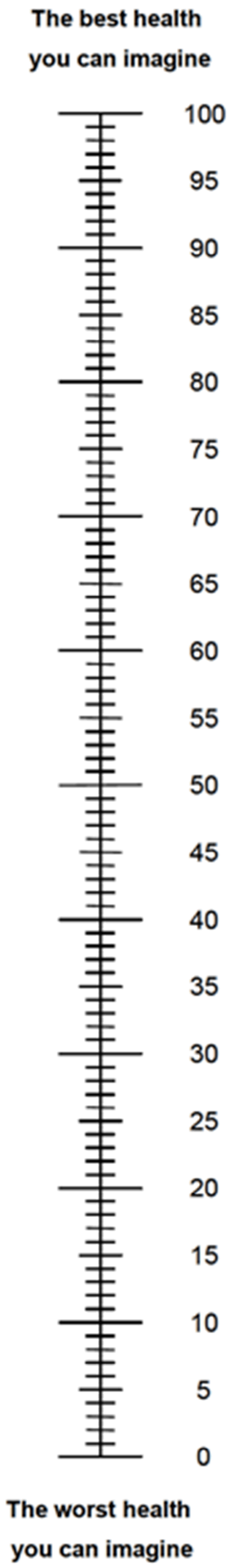

USA (English) © 2009 EuroQol Group. EQ-5D™ is a trade mark of the EuroQol Group

Attachment 5: New York Heart Association Classification

CLASS I: Subjects in whom angina is provoked by strenuous exertion.
Subjects with cardiac disease but without resulting limitations of physical activity. Ordinary physical activity does not cause undue fatigue, palpitations, dyspnea, or anginal pain.

CLASS II: Subjects with cardiac disease resulting in slight limitation of physical activity. They are comfortable at rest. Ordinary physical activity results in fatigue, palpitation, dyspnea, or anginal pain.

CLASS III: Subjects with cardiac disease resulting in marked limitation of physical activity. They are comfortable at rest. Less than ordinary physical activity causes fatigue, palpitation, dyspnea, or anginal pain.

CLASS IV: Subjects with cardiac disease resulting in inability to carry on any physical activity without discomfort. Symptoms of cardiac insufficiency or of the anginal syndrome may be present even at rest.

Attachment 6: Cockcroft-Gault Method

Creatinine clearance (CrCl) is to be estimated by using the following formula which is specific for females:

$$GFR = \frac{(140\text{-age}) \times (\text{Weight measured in kg}) \times (0.85)}{(72 \times \text{serum creatinine measured in mg/deciliter})}$$

GFR=glomerular filtration rate

Attachment 7: Study Procedures as Outlined in Amendment 6

Independent Data Monitoring Committee

On 26 June 2017, the IDMC requested a futility analysis of OS to be performed at the time of the next meeting 6 months later. In view of the IDMC request, study data up to 20 September 2017 were evaluated for safety and OS (45% rather than 60% of the required death events at the planned interim analysis). Following the review of the study data by the IDMC on 15 December 2017, the HR for OS was 0.962, crossing the previously agreed upon threshold for futility of 0.93. In view of this result and the observed and expected higher toxicity in Arm A as compared with Arm B, the IDMC recommended discontinuing the study.

Subjects Assigned to Treatment Arm A and Treatment Arm B

As of Amendment 6, treatment with trabectedin should be immediately discontinued for subjects assigned to Arm A (trabectedin+DOXIL). All study subjects (Arm A or Arm B) currently on study who, in the opinion of the investigator, are deriving clinical benefit may continue treatment with single-agent DOXIL as per the local standard of care. Treatment for these subjects may continue as long as the subjects comply with protocol-specified prohibitions and restrictions for treatment (Section 4.3) and as long as they experience clinical benefit in the opinion of the investigator. DOXIL will be provided by JR&D as needed until post-trial access to DOXIL is available or until disease progression, whichever occurs first.

Accordingly, the end of study data collection is defined as when all subjects on study treatment have completed treatment termination visit assessments as specified in the Time and Events Schedule for Amendment 6 or by 18 January 2018, whichever occurs first. For subjects continuing treatment with single-agent DOXIL, as per the local standard of care, only serious adverse events should be reported to JR&D as outlined in Section 12.3.2.

Subjects in Screening

No new subjects will be screened. Subjects currently in screening are be eligible to receive treatment with single-agent DOXIL, if the investigator confirms that such treatment would be a clinically appropriate therapeutic option. These subjects will not be randomized to a treatment group, but must meet study inclusion and exclusion criteria (per Amendment 6, Sections 4.1 and 4.2). As indicated above, for subjects in treatment Arms A and B who continue on single-agent DOXIL, subjects in screening who receive single-agent DOXIL will be allowed to continue treatment as long as the subjects comply with protocol-specified prohibitions and restrictions for treatment (Section 4.3) and who, in the opinion of the investigator, are experiencing clinical benefit. DOXIL will be provided by JR&D as needed until post-trial access to DOXIL is available or until disease progression, whichever occurs first.

Data Collection

Following Amendment 6, study data collection will cease when all subjects on study treatment have completed the treatment termination visit assessments as specified in the Time and Events Schedule for Amendment 6 or by 18 January 2018, whichever occurs first. Data collection for adverse events or abnormal findings will be monitored until the study clinical cutoff date (18 January 2018).

For subjects continuing treatment with single-agent DOXIL, as per the local standard of care, only serious adverse events should be reported to JR&D as outlined in Section 12.3.2.

INVESTIGATOR AGREEMENT

YONDELIS® (trabectedin)

Clinical Protocol ET743-OVC-3006 – Amendment 6

INVESTIGATOR AGREEMENT

I have read this protocol and agree that it contains all necessary details for carrying out this study. I will conduct the study as outlined herein and will complete the study within the time designated.

I will provide copies of the protocol and all pertinent information to all individuals responsible to me who assist in the conduct of this study. I will discuss this material with them to ensure that they are fully informed regarding the study drug, the conduct of the study, and the obligations of confidentiality.

Coordinating Investigator (where required):

Name (typed or printed): ____________________

Institution and Address: ____________________

Signature: ____________________ Date: ____________________
(Day Month Year)

Principal (Site) Investigator:

Name (typed or printed): ____________________

Institution and Address: ____________________

Telephone Number: ____________________

Signature: ____________________ Date: ____________________
(Day Month Year)

Sponsor's Responsible Medical Officer:

Name (typed or printed) [redacted] Andrew Cakana, MD

Institution: [redacted] Janssen Research & Development

Signature: [redacted] Date: 11/01/18
(Day Month Year)

Note: If the address or telephone number of the investigator changes during the course of the study, written notification will be provided by the investigator to the sponsor, and a protocol amendment will not be required.

118

Approved, 9 January 2018
